# Elucidation of the Physical Separation and Accumulation Mechanisms of Three Distinct Layers in Grease Traps

**DOI:** 10.1002/wer.70511

**Published:** 2026-07-29

**Authors:** Ling Ying Tang, Alex Kwong Jun Kiu, Ngie Hing Wong, Chung Siung Choo, Lily Li, Chin Ping Tan, Abu Zahrim Yaser, Deni Shidqi Khaerudini, Jaka Sunarso

**Affiliations:** ^1^ Research Centre for Sustainable Technologies, Faculty of Engineering, Computing and Science Swinburne University of Technology Kuching Sarawak Malaysia; ^2^ Centre for Innovative Society, Faculty of Engineering, Computing, and Science Swinburne University of Technology Kuching Sarawak Malaysia; ^3^ Centre of Smart Infrastructure and Digital Construction, Department of Civil and Construction Engineering Swinburne University of Technology Victoria Australia; ^4^ Department of Food Technology, Faculty of Food Science and Technology Universiti Putra Malaysia Serdang Selangor Malaysia; ^5^ Faculty of Engineering Universiti Malaysia Sabah Kota Kinabalu Sabah Malaysia; ^6^ Research Center for Energy Conversion Technology National Research and Innovation Agency (BRIN), Bld. 625 National Science Techno Park BJ Habibie South Tangerang Banten Indonesia

**Keywords:** Column tests, Floating and settling velocity, FOG deposits, Grease Trap, Hydraulic retention time, Surface overflow rate

## Abstract

Grease traps are essential for intercepting fats, oils, and grease (FOG) to prevent sewer blockages. However, the internal mechanisms governing their separation efficiency and layer accumulation remain poorly defined. This work investigates the dynamics of three distinct internal phases inside grease traps, i.e., top‐floated scum, middle‐clarified liquid, and bottom‐settled sludge, through a 32‐day site investigation (SI) and 24 laboratory column tests (CTs). The SI revealed that the settled sludge layer was 10 times thicker at the inlet chamber and 72 times thicker at the outlet chamber than the floated scum, indicating that sludge accumulation is the primary driver of desludging frequency and a key indirect influence on grease trap performance. CT results further demonstrated that separation kinetics were governed by solids content, with low‐solid wastewater achieving settling velocities of up to 3.04 m h^−1^, significantly higher than those of high‐solid wastewater (0.87 m h^−1^). These differences highlight the solid content in the grease trap governing the separation rates. Based on these findings, this work proposes optimized design criteria, including a surface overflow rate (SOR) of 20.9 m^3^ d^−1^ m^−2^, a minimum surface area (*A*_*s*_) of 1.25 m^2^, and a 1‐h hydraulic retention time (HRT). Ultimately, these results provide a systematic assessment framework to refine grease‐trap design standards based on the physical behavior of all three internal waste layers.

## Summary

- Six separation mechanisms control the three‐layer stratification in grease traps.
- Sludge accumulation was up to 9.3 × higher than surface scum in field grease traps.
- Phase separation kinetics strongly depend on total solids loading conditions.
- High‐solid wastewater prolonged phase separation and reduced settling efficiency.
- Effective grease traps need at least 20.9 m^3^ d^−1^ m^−2^ SOR and 1 h HRT.

## Graphical abstract

Grease trap performance is dominated by sludge accumulation rather than surface scum. This study reveals the mechanisms governing three‐layer formation and provides design criteria and a mechanistic model to improve grease trap efficiency and prevent sewer blockages.

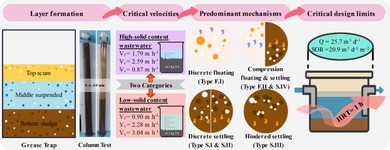

## 1 Introduction

Within a grease trap, kitchen wastewater separates into three distinct layers: top‐floated scum, middle‐clarified/suspended liquid, and bottom‐settled sludge, each exhibiting unique physicochemical characteristics (Aziz et al. 2012; Tang et al. 2024). For instance, the top scum layer is characterized by higher fat, oil, and grease (FOG, 0.266 g g^−1^) and volatile solids (VS, 0.134 g g^−1^), yet lower total solids (TS, 0.149 g g^−1^) than the bottom sludge layer. In contrast, the bottom layer contains negligible FOG but higher TS (0.225 g g^−1^) and lower VS (0.096 g g^−1^) (Tang et al. 2024). The middle liquid layer maintains a stable, high‐turbidity suspension with significant total suspended solids (TSS, 5.20 g L^−1^) (Tang et al. 2024). This suggests that substantial amounts of TSS and FOG deposits escape into public sewers, where they disrupt wastewater flow and compromise the efficiency of downstream treatment plants (Omidvar et al. 2023; Ahmad et al. 2023; Hums et al. 2016). These compositional variations point to divergent physicochemical mechanisms: the top and bottom layers are governed by the saponification, crystallization, and aggregation of free fatty acids (FFAs) with minerals (He et al. 2013; Gross et al. 2017; Iasmin et al. 2016), while the middle layer is dominated by the emulsification of FOG and FFAs driven by surfactants (Del Mundo and Sutheerawattananonda 2017; Wong et al. 2007). Despite the potential to repurpose scum and sludge as cost‐effective feedstocks (Tran et al. 2021; Hums et al. 2018), the physical separation and accumulation dynamics of these three layers remain poorly understood. This ambiguity results in inconsistent grease‐trap design and operational guidelines globally.

As summarized in Table 1, commercial grease‐trap designs vary significantly in terms of materials, geometries, volumes, and hydraulic retention times (HRTs). The HRT represents the detention time of wastewater within the unit and is defined as the ratio of the working volume (*V*) to the influent flow rate (*Q*), i.e., HRT = *V*/*Q*. A higher HRT facilitates the distinct separation and accumulation of the three internal layers. Conversely, a truncated HRT results in incomplete separation, allowing FOG deposits and solid residues to bypass the grease trap and enter the sewer network. Consequently, a robust grease‐trap design must account for the floating and settling velocities of waste particles relative to the surface overflow rate (SOR). The SOR is defined as the influent flow rate per unit of surface area (*A*_*s*_), i.e., SOR = *Q*/*A*_*s*_ (Tchobanoglous et al. 2014). From a fluid dynamics perspective, if the floating or settling velocity of a particle is lower than the SOR, the surface area (*A*_*s*_) is insufficient for that particle to be successfully captured. Currently, commercial grease traps exhibit inconsistent HRT and SOR values (Table 1), which obscures the correlation between design parameters and separation efficiency. Therefore, characterizing the relationship between wastewater properties and particle kinetics is essential for establishing standardized HRT and SOR guidelines that optimize grease‐trap performance.

**TABLE 1:** Summary of grease trap design criteria and technical specifications across selected countries.

Country	Material	Shape	Dimension (mm)	Volume (L)	HRT (h)	Ref.
Sarawak, Malaysia	High‐density polyethylene (HDPE)	Circular	Ø994	500	0.50–1.00	**This work** (WEIDA Integrated Industries Sdn Bhd 2021)
Australia	Reinforced concrete with stainless‐steel cover	Rectangular	L1800 × W1000 × H900	> 1000	1	(Australian Building Codes Board 2019; Federation Council 2026)
HK, China	Reinforced concrete	Rectangular	L2000 × W2000 × H1800	> 250	0.33	(Environmental Protection Department 2022)
Indonesia	Reinforced concrete	Rectangular	L800 × W400 × H1000	256	3.10	(Purwanti et al. 2018)
Italy	Polyethylene (PE)	Circular	Ø1050	1100	0.25	(Stampaggio Materie Plastiche 2022)
Singapore	Reinforced concrete with cement and stainless‐steel strainer basket	Circular	Ø2060	> 1000	> 0.50	(Singapore's National Water Agency 2019; British Standards Institution 2004)
United States	Reinforced concrete	Rectangular	L3200 × W1524 × H1524	3785	6.68	(Wong et al. 2007; Wong 1998)
United States	Reinforced concrete	Rectangular	L4876 × W1219 × H1473	7570	17.3	(Wong et al. 2007; Wong 1998)
United States	Reinforced concrete	Rectangular	L4572 × W2438 × H1828	8706	20.6	(Wong et al. 2007; Wong 1998)
United States	Reinforced concrete	Rectangular	L3200 × W1524 × H1524	20,441	18.9	(Wong et al. 2007; Wong 1998)

Dietary variations significantly alter the chemical composition of grease‐trap wastewater, specifically regarding FFAs, surfactants, and minerals/metals. These components dictate the dynamics of the three‐layer formation by influencing the floating and settling velocities of suspended particles (Aziz et al. 2011; Gurd et al. 2019; Tang et al. 2024). Consequently, these varying separation and accumulation mechanisms directly impact the HRT required for effective design and operation. When the HRT is insufficient, suspended solids fail to separate and are instead transported through baffle openings (Del Mundo and Sutheerawattananonda 2017; Wong et al. 2007), leading to the accumulation of FOG deposits in downstream sewer networks (He et al. 2017). Despite the critical nature of these kinetics, research concurrently investigating both floating and settling behaviors to optimize grease‐trap performance remains limited.

Tadros (2013) and Wang et al. (2015) proposed several mechanisms for the breakdown and accumulation of emulsified oily wastewater, in which floating pathways are determined by specific particle interactions (Figure S1). These include creaming, where discrete FOG droplets rise independently due to density differentials; coalescence, where droplets merge to increase buoyancy; flocculation, where droplets aggregate and may eventually settle if they bind with dense suspended solids (Sultana et al. 2025); and entrainment, where surfactant‐stabilized air bubbles promote the flotation of trapped solids.

Under grease‐trap conditions, these processes can be generalized into two functional categories: discrete floating (**Type F.I**), characterized by individual particles in the middle liquid layer rising independently, and compression floating (**Type F.II**), where the top scum layer compacts under its own accumulated mass, potentially leading to settling if the aggregate density exceeds that of the surrounding wastewater. Drawing on the fundamental similarities between grease traps and municipal wastewater, the settling mechanisms in grease traps can be categorized into four distinct types (D'Amato 2008; Ekama 1997; van Loosdrecht et al. 2016). Discrete non‐flocculent settling (**Type S.I**) occurs at low solid concentrations, where particles settle independently without interaction. Discrete flocculent settling (**Type S.II**) involves low‐concentration particles that aggregate into larger flocs, thereby increasing their mass and settling velocity. As solid concentrations increase, hindered settling (**Type S.III**) predominates; here, interparticle forces cause the solids to settle as a single unit at a uniform velocity, creating a distinct solid–liquid interface below a clear supernatant. Lastly, compression settling (**Type S.IV**) occurs as the accumulated sludge layer consolidates due to continuous mass addition, with increased physical contact and overlaying significantly reducing the settling velocity. In short, these flotation and settling mechanisms govern the separation and accumulation velocity variation of FOG, deposits, and other particulates during the three‐layer formation, thereby determining the optimal HRT and SOR required for effective grease trap design and operation.

Research indicates that the concentrations of FFAs and minerals in grease‐trap wastewater significantly enhance separation and accumulation kinetics (Iasmin et al. 2014; Yousefelahiyeh et al. 2017). Higher FFA concentrations generally facilitate the formation of larger droplets, which increases their floating velocity (Stewart and Arnold 2011; Sakeena et al. 2011). Similarly, in municipal wastewater contexts, increasing solid concentrations can transition a system into hindered settling; while close particle proximity initially governs this phase, the collective increase in floc mass can ultimately accelerate settling velocities (van Loosdrecht et al. 2016). Conversely, emulsified FOG creates a stable suspension that significantly reduces both floating and settling velocities, thereby prolonging the time required for the three‐layer formation. Such delayed separation necessitates a lower SOR, i.e., a critical design parameter in clarifiers that ensures sufficient time for solids to separate from the liquid phase. Because the separation and accumulation rates of FOG deposits and other residues are entirely dependent on these floating and settling velocities, they directly dictate the required HRT and SOR. Consequently, a systematic investigation of these floating and settling behaviors is essential to establish standardized HRT and SOR values for optimizing grease‐trap design and operational efficiency.

This work investigates the physical separation and accumulation mechanisms of the three distinct phases formed within grease traps, i.e., top‐floated scum, middle‐clarified liquid, and bottom‐settled sludge, by integrating field data with controlled laboratory analysis. The research was executed in three stages: a 32‐day site investigation (SI) of four grease traps in a major community market to establish longitudinal thickness profiles, followed by 24 laboratory column tests (CTs) using 16 field samples to quantify floating‐settling velocities and separation‐accumulation rates, and lastly, a data synthesis phase to determine optimal HRT and SOR limits.

## 2 Materials and Methods

### 2.1 Materials

Building on the methodology established by Tang et al. (2024), three custom‐designed samplers were employed to monitor and collect waste from the four grease traps during the SI and subsequent CTs (Figure S2). During the SI, Sampler A was utilized to measure the thickness profiles of the three internal layers at designated points within both the inlet and outlet chambers (Figure S3). For the CTs, comprehensive samples of floated scum, clarified liquid, and settled sludge were collected using Samplers B and C, transported to the Swinburne Laboratory, and homogenized. The laboratory evaluations utilized an adapted sedimentation apparatus (SOLTEQ, Model TR01, Malaysia) to concurrently monitor floating and settling kinetics (Figure S4) (Torfs et al. 2016). Following each CT, samples were extracted from the newly separated top, middle, and bottom layers and characterized according to Standard Methods (Table 2). All experiments were performed in triplicate, and results were presented as mean ± standard deviation. Distilled water was utilized for all analytical characterizations to ensure consistency.

**TABLE 2:** Standard methods and the corresponding equipment/apparatus characterizing the collected samples.

Parameter	Unit	Equipment	Methods [Ref.]
pH	—	Eutech pH 150 meter (USA)	—
Temperature	°C	Thermometer	—
Electrical conductivity (EC)	μS cm^−1^	Eutech CON150 meter (USA)	APHA 2510B Baird et al. 2017
Total solids (TS)	g g^−1^	Memmert UF260Plus oven, Carbolite Gero AAF 1100	APHA 2540B Baird et al. 2017
Volatile solids (VS)	g g^−1^	Memmert UF260Plus oven, Carbolite Gero AAF 1100	APHA 2540E Baird et al. 2017
Fixed solids (FS)	g g^−1^	Memmert UF260Plus oven, Carbolite Gero AAF 1100	APHA 2540E Baird et al. 2017
Total suspended solids (TSS)	g L^−1^	Memmert UF260Plus oven, Carbolite Gero AAF 1100, Sartorius (Germany) Glass Microfiber Filter	APHA 2540D Baird et al. 2017
Volatile suspended solids (VSS)	g L^−1^	Memmert UF260Plus oven, Carbolite Gero AAF 1100	APHA 2540E Baird et al. 2017
Total dissolved solids (TDS)	g L^−1^	Memmert UF260Plus oven, vacuum filtration set	APHA 2540C Baird et al. 2017
Oil and grease (O&G)	mg L^−1^	Partition‐gravimetric method	APHA 5520B Baird et al. 2017
Fat, oil, and grease (FOG)	g g^−1^	Memmert UF260Plus oven, stainless steel strainers, and glass beakers	—

### 2.2 Site Investigations

Building on the methodology established by Tang et al. (2024), four grease traps serving 61 food and beverage stalls at a two‐story community wet market in Kuching (Sarawak, Malaysia) were selected for study. During the 32‐day SI, Sampler A was carefully immersed in each grease trap to assess the three‐layer formation without disturbing the wastewater. A 5‐min stabilization period was allowed for separation and accumulation before recording the layer thicknesses (mm). Each grease trap was profiled at three randomized locations within the inlet chamber; however, due to spatial constraints, only one profiling point was used for the outlet chamber (Figure S3). Although Grease Traps A and B were desludged on Day 17, and Grease Traps C and D on Day 18, daily monitoring continued through the subsequent period to maintain the continuity of SI. Lastly, the daily average thickness of each layer was calculated and normalized to the total liquid depth (%) using Equations ((1), (3)).

(1) $$\begin{array}{c} \mathrm{Top}\;\left(T\right)\;\text{layer composition}\;\left(\%\right)\\ =\frac{\text{Scum layer},T\;\left(\mathrm{mm}\right)}{\text{Total liquid layer thickness},D\;\left(\mathrm{mm}\right)}\times 100\% \end{array}$$

(2) $$\begin{array}{c} \text{Middle}\;\left(M\right)\;\text{layer composition}\;\left(\%\right)\\ =\frac{\text{Middle layer},\;M\;\left(\mathrm{mm}\right)}{\text{Total liquid layer thicknes}s,D\;\left(\mathrm{mm}\right)}\times 100\% \end{array}$$

(3) $$\begin{array}{c} \text{Bottom}\;\left(B\right)\;\text{layer composition}\;\left(\%\right)\\ =\frac{\text{Sludge layer},B\;\left(\mathrm{mm}\right)}{\text{Total liquid layer thickness},D\;\left(\mathrm{mm}\right)}\times 100\% \end{array}$$

### 2.3 Column Tests

To address the time constraints observed during the SI (where layer formation was limited to < 5 min), two sampling rounds (R1 and R2) were conducted to collect 16 samples over eight randomized days. These samples were used for 24 CTs under controlled laboratory conditions with an extended 60‐min duration. Each grease trap was sampled twice on different days to ensure data representativeness. During collection, Sampler B was employed specifically for the top scum layer, while Sampler C was used to retrieve the middle clarified liquid and bottom sludge layers from both the inlet and outlet chambers. Due to the limited sample *V* available from the outlet chambers (Figure S3), only one CT (C1) was performed for each outlet sample, whereas the larger inlet chamber samples underwent duplicate testing (C1 and C2). The sample labeling for the 24 CTs is summarized in Table 3. For instance, samples collected during the first and second rounds (R1 and R2) at the inlet (I) and outlet (O) chambers of Grease Traps A through D were designated with codes ranging from A‐R1‐I‐C1 to D‐R2‐O‐C2, where C1 and C2 represent the initial and repeated CTs for the same inlet chamber sample, respectively.

**TABLE 3:** Summary of sample identification and testing rounds for inlet (I) and outlet (O) chamber wastewater across four grease traps.

Chamber	Sampling	CT	Grease Trap A	Grease Trap B	Grease Trap C	Grease Trap D
Inlet (I)	R1	C1	A‐R1‐I‐C1	B‐R1‐I‐C1	C‐R1‐I‐C1	D‐R1‐I‐C1
		C2	A‐R1‐I‐C2	B‐R1‐I‐C2	C‐R1‐I‐C2	D‐R1‐I‐C2
	R2	C1	A‐R2‐I‐C1	B‐R2‐I‐C1	C‐R2‐I‐C1	D‐R2‐I‐C1
		C2	A‐R2‐I‐C2	B‐R2‐I‐C2	C‐R2‐I‐C2	D‐R2‐I‐C2
Outlet (O)	R1	C1	A‐R1‐O‐C1	B‐R1‐O‐C1	C‐R1‐O‐C1	D‐R1‐O‐C1
	R2	C1	A‐R2‐O‐C1	B‐R2‐O‐C1	C‐R2‐O‐C1	D‐R2‐O‐C1

**FIGURE 1:**
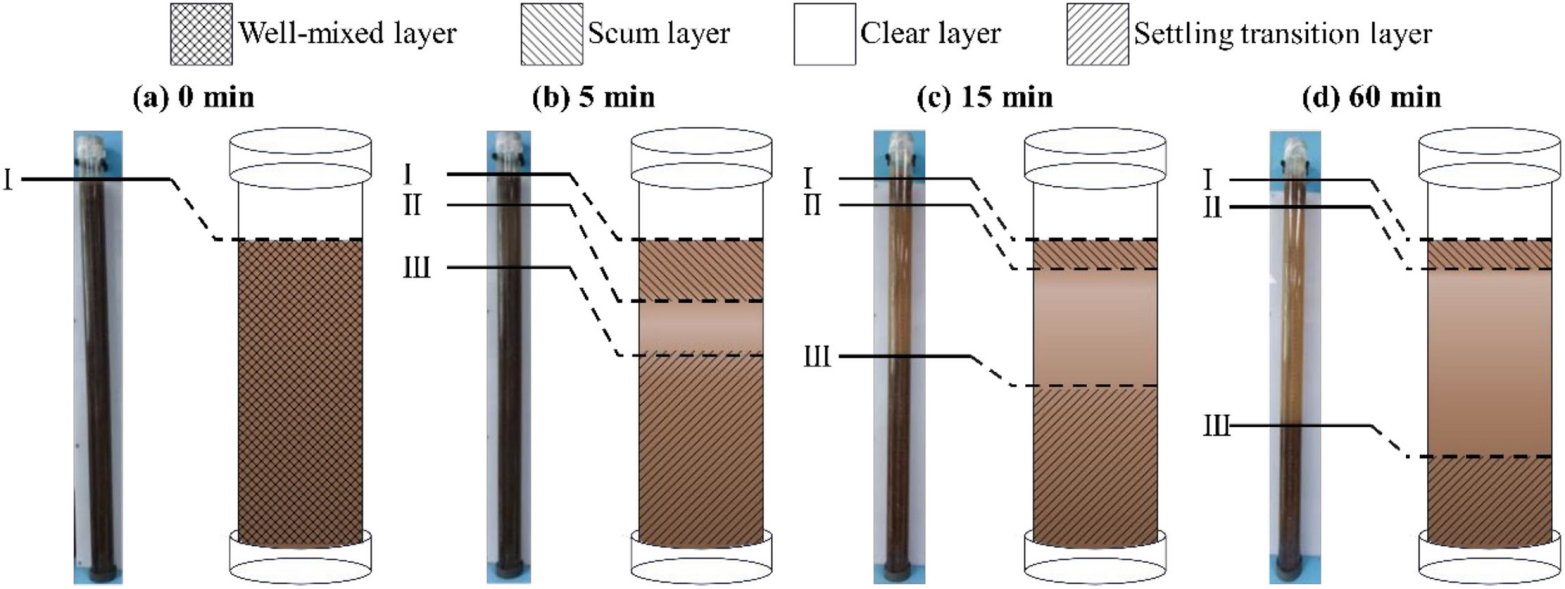
Schematic representation of the column test for sample B‐R1‐I‐C1, illustrating the progression of **Interface I** (total liquid), **Interface II** (floated scum), and **Interface III** (settling transition layer) at (a) 0, (b) 5, (c) 15, and (d) 60 min.

Figure 1 presents a schematic of the CTs using sample B‐R1‐I‐C1 to illustrate the observed layer formation and floating‐settling kinetics over 60 min. Unlike conventional sedimentation tests, this apparatus was modified to evaluate floatation and sedimentation behaviors simultaneously. Each composite sample, comprising scum, clarified liquid, and sludge, was transferred to the column (Figure S4) and homogenized by inverting the apparatus 15 times before the test commenced. At *t* = 0 (Figure 1), the initial clarified liquid interface (**Interface I**) was established, followed by the continuous recording of the floating scum interface (**Interface II**) and the settling sludge interface (**Interface III**) at 1‐min intervals. Following the 60‐min test period, the three distinct layers were extracted for physiochemical characterization according to Standard Methods (Table 2), with FOG content in the scum and sludge quantified using the modified TS extraction method (Tang et al. 2024). All experiments were performed in triplicate, and results were presented as mean ± standard deviation.

For each CT result, a layer thickness‐time graph was plotted to identify the steepest gradient, representing the maximum floating and settling velocities as defined by Equations (4) and (5). The systematic calculation procedure involved: (a) determining the thickness changes between specific time intervals *n*_*1*_ and *n*_*2*_ (the effective HRT); (b) calculating velocities where *A* represents the height difference between the liquid and scum interfaces, *B* represents the sludge height, and Δ*t* is the elapsed time; (c) identifying the critical velocity by comparing floating and settling rates; (d) calculating the SOR via Equation (6); and (e) determining the required grease‐trap surface area (*A*_*s*_) using the influent flow rate (*Q*) in Equation (7). Lastly, these calculated SOR, *A*_*s*_, and HRT limits were compared against the specifications of the four investigated grease traps and broader commercial designs to evaluate their adequacy in meeting fundamental separation requirements.

(4) $$\text{Floating velocity},m\;s-1=\frac{A_{t=n2}-A_{t=n1}}{t}$$

(5) $$\text{Settling velocity},m\;s-1=\frac{B_{t=n1}-B_{t=n2}}{t}$$

(6) $$\begin{array}{c} \text{Surface overflow rate}\;\left(\mathrm{SOR}\right),m3\;d-1\;m-2\\ =\text{Critical velocity},m\;h^{-1}\times 24\;h \end{array}$$

(7) $$\text{Required surface area}\;\left(\mathrm{As}\right),m2=\frac{Q}{\mathrm{SOR}}$$

## 3 Results and Discussion

### 3.1 Three‐Layer Thickness Profiles

**FIGURE 2:**
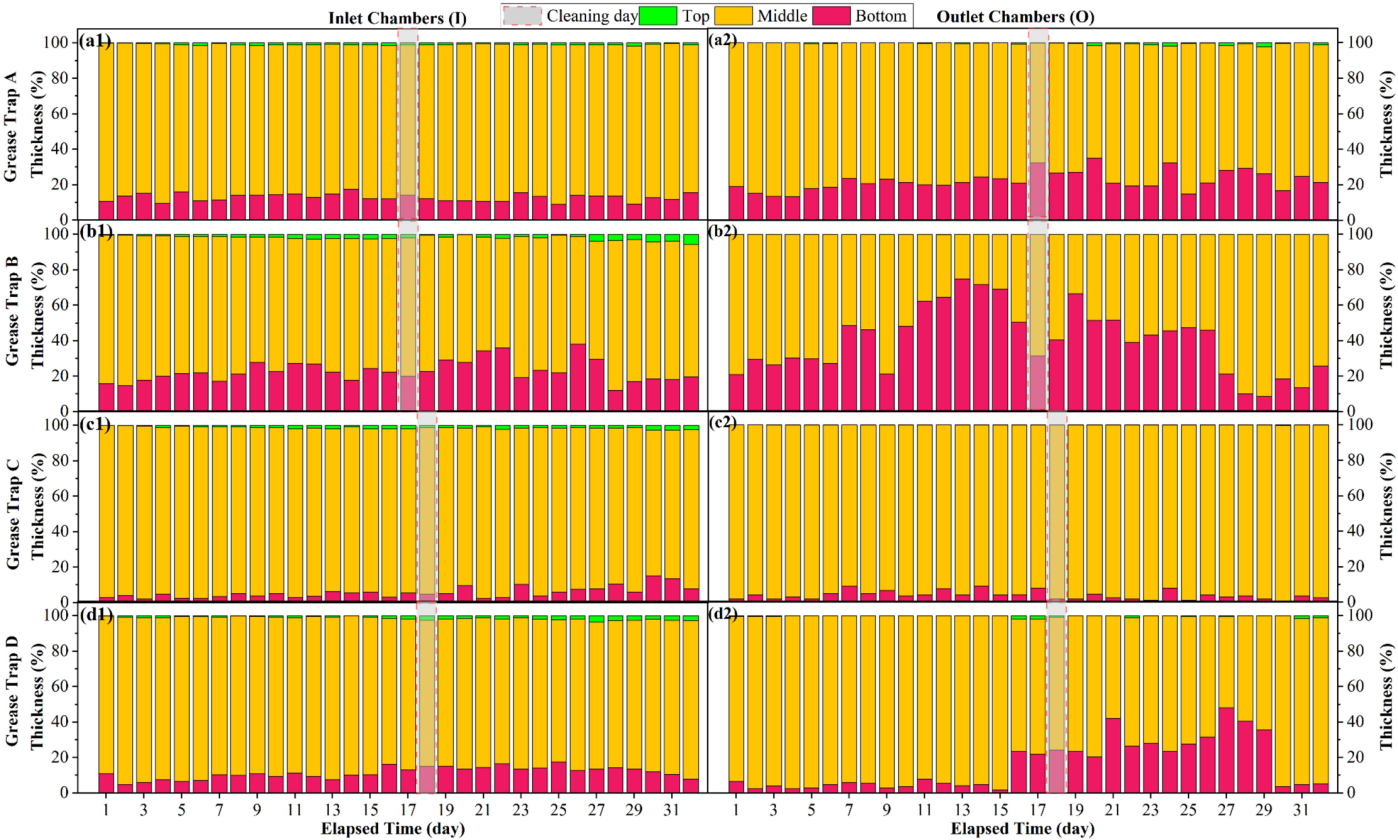
Normalized three‐layer thickness profiles (%) of Grease Traps A–D over a 32‐day site investigation. Subplots (a1‐d1) represent the inlet chambers (I), while subplots (a2‐d2) represent the outlet chambers (O). Vertical makers on Day 17 (Grease Traps A and B) and Day 18 (Grease Traps C and D) indicate desludging and cleaning events.

Figure 2 presents the normalized three‐layer thickness profiles (%) for the four grease traps over the 32‐day SI, highlighting the impacts of the desludging/cleaning events on Days 17 and 18. Detailed measurements (mm) and percentage compositions for the top (T), middle (M), and bottom (B) layers are summarized in Tables S1 and S2. Across all units, the total liquid depth averaged 851 ± 9.4 mm. As shown in Table 4, the composition for the inlet (I) and outlet (O) chambers was: T (1.41 ± 0.22% /0.29 ± 0.11%), M (85.4 ± 3.7% /79.3 ± 7.6%), and B (13.2 ± 3.6% /20.4 ± 7.6%).

**TABLE 4:** Average thickness (mm) and normalized profile composition (%) of the stratified layers for inlet and outlet chambers across all studied grease traps.

Chamber	Layer thickness (mm)				Composition (%)		
	Total	Top	Middle	Bottom	Top	Middle	Bottom
Inlet (I)	851 ± 9.4	11.9 ± 1.9	726 ± 25	114 ± 32	1.41 ± 0.22	85.4 ± 3.7	13.2 ± 3.6
Outlet (O)	851 ± 9.4	2.45 ± 0.9	673 ± 59	176 ± 67	0.29 ± 0.11	79.3 ± 7.6	20.4 ± 7.6

The top‐floated scum layer at the inlet chambers was approximately 4.86 times thicker than at the outlet chambers (11.9 vs. 2.45 mm). This disparity suggests that the baffle wall effectively retains the buoyant scum layer within the primary chamber (Figures S3 and S5). Conversely, the middle liquid and bottom sludge layers exhibited minor inter‐chamber differences of only 7.2% (85.4%–79.3%) and 6.1% (20.4%–13.2%), respectively. This indicates that the baffle wall opening fails to effectively isolate the M and B layers. This was particularly evident in Grease Traps A and B (serving 20 and 16 stalls, respectively), which were subjected to higher hydraulic loading and shorter HRTs compared to Grease Traps C and D (Table S3).

This performance gap may be ascribed to the baffle wall opening height (~225 mm from the base), which is positioned too low relative to the sludge interface (Figure S3). This configuration allows both settled and suspended solids to migrate from the inlet to the outlet chamber, leading to the rapid, excessive sludge accumulation that necessitated the desludging observed on Days 17 and 18 (Figure 2). These results imply that bottom sludge thickness is a more critical parameter than top scum accumulation when optimizing baffle opening height to prevent solids bypass. This finding aligns with the observations of Aziz et al. (2012) and Ducoste et al. (2008).

### 3.2 Floating and Settling Behaviors

**FIGURE 3:**
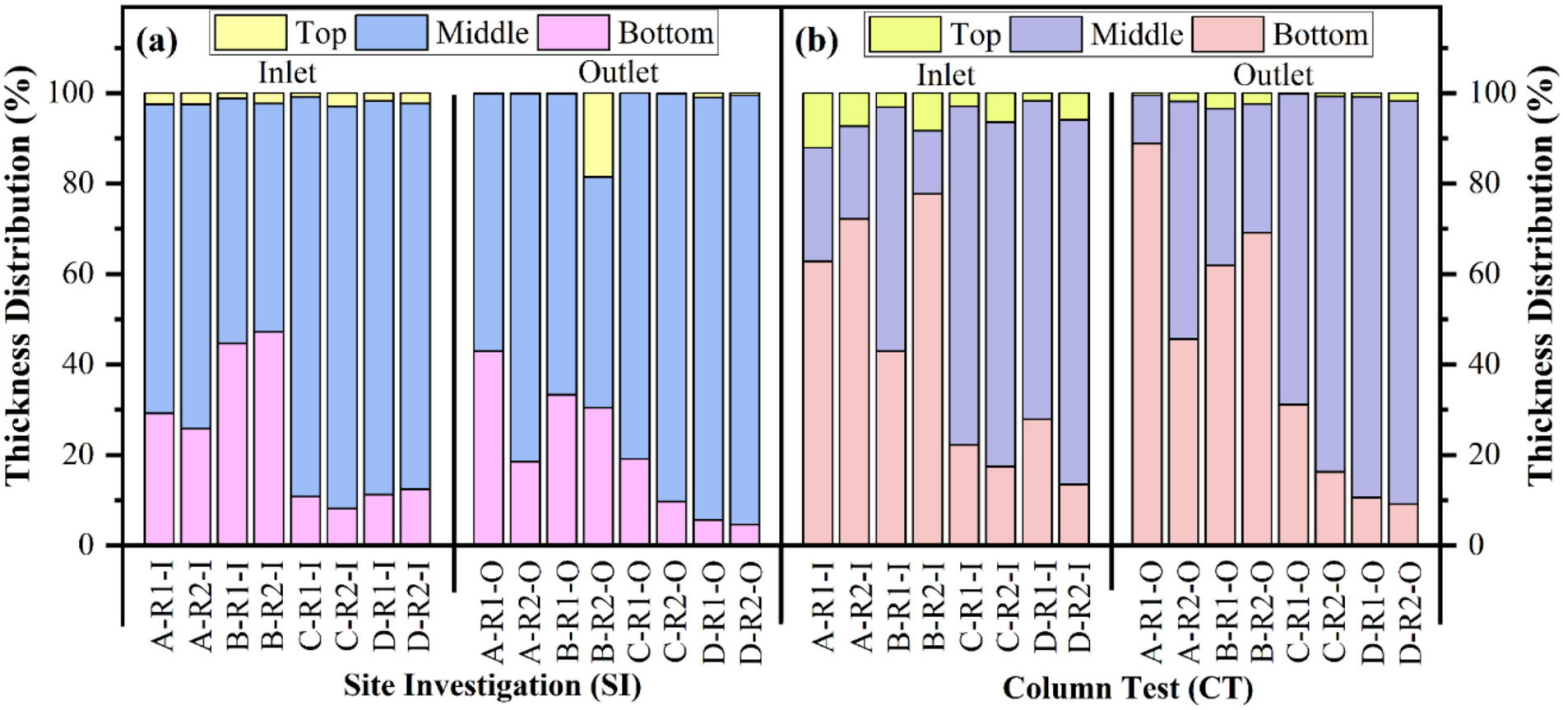
Comparative analysis of (a) site investigation (SI) and (b) laboratory column test (CT) results, showing the average percentage thickness distribution of the top, middle, and bottom layers for Grease Traps A–D. Data represent the mean values for inlet and outlet chamber samples obtained across two sampling rounds (R1 and R2).

Figure 3 illustrates the comparative three‐layer thickness profiles between the SI and CT results. While SI observations were limited to a < 5‐min window, the CTs were conducted under controlled laboratory conditions for > 60 min, providing a more comprehensive understanding and accumulation kinetics. Compared with the SI results, the extended CT duration led to a 2.91 times greater scum thickness in the inlet chamber, whereas the outlet chamber showed a 1.75 times smaller. Similarly, the settled sludge layer in the CTs was 1.81 and 2.07 times thicker in the inlet and outlet chambers, respectively. These results demonstrate that an extended HRT significantly enhances phase separation, confirming that a longer HRT is essential for stabilizing floating‐settling and separation‐accumulation processes.

**FIGURE 4:**
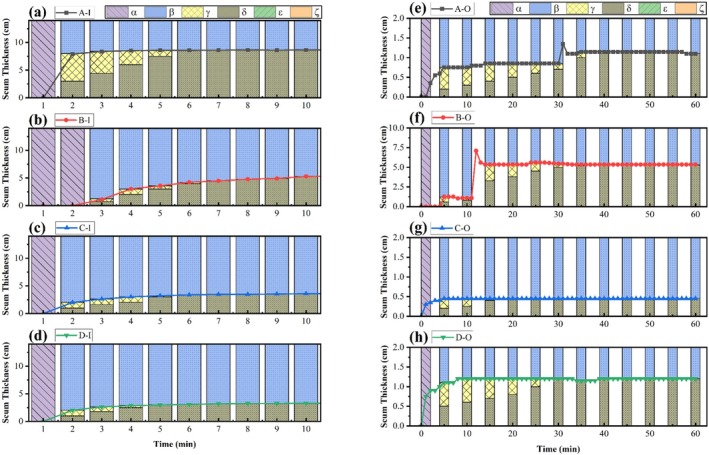
Floating kinetics of scum layers in the inlet (I) and outlet (O) chambers for Grease Traps (a, e) A, (b, f) B, (c, g) C, and (d, h) D. Data for inlet chambers represent the mean values of the initial and replicate column tests (C1 and C2).

**FIGURE 5:**
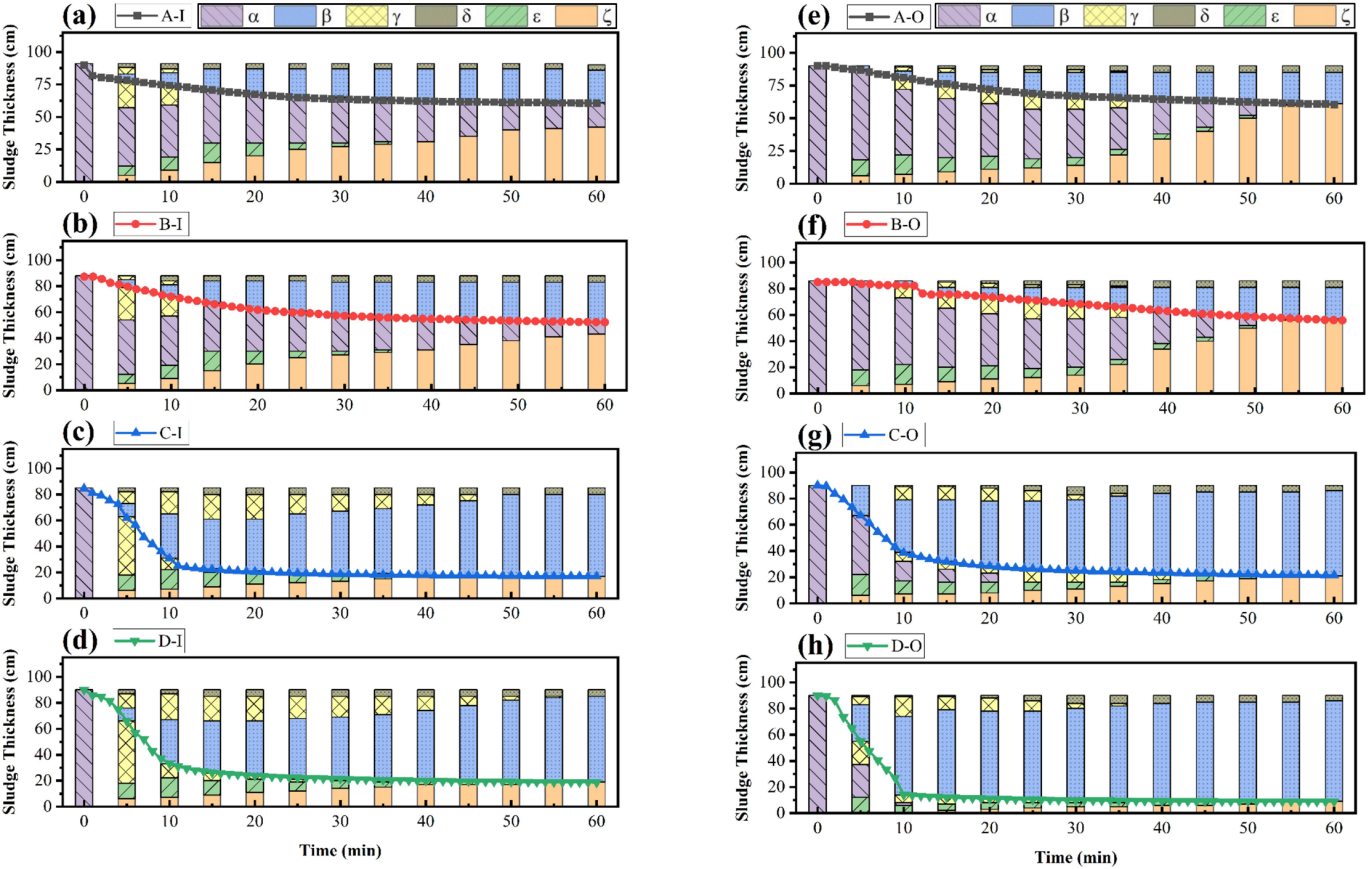
Settling kinetics of the settled sludge layers in the inlet (I) and outlet (O) chambers for Grease Traps (a, e) A, (b, f) B, (c, g) C, and (d, h) D. Inlet chamber data represent the mean values of C1 and C2 column test replicates.

Furthermore, the CT results reveal that grease‐trap wastewater transitions through six distinct zones (Zone α to Zone ζ) during the formation of the three primary layers (Figures 4 and 5):

- Zone β (**Clarified Zone**): The transparent liquid region situated between the floating scum and settling sludge interfaces (**Interfaces II and III**, Figure 1).
- Zone γ (**Transition Layer**): The regions where scum (Top Zone γ) and sludge (Bottom Zone γ) begin to consolidate, acting as the boundary for the distinct top and bottom layers.
- Zone α (**Clustered Zone**): Characterized by the aggregation of solids, forming an intermediate layer between Zones γ and ε.
- Zone ε (**Settling Layer**): The region between **Interface III** and the base, where solids descend and begin to compress under their own mass.
- Zone ζ (**Compression Zone**): The final, denser sludge layer formed by the compaction of settled solids at the bottom.
- Zone δ (**Scum Zone**): The region between **Interfaces I** and **II** where floating FOG accumulates to establish the uppermost solid–liquid interface.

This systematic classification enhances the understanding of grease‐trap separation dynamics by highlighting the progressive transition from a homogenized mixture to stratified layers through distinct floating and settling mechanisms.

Consequently, Grease Traps A and B exhibited higher solid accumulation in both the scum (Zone δ) and sludge (Zone ζ) layers compared to Grease Traps C and D (Figures 4 and 5), corroborating the daily SI observations (Figure 2). In contrast to the field results, the laboratory CT tests revealed that the floated scum thickness (Zone δ) generally increased over time before reaching a stable plateau (Figure 4). Notable exceptions occurred in the A‐O (Figure 4e) and B‐O (Figure 4f) samples, where scum thickness peaked sharply before declining. For example, the A‐R1‐O sample (black‐square marker line) reached a maximum at 30 min. The subsequent decline was likely attributable to extensive sludge sedimentation (Zone ζ), which displaced the clarified liquid zone (Zone β), thereby hindering the final separation and prolonging the accumulation of the scum layer.

Conversely, the thickness of the settling sludge layers (Bottom Zone γ, α, ε, and ζ) across all samples gradually decreased until reaching stagnant plateaus (Figures 5 and S6a,c). Grease Trap C and D achieved stability rapidly (< 10 min), whereas Grease Traps A and B required significantly longer durations (> 20 min). This disparity is attributed to the greater layer thickness distributions and higher TSS concentrations in Grease Traps A and B (Figure 3; Tables 5 and S4). These results suggest that higher solid concentrations in grease‐trap wastewater prolong the settling process, a finding that aligns with van Loosdrecht et al. (2016) regarding hindered settling in municipal wastewater. To further investigate this, the relationship between settling duration and total solid mass (the product of concentration and volume: TS, kg m^−3^ × solid volume, m^3^) was subsequently explored (Table 6).

**TABLE 5:** Physicochemical characterization of the top, middle, and bottom layer for Grease Traps A–D, categorized by (a) inlet (I) and (b) outlet (O) chambers for sampling rounds R1 and R2. All the parameters are reported in kg m^−3^ unless otherwise specified for VS/TS, VSS/TSS, pH, temperature (°C), and electrical conductivity (EC, μS cm^−1^), and (c) the removal efficiency (%) of the inlet and outlet samples.

			Grease Trap A		Grease Trap B		Grease Trap C		Grease Trap D	
	Sample		A‐R1‐I	A‐R2‐I	B‐R1‐I	B‐R2‐I	C‐R1‐I	C‐R2‐I	D‐R1‐I	D‐R2‐I
(a)	Top	TS	155.3 ± 0.85	206.7 ± 4.05	141.7 ± 18.2	122.9 ± 3.67	146.1 ± 1.60	120.6 ± 3.32	126.2 ± 4.68	113.2 ± 7.23
		VS	144.5 ± 2.60	174.5 ± 4.49	131.2 ± 14.1	106.6 ± 6.23	109.5 ± 1.17	108.5 ± 0.60	115.8 ± 3.46	100.8 ± 4.68
		VS/TS	0.93	0.845	0.926	0.867	0.75	0.9	0.918	0.891
		FOG	341	449.3	135.5	112.5	81.21	137.1	102.3	226.7
	Middle	pH	4.2	4.42	4.79	4.95	4.39	4.78	5.11	4.84
		Temp	28	26.5	28	29.5	28	29.5	28	28
		TS	1.782 ± 0.04	1.258 ± 0.07	0.527 ± 0.01	0.518 ± 0.03	1.009 ± 0.02	0.544 ± 0.03	0.689 ± 0.05	0.580 ± 0.02
		VS	1.513 ± 0.04	1.033 ± 0.01	0.407 ± 0.02	0.473 ± 0.02	0.802 ± 0.03	0.422 ± 0.01	0.600 ± 0.05	0.504 ± 0.04
		VS/TS	0.849	0.822	0.772	0.914	0.795	0.776	0.871	0.87
		TSS	0.918 ± 0.02	0.407 ± 0.02	0.220 ± 0.01	0.243 ± 0.01	0.537 ± 0.02	0.158 ± 0.01	0.189 ± 0.01	0.142 ± 0.00
		VSS	0.887 ± 0.02	0.388 ± 0.01	0.218 ± 0.01	0.227 ± 0.01	0.513 ± 0.02	0.119 ± 0.00	0.182 ± 0.01	0.134 ± 0.00
		VSS/TSS	0.996	0.954	0.99	0.932	0.957	0.754	0.965	0.945
		TDS	0.824 ± 0.02	0.742 ± 0.03	0.222 ± 0.03	0.229 ± 0.03	0.504 ± 0.01	0.453 ± 0.00	0.384 ± 0.02	0.424 ± 0.01
		EC	350.7 ± 8.60	331.0 ± 5.60	120.0 ± 0.60	176.8 ± 2.00	394.7 ± 2.90	335.3 ± 2.00	330.3 ± 2.70	324.3 ± 0.70
	Bottom	TS	240.3 ± 10.8	248.8 ± 2.19	120.2 ± 0.59	129.8 ± 1.56	143.8 ± 6.15	217.4 ± 13.2	264.3 ± 12.9	219.4 ± 4.82
		VS	235.1 ± 10.2	220.2 ± 3.03	116.2 ± 0.51	122.7 ± 1.45	135.3 ± 5.27	180.4 ± 5.59	221.8 ± 15.7	205.6 ± 3.12
		VS/TS	0.978	0.885	0.966	0.946	0.941	0.83	0.839	0.937

			Grease Trap A		Grease Trap B		Grease Trap C		Grease Trap D	
	Sample		A‐R1‐O	A‐R2‐O	B‐R1‐O	B‐R2‐O	C‐R1‐O	C‐R2‐O	D‐R1‐O	D‐R2‐O
(b)	Top	TS	—	—	128.3 ± 6.30	—	—	—	96.40 ± 2.98	118.0 ± 2.40
		VS	—	—	104.3 ± 2.97	—	—	—	95.26 ± 3.23	112.4 ± 3.46
		VS/TS	—	—	0.813	—	—	—	0.988	0.953
		FOG	—	—	1.416	—	—	—	0	208
	Middle	pH	4.81	4.32	4.8	5.06	4.92	4.94	5.1	5.05
		Temp	27	26.5	28	29.5	28	29.5	28.5	28
		TS	1.513 ± 0.02	1.240 ± 0.03	0.651 ± 0.02	0.467 ± 0.03	1.373 ± 0.02	0.544 ± 0.02	0.418 ± 0.03	0.376 ± 0.02
		VS	1.278 ± 0.02	1.098 ± 0.02	0.569 ± 0.03	0.393 ± 0.01	1.060 ± 0.02	0.367 ± 0.01	0.338 ± 0.02	0.329 ± 0.01
		VS/TS	0.844	0.885	0.874	0.843	0.772	0.673	0.809	0.876
		TSS	0.736 ± 0.01	0.535 ± 0.01	0.293 ± 0.01	0.146 ± 0.00	0.809 ± 0.01	0.138 ± 0.00	0.126 ± 0.00	0.117 ± 0.00
		VSS	0.686 ± 0.00	0.526 ± 0.01	0.287 ± 0.00	0.143 ± 0.00	0.744 ± 0.01	0.119 ± 0.00	0.121 ± 0.00	0.114 ± 0.00
		VSS/TSS	0.932	0.984	0.977	0.985	0.92	0.863	0.965	0.981
		TDS	0.720 ± 0.02	0.671 ± 0.01	0.387 ± 0.02	0.282 ± 0.05	0.607 ± 0.01	0.467 ± 0.02	0.300 ± 0.02	0.260 ± 0.01
		EC	179.2 ± 0.50	443.7 ± 1.50	128.6 ± 0.60	140.0 ± 1.80	359.7 ± 0.30	325.3 ± 1.30	284.3 ± 0.90	182.4 ± 0.40
	Bottom	TS	224.3 ± 6.97	226.0 ± 5.38	202.6 ± 2.71	266.0 ± 12.6	110.7 ± 5.54	202.2 ± 11.6	202.6 ± 2.71	148.8 ± 4.46
		VS	183.8 ± 30.3	208.4 ± 4.85	190.7 ± 2.80	164.7 ± 7.67	105.3 ± 5.17	187.4 ± 10.1	190.7 ± 2.80	141.5 ± 4.21
		VS/TS	0.819	0.922	0.941	0.619	0.951	0.927	0.941	0.951

			Grease Trap A		Grease Trap B		Grease Trap C		Grease Trap D	
	Sample		A‐R1	A‐R2	B‐R1	B‐R2	C‐R1	C‐R2	D‐R1	D‐R2
(c)	Top	TS	100.0	100.0	9.5	100.0	100.0	100.0	23.6	−4.2
		FOG	100.0	100.0	99.0	100.0	100.0	100.0	100.0	8.2
	Middle	TS	15.1	1.4	−23.5	9.8	−36.1	0.0	39.3	35.2
		TSS	19.8	−31.4	−33.2	39.9	−50.7	12.7	33.3	17.6
		TDS	12.6	9.6	−74.3	−23.1	−20.4	−3.1	21.9	38.7
	Bottom	TS	6.7	9.2	−68.6	−104.9	23.0	7.0	23.3	32.2

*Note:* Negative values in (c) indicate that the parameter concentration or mass in the outlet chamber exceeded that measured in the inlet chamber, suggesting continued accumulation, resuspension, or redistribution within the system.

**TABLE 6:** Inventory of solid mass and layer concentrations for the inlet chambers of Grease Traps A–D after 60 min of separation. *Note:* Solid mass is calculated as the product of chamber surface area, layer thickness, and measured concentration.

Grease trap		A	B	C	D
Surface area (m^2^)		0.002	0.002	0.002	0.002
Layer thickness @1 h HRT (m)	Top	0.087	0.049	0.039	0.035
	Middle	0.205	0.302	0.638	0.679
	Bottom	0.606	0.522	0.169	0.186
Top scum (kg m^−3^)	Total solids	181	132	133	120
	Volatile solids	160	119	109	108
Middle clarified liquid (kg m^−3^)	Total suspended solids	0.663	0.232	0.348	0.166
	Volatile suspended solids	0.638	0.223	0.316	0.158
	Total dissolved solids	0.783	0.226	0.479	0.404
Bottom sludge (kg m^−3^)	Total solids	245	125	181	242
	Volatile solids	228	119	158	214
Top scum (kg)	Total solids	0.030	0.012	0.010	0.008
	Volatile solids	0.026	0.011	0.008	0.007
Middle clarified liquid (g)	Total suspended solids	0.256	0.132	0.418	0.212
	Volatile suspended solids	0.246	0.127	0.380	0.202
	Total dissolved solids	0.302	0.128	0.576	0.517
Bottom sludge (kg)	Total solids	0.280	0.123	0.058	0.085
	Volatile solids	0.260	0.118	0.050	0.075
Scum + Middle clarified liquid + Sludge (kg)	Total solids	0.310	0.136	0.068	0.094
	Volatile solids	0.287	0.129	0.059	0.082
**Remarks**		**High solid**	**High solid**	**Low solid**	**Low solid**

For instance, Grease Traps A and B, which contained higher total solids (TS) masses (0.123 to 0.280 kg) and sludge concentrations of 125 to 245 kg m^−3^, required longer settling durations than Grease Traps C and D, which had lower solid masses (0.058 to 0.085 kg) despite maintaining similar concentration ranges (181 to 242 kg m^−3^) (Table 6). Notably, while the TS concentrations in Grease Traps A and D were numerically comparable (245 vs. 242 kg m^−3^), their settling kinetics differed significantly, particularly regarding the time elapsed to reach the stagnant phase (Figure 5).

This observation suggests that solid concentration alone cannot fully explain the settling behavior observed within grease traps. Although both systems exhibited comparable sludge concentrations, Grease Trap A had a substantially larger total sludge mass and a thicker sludge layer, resulting in prolonged settling times. The larger sludge inventories increased particle‐particle interactions and hindered settling effects, thereby reducing effective settling velocities and extending the time required to achieve stable layer stratification. Collectively, these findings indicate that solids loading exerts a greater influence on settling behavior than concentration alone.

In contrast, Grease Traps A and B exhibited similar settling profiles, suggesting that a high TS mass (> 0.1 kg) is a more dominant determinant of settling duration than concentration alone. This phenomenon may be attributed to increased particle interactions in high‐mass sludge, which prolongs the transition to the compression stage. Effectively, the higher solid content in Grease Traps A and B delayed the attainment of stagnant plateaus compared to the lower solid content in Grease Traps C and D, indicating that a longer HRT is essential for achieving optimal three‐layer separation and accumulation. Consequently, the following sections analyze the floating and settling velocities of these layers to elucidate the specific physical mechanisms governing layer formation and phase separation.

Table 5 summarizes the pollutant retention efficiencies between the inlet and outlet chambers of the grease traps. Overall, FOG exhibited the highest removal performance, with most systems achieving near‐complete retention (> 99%), indicating that floatable grease fractions were effectively captured in the scum layer. In contrast, the removal efficiencies of TSS and bottom‐layer TS were highly variable and frequently negative. These observations suggest that, despite the formation of distinct scum and sludge layers, suspended and settleable solids were not consistently retained within the inlet chamber. Negative removal efficiencies indicate continued solids transport and accumulation in the outlet chamber, consistent with the substantial sludge accumulation observed during the site investigation.

The contrasting behavior between FOG and solids removal highlights the differing mechanisms governing grease‐trap performance. While buoyant FOG fractions were readily separated through floating and scum accumulation processes, the retention of suspended and settleable solids remained strongly influenced by solids loading and hydraulic conditions. Consequently, effective layer formation alone does not necessarily translate into satisfactory solids removal performance.

### 3.3 Floating and settling mechanisms

**FIGURE 6:**
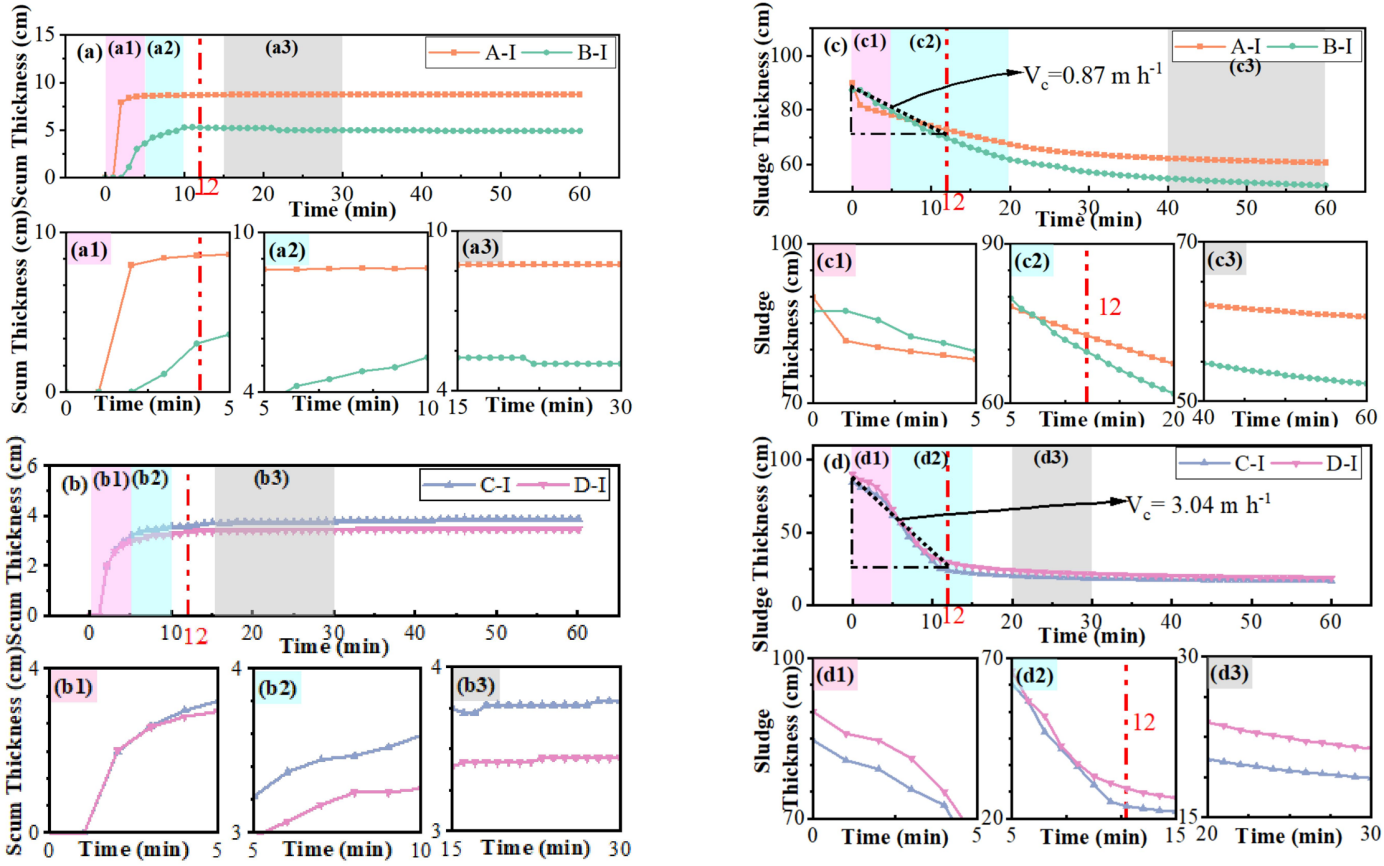
Scum flotation kinetic and sludge sedimentation profile of (a, c) high‐solid content wastewater (Grease Traps A and B) and (b, d) low‐solid content wastewater (Grease Traps C and D). Insets illustrate temporal dynamics of flotation and settling across selected intervals. The critical settling velocity (V_c_) at a 12‐min HRT was (c) 0.87 m h^−1^ and (d) 3.04 m h^−1^ for high‐ and low‐solid‐content wastewater, respectively.

Figure 6 highlights the distinct flotation trends observed in the inlet chambers of Grease Traps A–D during two critical intervals: *t*_0_ to *t*_5_ (**Inset** Figure 6a1) and *t*_5_ to *t*_10_ (**Inset** Figure 6a2). During the initial phase (*t*_0_ to *t*_1_, Figure S7a), the profiles exhibit their steepest gradients, corresponding to the peak flotation velocity. This behavior coincides with the discrete flotation mechanism (**Type F.I**). Between *t*_4_ and *t*_6_, the flotation velocity decreases, and the profiles stabilize (Figure S7), marking the transition to a compression flotation mechanism (**Type F.II**), though only minor continuous compression of the scum layer was observed thereafter.

The steepest gradient measured during the **Type F.I** phase was utilized to determine the critical flotation velocity (Figure S7a). As expected, the inlet chamber samples exhibited a significantly higher average flotation velocity (1.79 m h^−1^) compared to the outlet samples (0.90 m h^−1^), likely due to the higher FOG content in the inlet. This is supported by previous studies (Stewart and Arnold 2011; Sakeena et al. 2011), which indicate that higher FOG concentrations promote larger droplet sizes and accelerated buoyancy. This relationship explains why the flotation velocity in Grease Trap A surpassed those of Grease Traps B, C, and D (Figures S8 and S9). Consequently, higher FOG concentrations in buoyant particles increase flotation velocity, leading to rapid layer formation before the system reaches its optimal HRT.

Figure S7b illustrates two distinct sedimentation patterns observed in the column test (CT) for high‐ and low‐solid grease‐trap wastewater. During the initial phase (*t*_0_ to *t*_1_; **Inset** Figure 6c1), high‐solid wastewater exhibited a steep decline governed by discrete and flocculant settling mechanisms (**Types S.I** and **S.II**), resulting in a high initial settling velocity (Figure 6c). Between *t*_5_ and *t*_20_ (**Inset** Figure 6c2), the gradient became more gradual, signifying a transition to hindered settling (**Type S.III**). Beyond 40 min (**Inset** Figure 6c3), the profile reached a stagnant plateau, indicating the onset of the compression settling mechanism (**Type S.IV**) and a corresponding reduction in velocity. In contrast, the low‐solid wastewater initially settled more slowly (*t*_0_ to *t*_4_; **Inset** Figure 6d1), likely due to initial mixing effects and a brief period of **Type S.III** behavior. However, the settling rate accelerated between *t*_5_ and *t*_20_ (Figure 6d), suggesting a shift toward **Type S.I** and **S.II** mechanisms, which increased the settling velocity. Lastly, after 20 min, **Type S.IV** was initiated, resulting in a further decrease in settling velocity as the sludge layer stabilized.

Consequently, the hindered settling mechanism (**Type S.III**) in Grease Traps A and B (high‐solid wastewater) resulted in lower settling velocities compared to Grease Traps C and D, thereby prolonging the time required to reach a stagnant plateau (Figure 6c,d; Tables S5–S8). In contrast, the low‐solid wastewater in Grease Traps C and D promoted rapid discrete settling (**Types S.I** and **S.II**) with negligible interference from hindered settling (**Type S.III**). This resulted in the inlet chamber samples, which contain higher solid loads, exhibiting a slightly higher average settling velocity (2.59 m h^−1^) than the outlet chamber samples (2.28 m h^−1^) (Figure S7b).

The data suggest that elevated solid concentration enhances **Type S.III** behavior, which extends the total sedimentation duration. By the 50‐min mark, settling velocities showed minimal variation, indicating that the wastewater had transitioned into the compression settling mechanism (**Type S.IV**). Notably, high‐solid wastewater (Grease Traps A and B) stabilized faster (~20 min) than low‐solid wastewater (~45 min) (Figure S9a,c). These findings imply that low‐solid environments can accelerate the settling velocity once denser particles aggregate. In short, because the flotation and sedimentation mechanisms stabilize within 30 to 50 min, an optimal HRT of 0.5 to 1 h is recommended. This duration ensures sufficient physical separation and accumulation of FOG, FOG deposits, and other residues, particularly within the primary inlet chambers.

Conversely, the CT results demonstrated that, while most samples from Grease Traps A–D stratified into three distinct layers, specific samples, such as A‐R1‐I‐C1, B‐R2‐I‐C1, and B‐R2‐I‐C2, exhibited more complex phase behavior (Figures 4 and 5). For instance, the A‐R1‐I‐C1 sample initially formed five discrete layers (< 5 min) before consolidating into three as the middle‐layer solids settled after 20 min (Figure S10; Tables S9–S16). In contrast, the B‐R2‐I‐C1 and B‐R2‐I‐C2 samples maintained five layers for over 1 h (Table S10), suggesting that 1 h was sufficient for complete phase separation in these cases. These variations are likely ascribed to the diverse wastewater composition discharged by different F&B stalls, which significantly alter the physicochemical properties and settling mechanisms of the influent. Consequently, such high‐load conditions necessitate a longer HRT to achieve stable three‐layer accumulation.

Despite these irregularities, the settling velocity (*V*_s_), rather than the floating velocity (*V*_*f*_), was identified as the critical velocity (*V*_*c*_) at an HRT of 12 min (0.20 h). Specifically, *V*_*c*_ values of 0.87 and 3.04 m h^−1^ were determined for high‐solid and low‐solid wastewater, respectively (Figure 6c,d). These critical velocities were subsequently used to calculate SOR and the required surface area (*A*_*s*_) for the investigated grease traps. These parameters provide a basis for evaluating their performance against commercial grease‐trap specifications and established design standards.

### 3.4 SOR and Hydraulic Retention Time (HRT)

Figure S11 presents a schematic diagram illustrating the sedimentation of suspended particles between the inlet and outlet chambers, highlighting the derivation of two critical design parameters: SOR and HRT. Table 7 summarizes the evaluation of these parameters for high‐solid (Grease Traps A and B) and low‐solid (Grease Traps C and D) conditions, identifying the optimal values for effective separation.

**TABLE 7:** Evaluation of design parameters for high‐solid (A and B) and low‐solid (C and D) grease traps to determine optimal HRT and SOR.

*Cell*	Design parameters and calculations	High‐solid	Low‐solid	Remarks (if any)
*A1*	Actual influent flow rate, Q (m^3^ d^−1^)	25.7	17.9	*Average Q of Grease Traps A&B and B&C from* Table S3
*A2*	Actual surface area, *A*_*s*,act_, (m^2^)	0.55	0.55	(WEIDA Integrated Industries Sdn Bhd 2021)
*A3*	Find actual SOR_act_ (m^3^ d^−1^ m^−2^)	46.7	32.5	*= Cell A1/A2; SOR*_*act*_ *= Q/A*_*s,act*_ *> > SOR*_*crit*_
*A4*	Critical time, t_crit_ (h)	0.20	0.20	*Critical HRT@12 min from* Figure S10
*A5*	Critical velocity, V_c_ (m h^−1^)	0.87	3.04	*Critical settling velocity from* Figure S10
*A6*	Find critical surface overflow rate, SOR_crit_ (m^3^ d^−1^ m^−2^)	20.9	73.0	= Cell A5*24 when Vs *= Vc; SOR*_*crit*_ *< < SOR*_*act*_
*A7*	Find required surface area, *A*_*s*,req_, (m^2^)	1.23	0.25	*= Cell A1/A6; NG (> A*_*s,act*_ *) for high‐solid; OK (< As,act) for low‐solid*
*A8*	Average thickness, H (m)	0.86	0.84	*Average total liquid thickness of Grease Traps A and B and C and D from* Tables S1 and S2
*A9*	Find settling time, t_s_ (h)	0.99	0.28	= Cell A8/A6*24; ts = H/SOR_*crit*_
*A10*	Measured total grease‐trap diameter, Ø_t_ (m)	0.99	0.99	*994 mm measured on‐site*; Figure S3
*A11*	Measured inlet‐chamber diameter, Ø_i_ (m)	0.27	0.27	*270 mm measured on‐site*; Figure S3
*A12*	Measured outlet‐chamber diameter, Ø_o_ (m)	0.34	0.34	*340 mm measured on‐site*; Figure S3
*A13*	Estimated inlet‐chamber length, L (m)	2.05	2.05	= Cell [(2*π*(A10/2) − (2*π*(A12/2)]
*A14*	Estimated inlet‐chamber width, W (m)	0.27	0.27	*= Cell A11; Same as the diameter of the inlet chamber*
*A15*	Estimated horizontal velocity, V_h_ (m h^−1^)	4.61	3.28	*= Cell A1/A8/A14/24; V*_*h*_ = Q/(H*W)
*A16*	Estimated horizontal time, t_h_ (h)	0.45	0.63	*= Cell A13/A15; t*_*h*_ *= L/V*_*h*_ *; OK (< t*_*s*_ *) for high‐solid; NG (> t*_*s*_ *) for low‐solid*
*A17*	Find favorable HRT (h)	0.99	0.63	*= max(A4,A9,A16)*
*A18*	Use HRT	1.00	0.70	*= roundup(A17,1); When t*_*s*_ *= t*_*h*_ *, favorable final HRT = 0.70 to 1.00 h*
*A19*	Final H (m)	0.86	0.86	*= max(A8)*
*A20*	Final L (m)	2.05	2.05	*= max(A13)*
*A21*	Find Vs, (m/h)	0.86	1.23	*= Cell A19/A18*; V_s_ *= H/ts*
*A22*	Find Vh, (m/h)	2.05	2.94	*= Cell A20/A18; V*_*h*_ *= L/ts*
*A23*	Find W, (m)	0.61	0.30	= Cell A1/24/A8/A20; W = Q/(H*Vh)
*A24*	Final W, (m)	0.61	0.61	*= max(A23)*
*A25*	Final A_s_ (m^2^)	1.25	1.25	= Cell A13*A21; A_*s*_ = L*W
*A26*	Find SOR (m^3^ d^−1^ m^−2^)	20.6	14.4	*= Cell A1/A22; SOR = Q/A*_*s*_
*A27*	Final SOR (m^3^ d^−1^ m^−2^)	20.6	20.6	*= max(A26); Check against SOR*_*crit*_ *OK!*
*A28*	Find final volume (m^3^)	1.07	1.07	= Cell A19*A20*A24; Volume = H*L*W
*A29*	Check final HRT (h)	1.00	1.44	= Cell A28/A1*24; HRT = V/Q; Check OK!
*A30*	Find the required grease trap diameter, Ø_req_ (m)	1.30	1.30	= 2*(SQRT((Cell A25 + (PI()*(Cell A12/2)^2))/(PI())))

The analysis was initiated using the critical settling velocities (*V*_*c*_ = V_s_) of 0.87 and 3.04 m h^−1^ for high‐solid and low‐solid wastewater, respectively, derived at a 12‐min (0.20 h) HRT (Figure 6c,d). Consequently, the critical SORs (SOR_crit_) were determined to be 20.9 and 73.0 m^3^ d^−1^ m^−2^. Based on the actual influent flow rates (*Q*) of 25.7 and 17.9 m^3^ d^−1^ (Table S3), the required *A*_*s*_ were calculated as 1.23 m^2^ for high‐solid conditions and 0.25 m^2^ for low‐solid conditions. These results reveal a significant design gap: the existing grease trap surface area (0.55 m^2^) is sufficient for low‐solid wastewater (0.25 m^2^ required) but is critically inadequate for high‐solid loads (1.23 m^2^ required). This disparity necessitates a re‐evaluation of current grease‐trap design criteria, as detailed in the following sections.

Consequently, the total liquid layer thicknesses for Grease Traps A and B (0.86 m) and C and D (0.84 m) were estimated from average liquid heights recorded over the 32‐day monitoring period. Using these values, the settling times (t_s_) and favorable HRTs were determined to be 0.99 h (0.28 h) and 0.99 h (0.63 h) for high‐solid (low‐solid) conditions, respectively. Based on the recommended HRTs (1.00 and 0.70 h), the final design dimensions, liquid height (*H*), length (*L*), and width (*W*), were established as 0.86, 2.05, and 0.61 m, respectively. Ultimately, the required *A*_*s*_ was determined to be 1.25 m^2^ with a corresponding SOR of 20.6 m^3^ d^−1^ m^−2^. These calculations utilized the high‐solid influent flow rate (*Q*) as the critical design condition. Notably, this SOR limit (20.6 m^3^ d^−1^ m^−2^) is significantly lower than the typical range for sedimentation tanks in a water resource recovery facility (30 to 70 m^3^ d^−1^ m^−2^) (Tchobanoglous et al. 2014). This discrepancy is likely due to the critical floating and settling velocities of grease‐trap wastewater, which are inherently lower than those of the biofloc generated during activated sludge processes.

Table 8 summarizes the estimated SOR and *A*_*s*_ limits based on the dimensions, working *V*s, HRTs, and flow rates (*Q*) of commercial grease traps from various regions, including those evaluated in this work (Weida Integrated Industries Sdn Bhd 2021). The results indicate that Grease Traps A–D fail to meet the required *A*_*s*_, SOR, and HRT thresholds. Compared to standards in Australia (Australian Building Codes Board 2019; Federation Council 2026) and the United States (Wong 1998; Wong et al. 2007), grease traps from Hong Kong, Indonesia, Italy, and Singapore were found to have insufficient *A*_*s*_ and/or HRT. This deficiency is likely due to the more generous surface area and retention time specifications in Australian and American designs, which facilitate superior three‐layer stratification (separation and accumulation). Nonetheless, it is crucial to account for regional variations in the physicochemical properties of grease‐trap wastewater when establishing these critical SOR and HRT limits, as influent characteristics directly influence separation efficiency.

**TABLE 8:** Comparative analysis of surface overflow rate (SOR) and surface area (*A*_*s*_) limits for commercial grease trap designs across various regions, including those investigated in this work.

Country	Size (mm)	Actual				Required limits			Remarks	Ref.
		Volume (L)	Q (m^3^ d^−1^)	HRT (h)	*A*_*s*_ (m^2^)	*A*_*s*_ ^a^ (m^2^)	SOR (m^3^ d^−1^ m^−2^)	HRT (h)		
Sarawak, Malaysia	Ø994	500	14–29	0.14–0.28	0.55	1.25	14.4–20.6	0.70–1.00	Insufficient HRT and ***A***_***s***_	**This work**
Australia	L1800 × W1000 × H900	> 1000	24	1.00	1.80	1.15	13.3	Min. 1.00	OK	(Australian Building Codes Board 2019; Federation Council 2026)
Hong Kong, China	L2000 × W2000 × H1800	> 250	18	0.33	8.00	0.87	2.27	Min. 1.00	Insufficient HRT	(Environmental Protection Department 2022)
Indonesia	L800 × W400 × H1000	256	2	3.10	0.32	0.09	6.19	Min. 1.00	OK	(Purwanti et al. 2018)
Italy	Ø1050	1100	106	0.25	0.87	5.05	122	Min. 1.00	Insufficient HRT and ***A***_***s***_	(Stampaggio Materie Plastiche 2022)
Singapore	Ø2060	> 1000	48	> 0.50	1.58	2.30	30.4	Min. 1.00	Insufficient HRT and ***A***_***s***_	(Singapore's National Water Agency 2019; British Standards Institution 2004)
United State	L3200 × W1524 × H1524	3785	14	6.68	4.88	0.65	2.79	Min. 1.00	OK	(Wong et al. 2007; Wong 1998)
United State	L4876 × W1219 × H1473	7570	11	17.3	5.94	0.50	1.77	Min. 1.00	OK	(Wong et al. 2007; Wong 1998)
United State	L4572 × W2438 × H1828	8706	10	20.6	11.2	0.49	0.91	Min. 1.00	OK	(Wong et al. 2007; Wong 1998)
United State	L3200 × W1524 × H1524	20,441	19	26.0	4.88	1.24	5.31	Min. 1.00	OK	(Wong et al. 2007; Wong 1998)

^a^ Required *A*_*s*_ is calculated based on the critical floating‐settling velocity (*V*_c_) of this work.

Table 9 presents the solid removal efficiencies for the three distinct layers between the inlet and outlet chambers under varying solid‐loading conditions. The results demonstrate that both high‐ and low‐solid wastewater effectively sequestered the top scum layer, achieving TS removal efficiencies of 77% and 55%, respectively. In contrast, low‐solid wastewater exhibited improved retention of bottom‐settled sludge, with only 21% of the settled solids reaching the outlet chamber. This suggests that the existing surface area (0.25 m^2^) was sufficient to retain a substantial proportion of settleable solids under low‐solid‐loading conditions (Table 7), although it remained inadequate to achieve satisfactory overall solids removal. However, there was no significant reduction in suspended solids in the middle‐clarified layer (3% TSS). This is further evidenced by the TSS concentrations across Grease Traps A–D (0.376 to 1.513 g L^−1^, Table 5), which fail to meet the Malaysian Sewerage Industry Guidelines limit of 100 mg L^−1^ (National Water Services Commission 2009).

**TABLE 9:** Estimated removal efficiencies for TS and TSS across the stratified top, middle, and bottom layers between the inlet and outlet chambers.

Three layers	Inlet chamber	Outlet chamber	Removal efficiency of high (low) solid content^a^
Top‐scum layer (TS, g L^−1^)	157 ± 10.2 (127 ± 4.19)	32.1 ± 16.8 (53.6 ± 16.3)	77% (55%)
Middle clarified liquid layer (TSS, g L^−1^)	0.45 ± 0.08 (0.26 ± 0.05)	0.43 ± 0.07 (0.30 ± 0.09)	−1% (3%)
Bottom‐sludge layer (TS, g L^−1^)	185 ± 18.2 (211 ± 13.7)	230 ± 7.65 (166 ± 12.1)	−39% (21%)

^a^ Removal efficiency is the average efficiency calculated based on the grease trap efficiency for each sample.

The observed removal efficiencies further highlight the importance of hydraulic conditions in governing grease‐trap performance. Although distinct scum, clarified liquid, and sludge layers were formed, the existing grease traps did not meet the proposed HRT, SOR, and surface area criteria, thereby limiting the hydraulic conditions required for effective floating, settling, and accumulation. Consequently, while buoyant FOG fractions were effectively retained within the scum layer, suspended and settleable solids were more susceptible to carrying over into the outlet chamber, resulting in poor, and occasionally negative, TSS removal efficiencies. These findings suggest that hydraulic conditions influence not only layer formation but also the overall pollutant retention performance of grease traps.

Consequently, it is estimated that a minimum *A*_*s*_ of 1.25 m^2^, a SOR of 20.6 m^3^ d^−1^ m^−2^, and a 1‐h HRT are required to optimize separation and accumulation (Table 7A25,A27,A29). To meet these criteria, the total diameter of Grease Traps A–D should be increased from 0.99 to 1.30 m, while maintaining the current outlet chamber dimension (Table 7A30).

### 3.5 Proposed physical separation and accumulation mechanisms

Following 24 CT performance on 16 grease‐trap wastewater samples, two flotation mechanisms (**Types F.I and F.II**) and four sedimentation mechanisms (**Types S.I, S.II, S.III, and S.IV**) were concurrently identified and characterized. In the initial stages, discrete and flocculant settling (**Types S.I and S.II)** and hindered settling **(Type S.III)** were found to be the predominant mechanisms for high‐solid‐content wastewater. Conversely, low‐solid‐content wastewater was primarily governed by discrete settling mechanisms (**Types S.I and S.II**), with minimal to no hindered settling observed (**Type S.III**).

**FIGURE 7:**
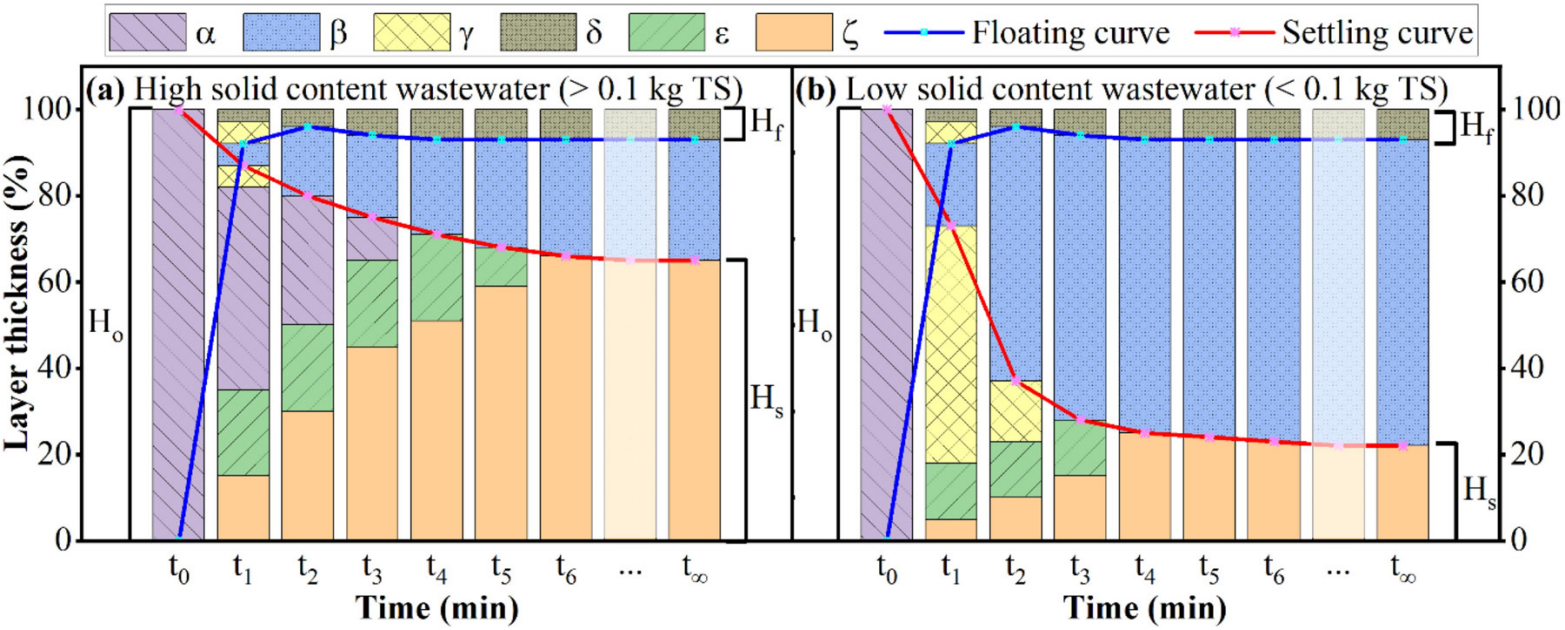
Schematic representation of the proposed physical separation and accumulation mechanisms for (a) high‐solid and (b) low‐solid grease‐trap wastewater, illustrating the development of three‐layer stratification.

Figure 7 presents schematic diagrams illustrating the physical separation and accumulation mechanisms within a grease trap. The diagrams emphasize the flotation and sedimentation behaviors that drive the stratification of high‐solid (> 0.1 kg TS, Figure 7a) and low‐solid (< 0.1 kg TS, Figure 7b) wastewater into three distinct layers. Initially (*t*_*0*_), the homogeneous wastewater occupies a total height *H*_*0*_ within a clean column. By *t₁*, high‐solid wastewater undergoes discrete flotation (Top Zone γ, **Type F.I**) while simultaneously experiencing discrete and flocculent sedimentation (Bottom Zone γ, **Types S.I and S.II**) and hindered settling (Zone α, **Type S.III**). This simultaneous migration of particles leads to the emergence of a clarified region (Zone β), resulting in two distinct solid–liquid interfaces at the top (Top Zones γ, δ) and bottom (Bottom Zones γ, α, ε, and ζ), effectively dividing the system into seven zones.

While discrete flotation (**Type F.I**) allows scum to accumulate at the surface, the process stabilizes rapidly (typically between *t*_*0*_ and *t*_*2*_). Subsequently, the layer enters compression flotation (Zone δ, **Type F.II**), which defines the final floated solid–liquid interface. In parallel, most of the sludge transitioning from a discrete settling (Bottom Zone γ, **Types S.I and S.II**) enters the hindered settling phase (Zone α, **Type S.III**), followed by a transition settling zone (Zone ε). This culminates in the formation of a dense sludge layer in Zone ζ via compression settling (**Type S.IV**). After *t₆*, both the surface scum and bottom sludge undergo final compression, establishing the terminal layer heights, *H*_*f*_ and *H*_*s*_.

Conversely, low‐solid‐content wastewater follows a similar flotation mechanism but exhibits distinct sedimentation behavior. While the initial homogeneous state (*t*_0_) transitions to form two solid–liquid interfaces and a clarified region (Zone β) by *t*₁, only six zones are observable. Notably, the hindered sedimentation zone (Zone α, **Type S.III**) is absent. This variation is attributed to the low solids concentration, which promotes discrete settling (Bottom Zone γ, **Types S.I and S.II**) over hindered settling (Zone α, **Type S.III**), which dominates at high solids concentrations. Consequently, the transition settling phases in Zone ε shift directly from discrete settling (Bottom Zone γ, **Types SI and S.II**) to compression settling (Zone ζ, Type **S.IV**), effectively bypassing the hindered settling stage (Zone α, **Type S.III**). As a result, the transition settling phase concludes significantly earlier in low‐solid wastewater compared to high‐solid wastewater (*t*_*4*_ vs. *t*_*6*_). This indicates that sludge accumulation occurs more rapidly under low‐solid conditions, as larger discrete particles settle faster with minimal inter‐particle interference. These findings are consistent with the critical velocities measured in the CTs (Table 7), where the critical setting velocity for low‐solid wastewater (3.04 m h^−1^) was significantly higher than that of high‐solid wastewater (0.87 m h^−1^).

## 4 Conclusion

This work characterized the mechanisms of physical separation and accumulation of FOG and solid residues in grease traps. Site investigations revealed that sludge accumulation at the bottom was up to 9.3 times greater than the surface scum layer. Grease trap wastewater was categorized by loading; high‐solid‐content (> 0.1 kg TS) samples required significantly longer phase‐separation times. While higher FOG concentrations improve flotation velocities (1.79 vs. 0.90 m h^−1^), the increased solid concentration inhibited sedimentation, resulting in lower settling velocities than in low‐solid samples (2.28 vs. 2.59 m h^−1^).

CTs identified a critical settling velocity of 0.87 m h^−1^ at 12 min, suggesting that a 1‐h HRT is a sufficient design benchmark. To ensure effective three‐layer stratification, grease traps require a minimum surface area (*A*_*s*_) of 1.25 m^2^ and an SOR of 20.9 m^3^ d^−1^ m^−2^. Most existing grease traps in Malaysia, including the units investigated here, fail to meet these thresholds.

Mechanistically, this work identifies two flotation (**Types F.I and F.II**) and four sedimentation (**Types S.I, S.II, S.III, and S.IV**) types. High‐solid wastewater undergoes discrete, flocculent, and hindered settling, whereas low‐solid wastewater bypasses the hindered phase. These findings provide a scientific basis for re‐evaluating grease trap design standards. Future research should focus on long‐term monitoring and complex physicochemical interactions under controlled laboratory conditions.

## Author Contributions

**Ling Ying Tang:** conceptualization, data curation, methodology, formal analysis, investigation, validation, writing – original draft. **Alex Kwong Jun Kiu:** data curation, formal analysis, investigation, writing – review and editing. **Ngie Hing Wong:** conceptualization, formal analysis, funding acquisition, project administration, resources, supervision, writing – review and editing. **Chung Siung Choo:** supervision, writing – review and editing. **Lily Li:** conceptualization, writing – review and editing. **Chin Ping Tan:** visualization, writing – review and editing. **Abu Zahrim Yaser:** visualization, writing – review and editing. **Deni Shidqi Khaerudini:** formal analysis, resources. **Jaka Sunarso:** formal analysis, supervision, writing – original draft, writing – review and editing.

## Funding

This work was supported by the Ministry of Higher Education, Malaysia (FRGS/1/2021/TK0/SWIN/02/4).

## Conflicts of Interest

The authors declare no conflicts of interest.

## Supporting information


**Figure S1:** Schematic diagram of various breakdown processes in emulsified oily wastewater (Tadros 2013).
**Figure S2:** Three custom‐made samplers: (a) Sampler A for profiling the formation of different layers (thickness) in the grease traps, (b) Sampler B for collecting the floated scum samples at the top layer, (c) Sampler C for collecting the suspended solid–liquid wastewater and settled sludge samples at the middle and bottom layers, respectively (Tang et al. 2024).
**Figure S3:** Schematic diagrams illustrating (a) the Plan and (b) Section A‐A views of the four chosen spots at the inlet and outlet chambers for daily monitoring and sampling using different samplers. All dimensions labeled are in mm (Tang et al. 2024).
**Figure S4:** Sedimentation studies apparatus (SOLTEQ, Model TR01, Malaysia) adapted for CTs in this work.
**Figure S5:** Schematic diagram illustrating the (a) side view and (b, c) separated chamber view of a circular grease trap with the floated scum, clarified liquid (stable suspension), and settled sludge separating and accumulating at their top, middle, and bottom layers (Tang et al. 2024).
**Figure S6:** Settling trends of settled sludge of (a) inlet and (c) outlet chambers for Grease Traps A to D; Floating trends of the floated scum of (b) inlet and (d) outlet chambers for Grease Traps A to D. [Note: Inlet chamber results were taken based on the average of C1 and C2 results].
**Figure S7:** Determination of critical velocities using (a) flotation and (b) sedimentation kinetics for inlet and outlet chambers. The steepest gradients were utilized to calculate mean flotation velocities (1.79 and 0.90 m h^−1^) and mean sedimentation velocities (2.59 and 2.28 m h^−1^) across Greaes Traps A–D.
**Figure S8:** Settling velocity (m h^−1^) of the (a) C1 and C2 inlet and (b) C1 outlet samples for Grease Traps A to D; Floating velocity (m h^−1^) of the (c) C1 and C2 inlet and (d) C1 outlet samples for Grease Traps A to D.
**Figure S9:** Average settling velocity (m h^−1^) of the (a) C1 and C2 inlet and (b) C1 outlet samples for Grease Traps A to D; Floating velocity (m h^−1^) of the (c) C1 and C2 inlet and (d) C1 outlet samples for Grease Traps A to D.
**Figure S10:** Time‐lapse photography of the column test for Sample A‐R1‐I‐C2, illustrating the transition from initial five‐layer stratification (< 5 min) to the consolidation of three distinct layers by 20 min.
**Figure S11:** Schematic representation of particle sedimentation principles used to derive the critical grease trap design criteria, including the surface overflow (SOR) and hydraulic retention time (HRT).
**Table S1:** Thickness (D, mm) and profile composition (%) of the top (T), middle (M), and bottom (B) layers for Grease Traps A and B, including those inside the inlet (I) and outlet (O) chambers.
**Table S2:** Thickness (D, mm) and profile composition (%) of the top (T), middle (M), and bottom (B) layers for Grease Traps C and D, including those inside the inlet (I) and outlet (O) chambers.
**Table S3:** Estimated HRT for Grease Traps A to D based on 500 L working volume and MYR36 monthly water bill per stall and eight operating hours to estimate the flow rates (Tang et al. 2024).
**Table S4:** Comparison of SI and CT results on their average layer thickness (T, cm) and profile distribution (%) at the top (T), middle (M), and bottom (B) layers for Grease Traps A to D.
**Table S5:** Settling and floating velocity (m h^−1^) over the elapsed time (min) in Grease Trap A.
**Table S6:** Settling and floating velocity (m h^−1^) over the elapsed time (min) in Grease Trap B.
**Table S7:** Settling and floating velocity (m h^−1^) over the elapsed time (min) in Grease Trap C.
**Table S8:** Settling and floating velocity (m h^−1^) over the elapsed time (min) in Grease Trap D.
**Table S9:** Photographs of column tests (CTs) for Round 1 (R1) samples collected from Grease Traps A, including Column 1 (C1) for both inlet (I) and outlet (O) chamber samples and Column 2 (C2) for inlet (I) chamber samples only.
**Table S10:** Photographs of column tests (CTs) for Round 2 (R2) samples collected from Grease Traps A, including Column 1 (C1) for both inlet (I) and outlet (O) chamber samples and Column 2 (C2) for inlet (I) chamber samples only.
**Table S11:** Photographs of column tests (CTs) for Round 1 (R1) samples collected from Grease Traps B, including Column 1 (C1) for both inlet (I) and outlet (O) chamber samples and Column 2 (C2) for inlet (I) chamber samples only.
**Table S12:** Photographs of column tests (CTs) for Round 2 (R1) samples collected from Grease Traps B, including Column 1 (C1) for both inlet (I) and outlet (O) chamber samples and Column 2 (C2) for inlet (I) chamber samples only.
**Table S13:** Photographs of column tests (CTs) for Round 1 (R1) samples collected from Grease Traps C, including Column 1 (C1) for both inlet (I) and outlet (O) chamber samples and Column 2 (C2) for inlet (I) chamber samples only.
**Table S14:** Photographs of column tests (CTs) for Round 2 (R2) samples collected from Grease Traps C, including Column 1 (C1) for both inlet (I) and outlet (O) chamber samples and Column 2 (C2) for inlet (I) chamber samples only.
**Table S15:** Photographs of column tests (CTs) for Round 1 (R1) samples collected from Grease Traps D, including Column 1 (C1) for both inlet (I) and outlet (O) chamber samples and Column 2 (C2) for inlet (I) chamber samples only.
**Table S16:** Photographs of column tests (CTs) for Round 2 (R2) samples collected from Grease Traps D, including Column 1 (C1) for both inlet (I) and outlet (O) chamber samples and Column 2 (C2) for inlet (I) chamber samples only.

## Data Availability

Data can be made available on request.
