# Supplementary material for: Elucidation of the Physical Separation and Accumulation Mechanisms of Three Distinct Layers in Grease Traps
Source: Water Environ Res. 2026 Jul 29;98(8):e70511. doi: 10.1002/wer.70511 (PMC13420775; doi:10.1002/wer.70511)
Supplement: Supplementary file 1 — Figure S1: Schematic diagram of various breakdown processes in emulsified oily wastewater (Tadros 2013). Figure S2: Three custom‐made samplers: (a) Sampler A for profiling the formation of different layers (thickness) in the grease traps, (b) Sampler B for collecting the floated scum samples at the top layer, (c) Sampler C for collecting the suspended solid–liquid wastewater and settled sludge samples at the middle and bottom layers, respectively (Tang et al. 2024). Figure S3: Schematic diagrams illustrating (a) the Plan and (b) Section A‐A views of the four chosen spots at the inlet and outlet chambers for daily monitoring and sampling using different samplers. All dimensions labeled are in mm (Tang et al. 2024). Figure S4: Sedimentation studies apparatus (SOLTEQ, Model TR01, Malaysia) adapted for CTs in this work. Figure S5: Schematic diagram illustrating the (a) side view and (b, c) separated chamber view of a circular grease trap with the floated scum, clarified liquid (stable suspension), and settled sludge separating and accumulating at their top, middle, and bottom layers (Tang et al. 2024). Figure S6: Settling trends of settled sludge of (a) inlet and (c) outlet chambers for Grease Traps A to D; Floating trends of the floated scum of (b) inlet and (d) outlet chambers for Grease Traps A to D. [Note: Inlet chamber results were taken based on the average of C1 and C2 results]. Figure S7: Determination of critical velocities using (a) flotation and (b) sedimentation kinetics for inlet and outlet chambers. The steepest gradients were utilized to calculate mean flotation velocities (1.79 and 0.90 m h−1) and mean sedimentation velocities (2.59 and 2.28 m h−1) across Greaes Traps A–D. Figure S8: Settling velocity (m h−1) of the (a) C1 and C2 inlet and (b) C1 outlet samples for Grease Traps A to D; Floating velocity (m h−1) of the (c) C1 and C2 inlet and (d) C1 outlet samples for Grease Traps A to D. Figure S9: Average settling velocity (m h−1) of the (a) C1 and C2 i [file WER-98-e70511-s001.docx]

Supplementary Materials

Elucidation of the physical separation and accumulation mechanisms of three distinct layers in grease traps

Ling Ying Tang^a^, Alex Kwong Jun Kiu^a^, Ngie Hing Wong^a,^*, Chung Siung Choo^b^, Lily Li^c^, Chin Ping Tan^d^, Abu Zahrim Yaser^e^, Deni Shidqi Khaerudini^f^, Jaka Sunarso^a^

*^a^Research Centre for Sustainable Technologies, Faculty of Engineering, Computing and Science, Swinburne University of Technology, Jalan Simpang Tiga, 93350 Kuching, Sarawak, Malaysia*

*^b^Centre for Innovative Society, Faculty of Engineering, Computing, and Science, Swinburne University of Technology, Jalan Simpang Tiga, 93350 Kuching, Sarawak, Malaysia*

*^c^Centre of Smart Infrastructure and Digital Construction, Department of Civil and Construction Engineering, Swinburne University of Technology, Victoria 3122, Australia*

*^d^Department of Food Technology, Faculty of Food Science and Technology, Universiti Putra Malaysia, Selangor, Serdang, 43400, Malaysia*

*^e^Faculty of Engineering, Universiti Malaysia Sabah, Jalan UMS, 88400 Kota Kinabalu, Sabah, Malaysia*

*^f^Research Center for Energy Conversion and Conservation, National Research and Innovation Agency (BRIN), Bld. 625 National Science Techno Park BJ Habibie, South Tangerang 15314 Banten, Indonesia*

- Figures -


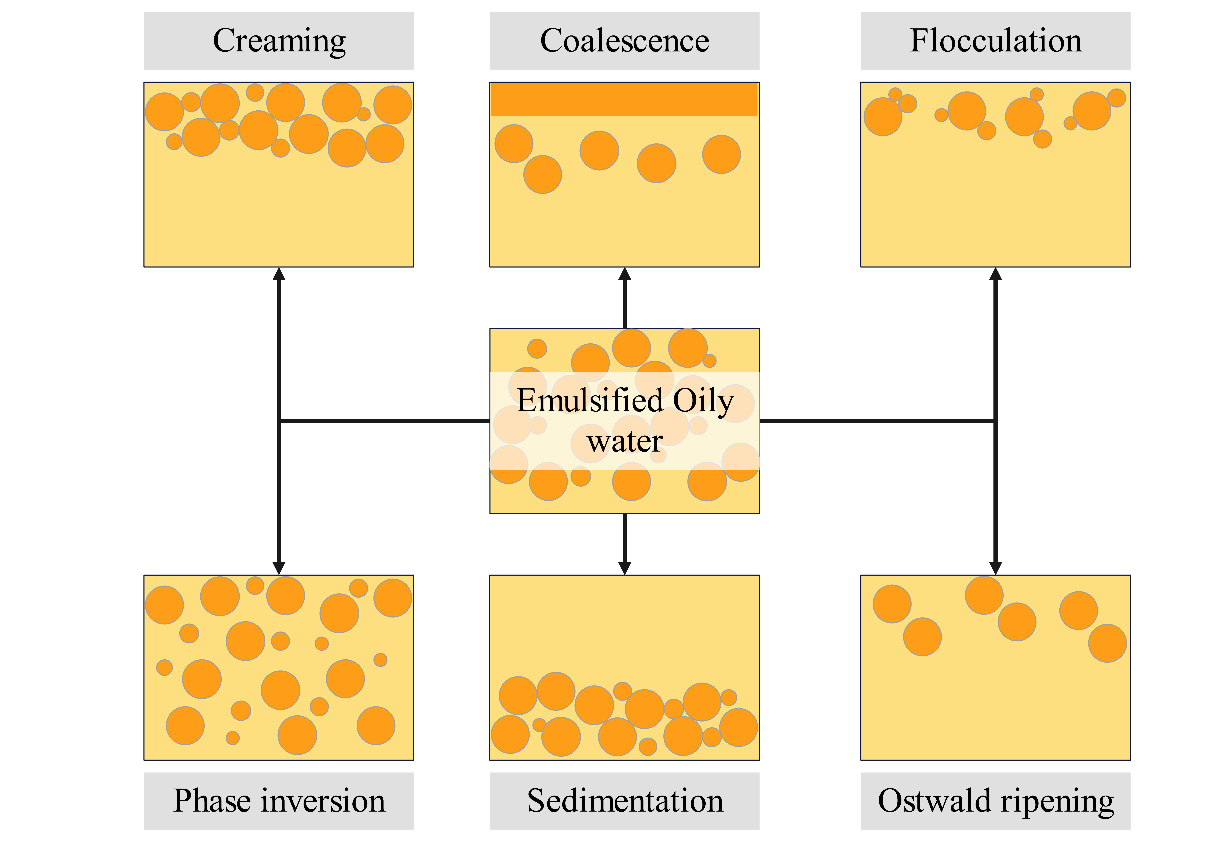


**Fig. S1.** Schematic diagram of various breakdown processes in emulsified oily wastewater (Tadros 2013).


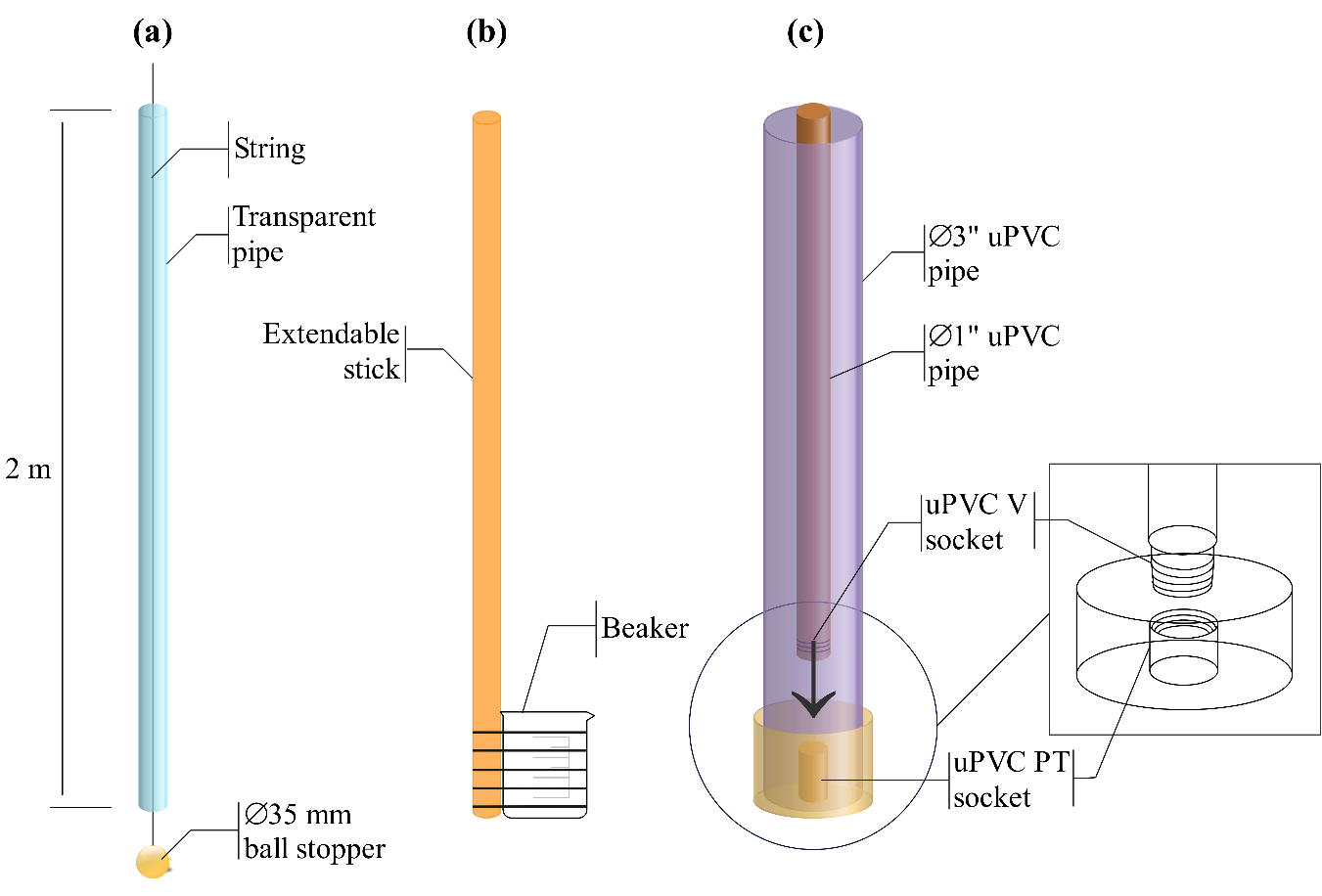


**Fig. S2.** Three custom-made samplers: (a) Sampler A for profiling the formation of different layers (thickness) in the grease traps, (b) Sampler B for collecting the floated scum samples at the top layer, (c) Sampler C for collecting the suspended solid-liquid wastewater and settled sludge samples at the middle and bottom layers, respectively (Tang et al. 2024).


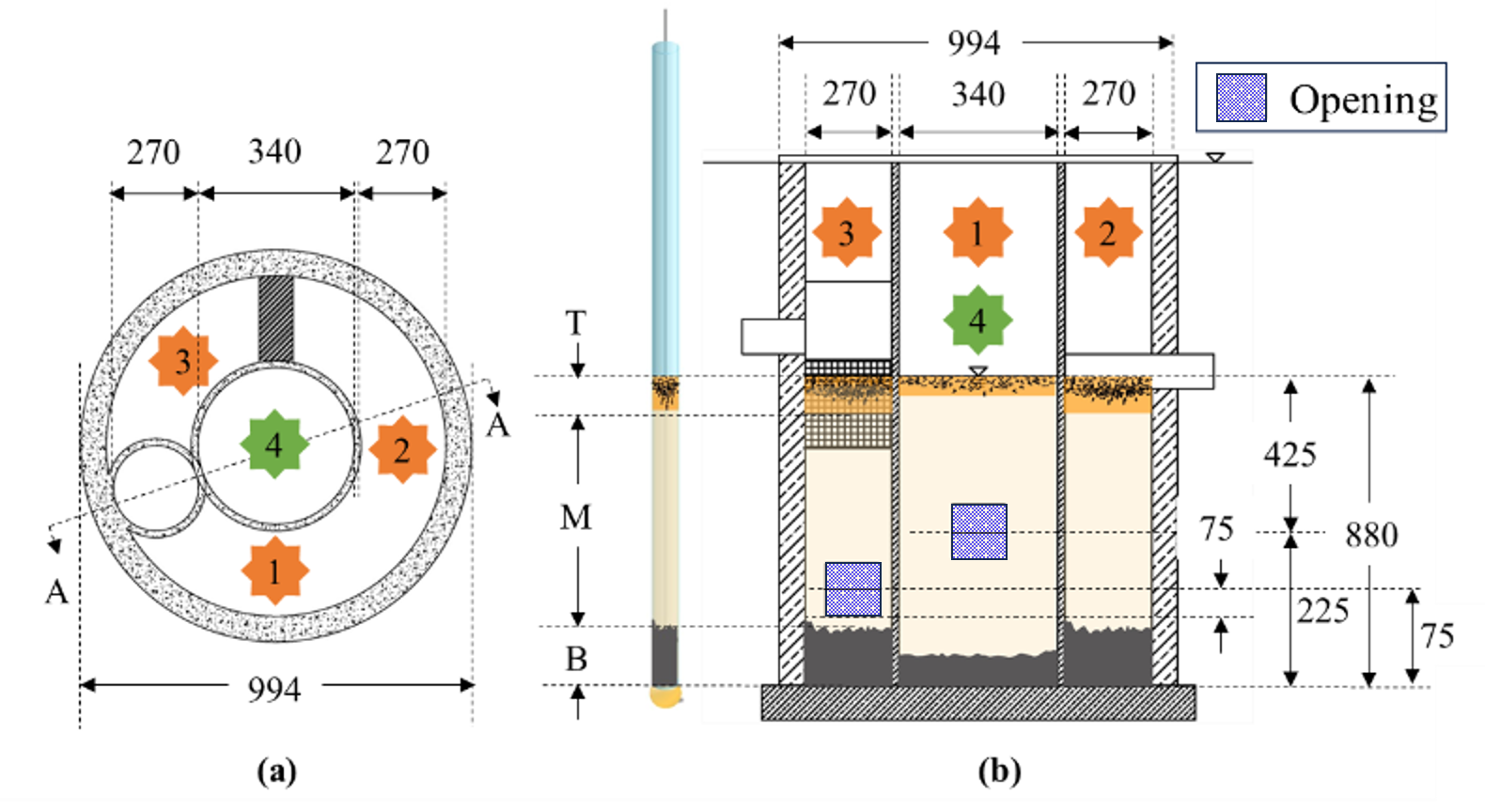


**Fig. S3.** Schematic diagrams illustrating (a) the Plan and (b) Section A-A views of the four chosen spots at the inlet and outlet chambers for daily monitoring and sampling using different samplers. All dimensions labeled are in mm (Tang et al. 2024).


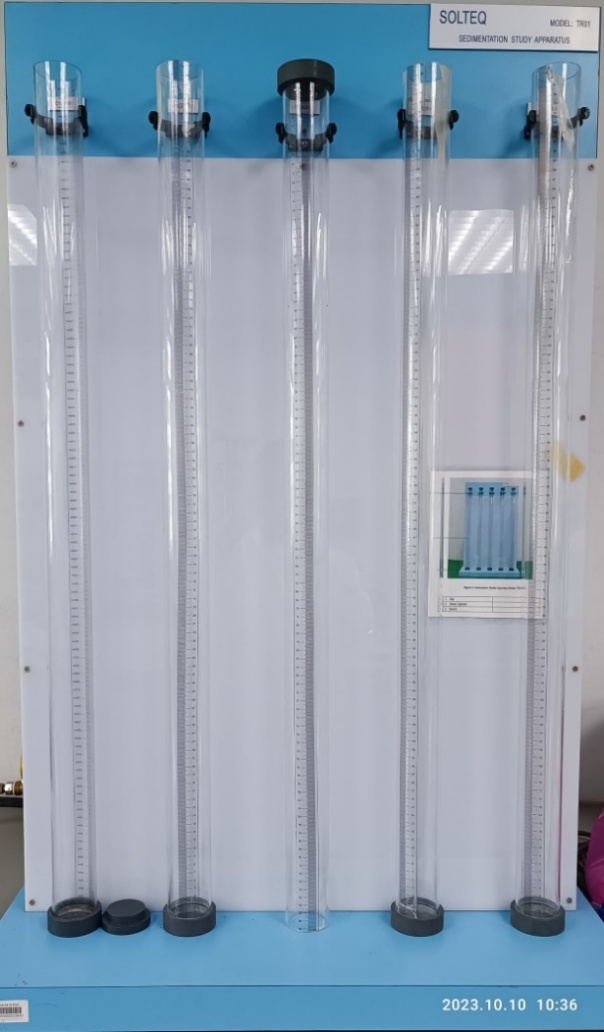


**Fig. S4.** Sedimentation studies apparatus (SOLTEQ, Model TR01, Malaysia) adapted for CTs in this work.


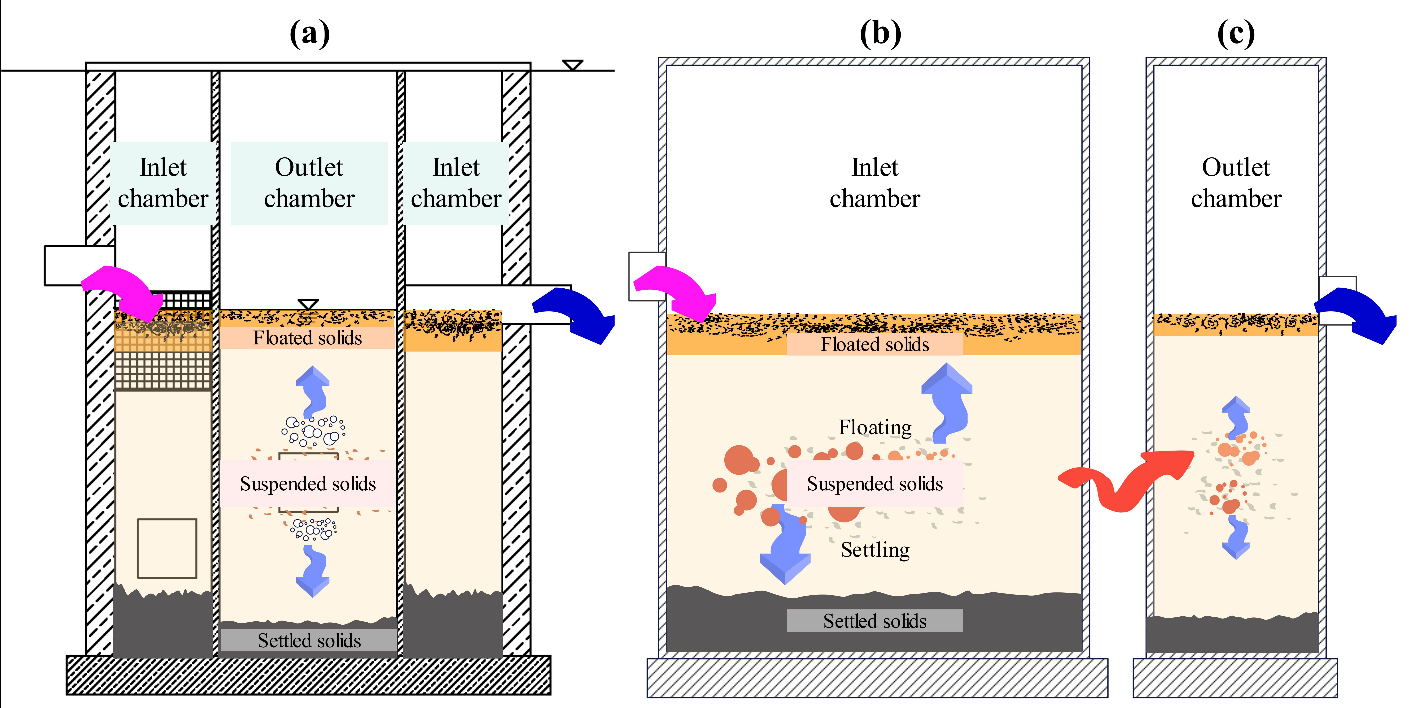


**Fig. S5.** Schematic diagram illustrating the (a) side view and (b, c) separated chamber view of a circular grease trap with the floated scum, clarified liquid (stable suspension), and settled sludge separating and accumulating at their top, middle, and bottom layers (Tang et al. 2024).


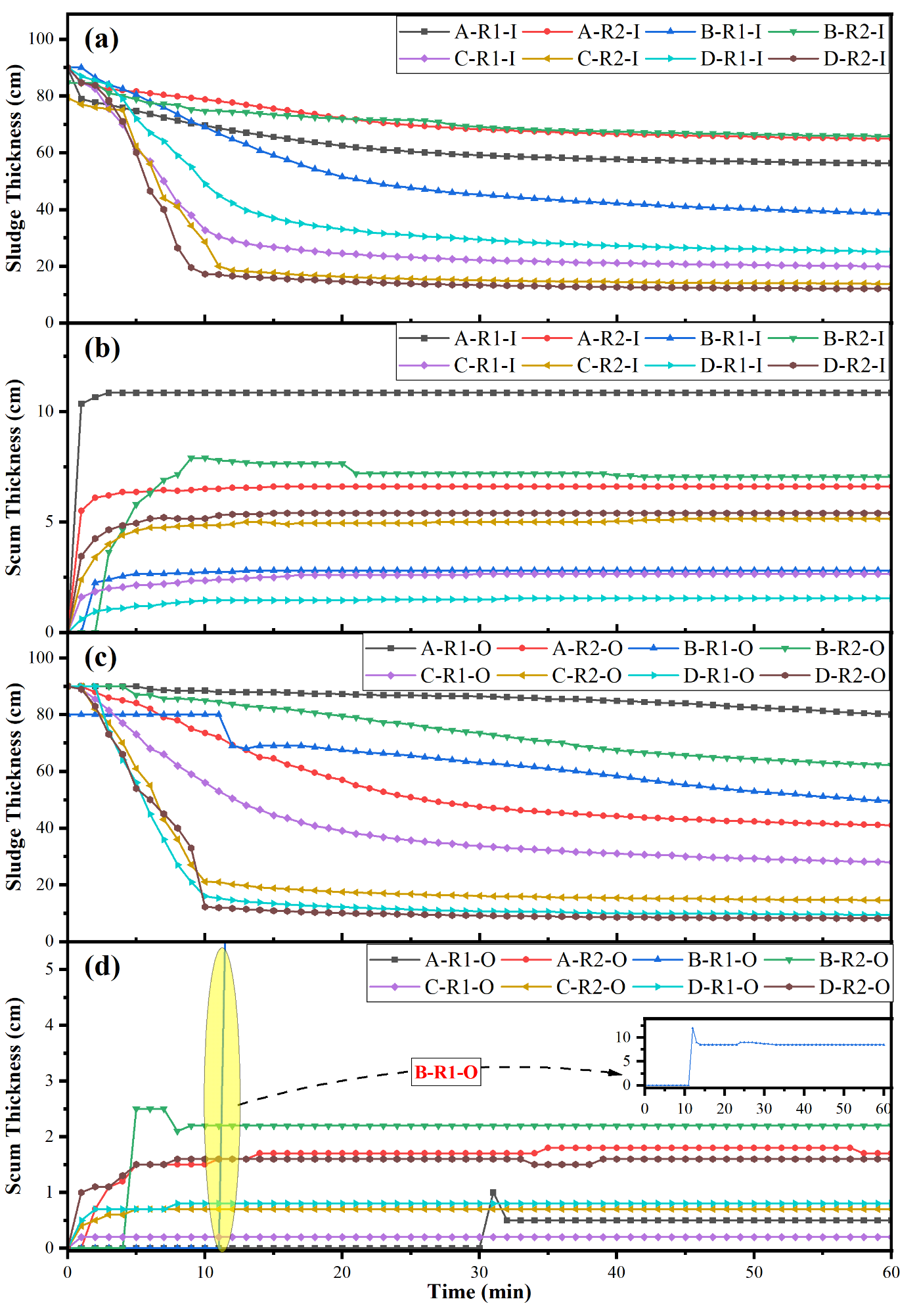


**Fig. S6.** Settling trends of settled sludge of (a) inlet and (c) outlet chambers for Grease Traps A to D; Floating trends of the floated scum of (b) inlet and (d) outlet chambers for Grease Traps A to D. [Note: Inlet chamber results were taken based on the average of C1 and C2 results]

| 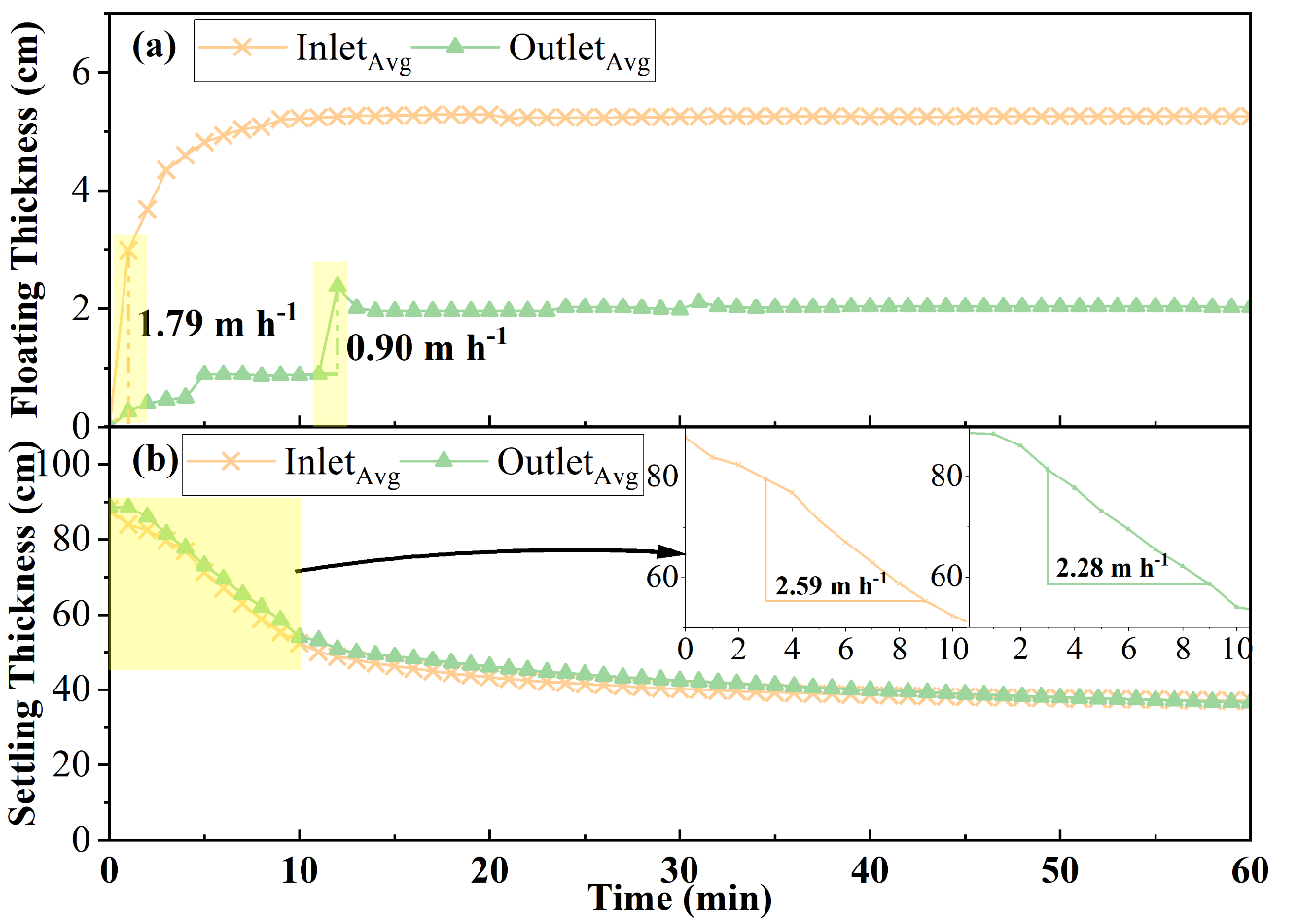 |
| --- |

**Fig. S7.** Determination of critical velocities using (a) flotation and (b) sedimentation kinetics for inlet and outlet chambers. The steepest gradients were utilized to calculate mean flotation velocities (1.79 and 0.90 m h^-1^) and mean sedimentation velocities (2.59 and 2.28 m h^-1^) across Greaes Traps A‒D.


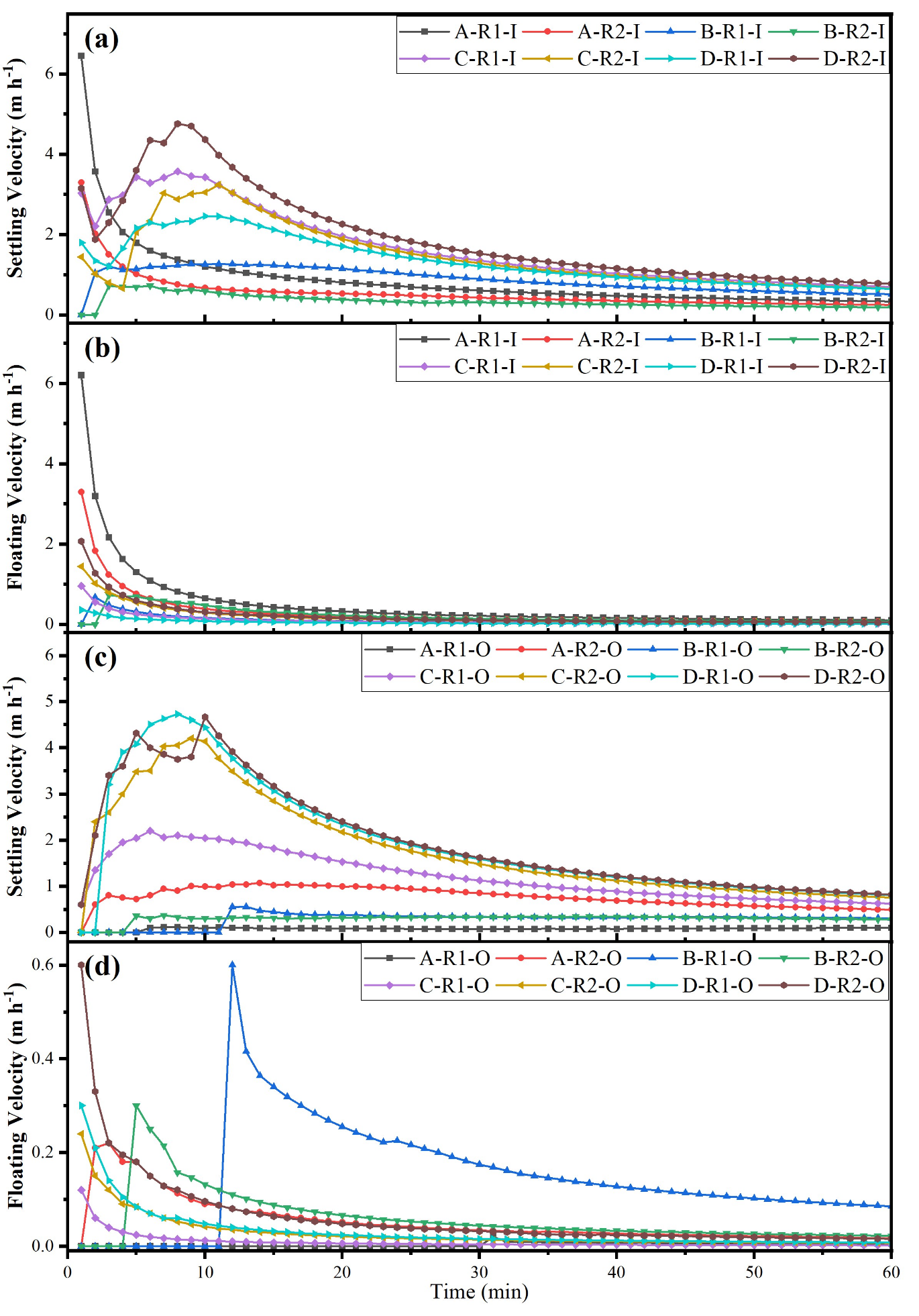


**Fig.** **S8.** Settling velocity (m h⁻¹) of the (a) C1 & C2 inlet and (b) C1 outlet samples for Grease Traps A to D; Floating velocity m h⁻¹) of the (c) C1 & C2 inlet and (d) C1 outlet samples for Grease Traps A to D.


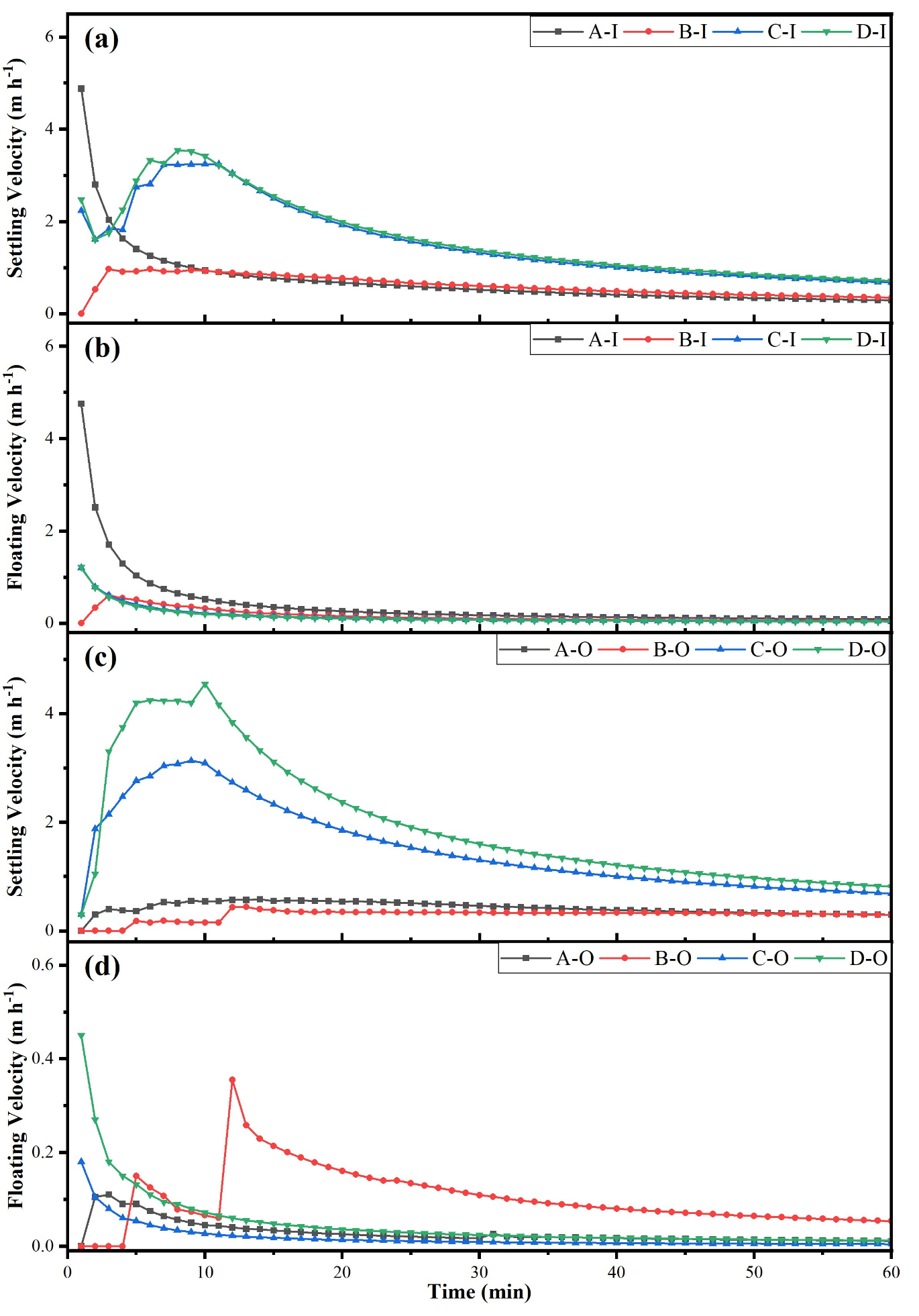


**Fig. S9.** Average settling velocity (m h⁻¹) of the (a) C1 & C2 inlet and (b) C1 outlet samples for Grease Traps A to D; Floating velocity (m h⁻¹) of the (c) C1 & C2 inlet and (d) C1 outlet samples for Grease Traps A to D.


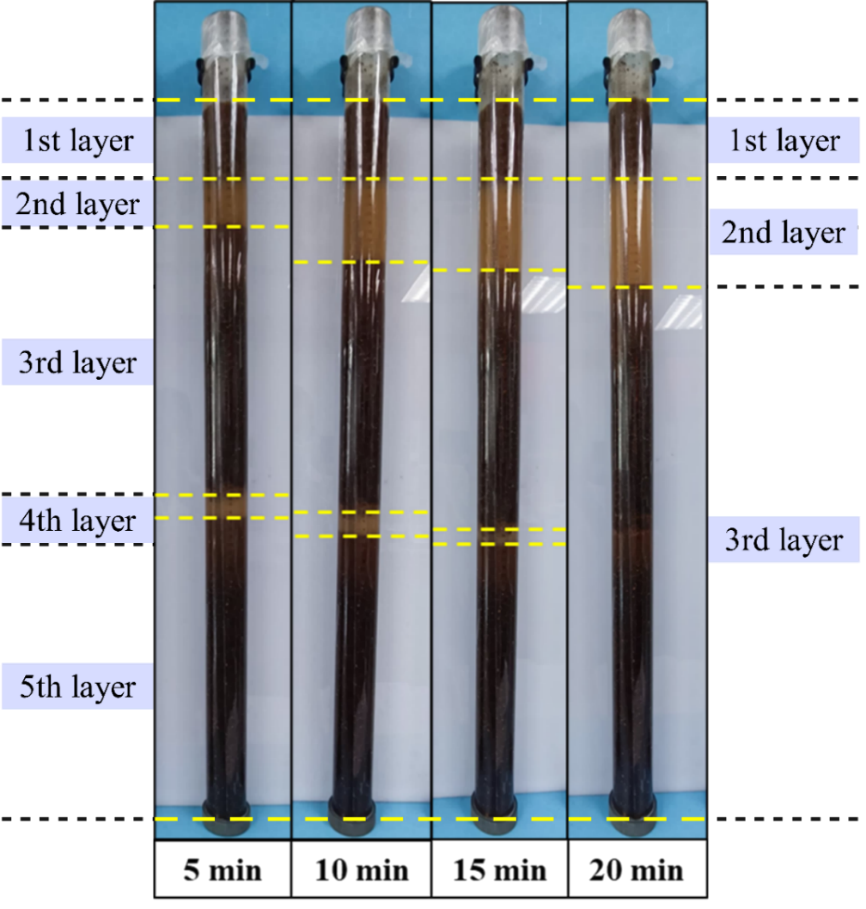


**Fig. S10.** Time-lapse photography of the column test for Sample A-R1-I-C2, illustrating the transition from initial five-layer stratification (< 5 minutes) to the consolidation of three distinct layers by 20 minutes.


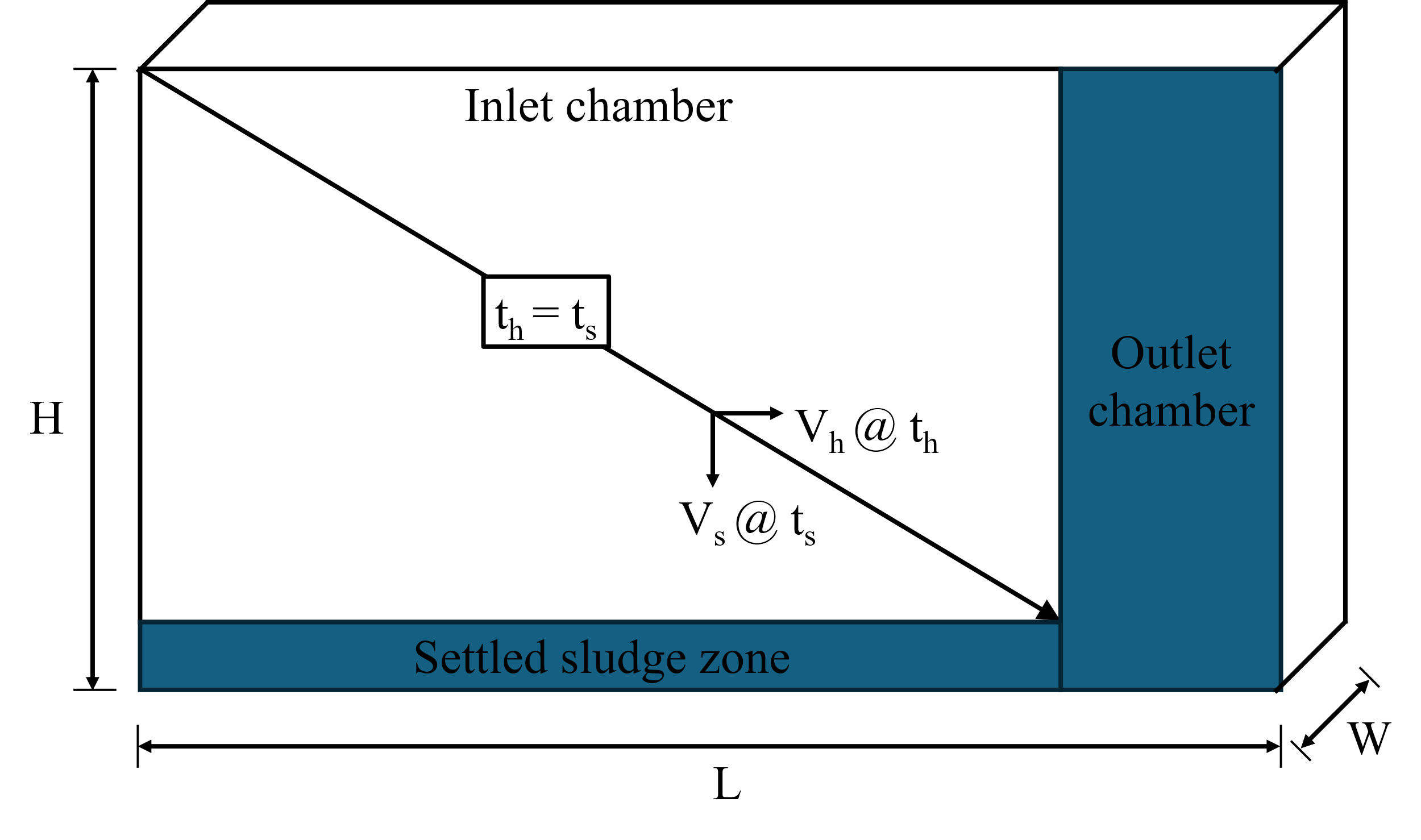


**Fig. S11.** Schematic representation of particle sedimentation principles used to derive the critical grease trap design criteria, including the surface overflow (SOR) and hydraulic retention time (HRT).

- Tables -

**Table S1**. Thickness (D, mm) and profile composition (%) of the top (T), middle (M), and bottom (B) layers for Grease Traps A & B, including those inside the inlet (I) and outlet (O) chambers.

|  |  | **A-I (mm)** | | | **A-I (%)** | | | **A-O (mm)** | | | **A-O (%)** | | |  | **B-I (mm)** | | | **B-I (%)** | | | **B-O (mm)** | | | **B-O (%)** | | |
| --- | --- | --- | --- | --- | --- | --- | --- | --- | --- | --- | --- | --- | --- | --- | --- | --- | --- | --- | --- | --- | --- | --- | --- | --- | --- | --- |
| **Day** | **D** | **T** | **B** | **M** | **T** | **B** | **M** | **T** | **B** | **M** | **T** | **B** | **M** | **D** | **T** | **B** | **M** | **T** | **B** | **M** | **T** | **B** | **M** | **T** | **B** | **M** |
| 1 | 845 | 10 | 120 | 715 | 1.18 | 14.2 | 84.6 | 2 | 275 | 568 | 0.24 | 32.5 | 67.2 | 845 | 16 | 168 | 660 | 1.89 | 19.9 | 78.2 | 1 | 265 | 579 | 0.12 | 31.4 | 68.5 |
| 2 | 845 | 9 | 103 | 733 | 1.07 | 12.2 | 86.7 | 2 | 225 | 618 | 0.24 | 26.6 | 73.1 | 855 | 5 | 195 | 655 | 0.58 | 22.8 | 76.6 | 1 | 345 | 509 | 0.12 | 40.4 | 59.5 |
| 3 | 850 | 9 | 95 | 746 | 1.06 | 11.2 | 87.8 | 4 | 230 | 616 | 0.47 | 27.1 | 72.5 | 855 | 13 | 250 | 592 | 1.52 | 29.2 | 69.2 | 1 | 570 | 284 | 0.12 | 66.7 | 33.2 |
| 4 | 845 | 5 | 93 | 747 | 0.59 | 11.1 | 88.4 | 12 | 295 | 538 | 1.42 | 34.9 | 63.7 | 845 | 2 | 235 | 608 | 0.24 | 27.8 | 72.0 | 1 | 435 | 409 | 0.12 | 51.5 | 48.4 |
| 5 | 855 | 4 | 90 | 761 | 0.51 | 10.5 | 89.0 | 5 | 180 | 670 | 0.58 | 21.1 | 78.4 | 835 | 14 | 287 | 534 | 1.68 | 34.3 | 64.0 | 1 | 430 | 404 | 0.12 | 51.5 | 48.4 |
| 6 | 845 | 6 | 90 | 749 | 0.67 | 10.7 | 88.7 | 5 | 165 | 675 | 0.59 | 19.5 | 79.9 | 885 | 19 | 318 | 547 | 2.15 | 36.0 | 61.9 | 1 | 345 | 539 | 0.11 | 39.0 | 60.9 |
| 7 | 850 | 8 | 133 | 708 | 0.98 | 15.7 | 83.3 | 10 | 165 | 675 | 1.18 | 19.4 | 79.4 | 865 | 11 | 165 | 689 | 1.27 | 19.1 | 79.6 | 1 | 375 | 489 | 0.12 | 43.4 | 56.5 |
| 8 | 850 | 7 | 115 | 728 | 0.82 | 13.5 | 85.7 | 15 | 275 | 560 | 1.76 | 32.4 | 65.9 | 875 | 17 | 203 | 654 | 1.94 | 23.2 | 74.8 | 1 | 400 | 474 | 0.11 | 45.7 | 54.2 |
| 9 | 845 | 10 | 75 | 760 | 1.18 | 8.9 | 89.9 | 3 | 125 | 717 | 0.36 | 14.8 | 84.9 | 1320 | 8 | 292 | 1020 | 0.61 | 22.1 | 77.3 | 1 | 625 | 694 | 0.08 | 47.4 | 52.6 |
| 10 | 850 | 9 | 120 | 721 | 1.02 | 14.1 | 84.9 | 2 | 180 | 668 | 0.24 | 21.2 | 78.6 | 1300 | 16 | 495 | 789 | 1.23 | 38.1 | 60.7 | 1 | 599 | 700 | 0.08 | 46.1 | 53.9 |
| 11 | 835 | 9 | 115 | 711 | 1.08 | 13.8 | 85.2 | 12 | 235 | 588 | 1.44 | 28.1 | 70.4 | 845 | 32 | 250 | 563 | 3.79 | 29.6 | 66.6 | 1 | 180 | 664 | 0.12 | 21.3 | 78.6 |
| 12 | 850 | 9 | 117 | 724 | 1.10 | 13.7 | 85.2 | 5 | 250 | 595 | 0.59 | 29.4 | 70.0 | 850 | 29 | 102 | 720 | 3.41 | 12.0 | 84.7 | 1 | 85 | 764 | 0.12 | 10.0 | 89.9 |
| 13 | 835 | 15 | 77 | 743 | 1.80 | 9.18 | 89.0 | 20 | 220 | 595 | 2.40 | 26.4 | 71.3 | 865 | 25 | 147 | 693 | 2.89 | 17.0 | 80.2 | 1 | 75 | 789 | 0.12 | 8.67 | 91.2 |
| 14 | 835 | 7 | 107 | 722 | 0.80 | 12.8 | 86.4 | 3 | 140 | 692 | 0.36 | 16.8 | 82.9 | 845 | 36 | 157 | 653 | 4.26 | 18.5 | 77.2 | 1 | 155 | 689 | 0.12 | 18.3 | 81.5 |
| 15 | 845 | 2 | 98 | 744 | 0.28 | 11.6 | 88.1 | 1 | 210 | 634 | 0.12 | 24.9 | 75.0 | 845 | 32 | 153 | 661 | 3.79 | 18.1 | 78.2 | 1 | 115 | 729 | 0.12 | 13.6 | 86.3 |
| 16 | 845 | 8 | 132 | 705 | 0.99 | 15.6 | 83.4 | 10 | 180 | 655 | 1.18 | 21.3 | 77.5 | 840 | 47 | 165 | 628 | 5.60 | 19.6 | 74.7 | 1 | 215 | 624 | 0.12 | 25.6 | 74.3 |
| 17 | 840 | 1 | 88 | 751 | 0.12 | 10.5 | 89.4 | 1 | 160 | 679 | 0.12 | 19.1 | 80.8 | 835 | 4 | 132 | 699 | 0.48 | 15.8 | 83.8 | 1 | 175 | 659 | 0.12 | 21.0 | 78.9 |
| 18 | 845 | 1 | 115 | 729 | 0.12 | 13.6 | 86.3 | 1 | 130 | 714 | 0.12 | 15.4 | 84.5 | 835 | 4 | 123 | 708 | 0.48 | 14.8 | 84.8 | 1 | 245 | 589 | 0.12 | 29.3 | 70.5 |
| 19 | 845 | 3 | 128 | 714 | 0.32 | 15.2 | 84.5 | 0 | 115 | 730 | 0.00 | 13.6 | 86.4 | 855 | 6 | 152 | 697 | 0.70 | 17.7 | 81.6 | 0 | 225 | 630 | 0.00 | 26.3 | 73.7 |
| 20 | 855 | 4 | 82 | 769 | 0.51 | 9.6 | 89.9 | 1 | 115 | 739 | 0.12 | 13.5 | 86.4 | 855 | 6 | 172 | 677 | 0.70 | 20.1 | 79.2 | 1 | 260 | 594 | 0.12 | 30.4 | 69.5 |
| 21 | 835 | 8 | 133 | 694 | 0.96 | 16.0 | 83.1 | 5 | 150 | 680 | 0.60 | 18.0 | 81.4 | 840 | 11 | 180 | 649 | 1.31 | 21.4 | 77.3 | 1 | 250 | 589 | 0.12 | 29.8 | 70.1 |
| 22 | 830 | 11 | 92 | 727 | 1.37 | 11.0 | 87.6 | 3 | 155 | 672 | 0.36 | 18.7 | 81.0 | 845 | 11 | 185 | 649 | 1.30 | 21.9 | 76.8 | 1 | 230 | 614 | 0.12 | 27.2 | 72.7 |
| 23 | 845 | 3 | 97 | 746 | 0.32 | 11.4 | 88.2 | 0 | 200 | 645 | 0.00 | 23.7 | 76.3 | 845 | 11 | 145 | 689 | 1.30 | 17.2 | 81.5 | 1 | 410 | 434 | 0.12 | 48.5 | 51.4 |
| 24 | 845 | 10 | 118 | 716 | 1.22 | 14.0 | 84.8 | 1 | 175 | 669 | 0.12 | 20.7 | 79.2 | 845 | 13 | 180 | 652 | 1.54 | 21.3 | 77.2 | 1 | 390 | 454 | 0.12 | 46.2 | 53.7 |
| 25 | 835 | 11 | 118 | 706 | 1.32 | 14.2 | 84.5 | 1 | 195 | 639 | 0.12 | 23.4 | 76.5 | 845 | 14 | 235 | 596 | 1.66 | 27.8 | 70.6 | 1 | 180 | 664 | 0.12 | 21.3 | 78.6 |
| 26 | 825 | 8 | 118 | 699 | 0.97 | 14.3 | 84.7 | 1 | 175 | 649 | 0.12 | 21.2 | 78.7 | 850 | 12 | 192 | 646 | 1.41 | 22.6 | 76.0 | 0 | 410 | 440 | 0.00 | 48.2 | 51.8 |
| 27 | 825 | 7 | 122 | 696 | 0.89 | 14.8 | 84.4 | 3 | 165 | 657 | 0.36 | 20.0 | 79.6 | 850 | 19 | 232 | 599 | 2.24 | 27.3 | 70.5 | 1 | 530 | 319 | 0.12 | 62.4 | 37.5 |
| 28 | 835 | 9 | 108 | 717 | 1.12 | 13.0 | 85.9 | 1 | 165 | 669 | 0.12 | 19.8 | 80.1 | 845 | 23 | 227 | 595 | 2.72 | 26.8 | 70.4 | 2 | 545 | 298 | 0.24 | 64.5 | 35.3 |
| 29 | 825 | 7 | 123 | 695 | 0.81 | 15.0 | 84.2 | 5 | 175 | 645 | 0.61 | 21.2 | 78.2 | 835 | 19 | 185 | 631 | 2.28 | 22.2 | 75.5 | 1 | 625 | 209 | 0.12 | 74.9 | 25.0 |
| 30 | 835 | 9 | 145 | 681 | 1.08 | 17.4 | 81.6 | 2 | 205 | 628 | 0.24 | 24.6 | 75.2 | 845 | 20 | 150 | 675 | 2.37 | 17.8 | 79.9 | 1 | 605 | 239 | 0.12 | 71.6 | 28.3 |
| 31 | 830 | 8 | 102 | 720 | 1.00 | 12.3 | 86.8 | 1 | 195 | 634 | 0.12 | 23.5 | 76.4 | 840 | 22 | 205 | 613 | 2.62 | 24.4 | 73.0 | 1 | 580 | 259 | 0.12 | 69.1 | 30.8 |
| 32 | 835 | 12 | 100 | 723 | 1.40 | 12.0 | 86.6 | 7 | 175 | 653 | 0.84 | 21.0 | 78.2 | 855 | 21 | 190 | 644 | 2.46 | 22.2 | 75.3 | 1 | 430 | 424 | 0.12 | 50.3 | 49.6 |
| Avg | 841 | 7 | 108 | 725 | 0.89 | 12.9 | 86.2 | 5 | 188 | 649 | 0.53 | 22.3 | 77.2 | 878 | 17 | 202 | 659 | 1.95 | 22.8 | 75.3 | 1 | 353 | 524 | 0.11 | 40.0 | 59.9 |
| Max | 855 | 15 | 145 | 769 | 1.80 | 17.4 | 89.9 | 20 | 295 | 739 | 2.40 | 34.9 | 86.4 | 1320 | 47 | 495 | 1020 | 5.60 | 38.1 | 84.8 | 2 | 625 | 789 | 0.24 | 74.9 | 91.2 |
| Min | 825 | 1 | 75 | 681 | 0.12 | 8.88 | 81.6 | 0 | 115 | 538 | 0.00 | 13.5 | 63.7 | 835 | 2 | 102 | 534 | 0.24 | 12.0 | 60.7 | 0 | 75 | 209 | 0.00 | 8.67 | 25.0 |
| Std err | 1.51 | 0.57 | 3.11 | 3.87 | 0.07 | 0.38 | 0.39 | 0.85 | 8.06 | 8.42 | 0.10 | 0.95 | 1.00 | 20.1 | 1.81 | 13.0 | 14.9 | 0.22 | 1.08 | 1.08 | 0.05 | 30.0 | 28.8 | 0.01 | 3.27 | 3.27 |

**Table S2**. Thickness (D, mm) and profile composition (%) of the top (T), middle (M), and bottom (B) layers for Grease Traps C & D, including those inside the inlet (I) and outlet (O) chambers.

|  |  | **C-I (mm)** | | | **C-I (%)** | | | **C-O (mm)** | | | **C-O (%)** | | |  | **D-I (mm)** | | | **D-I (%)** | | | **D-O (mm)** | | | **D-O (%)** | | |
| --- | --- | --- | --- | --- | --- | --- | --- | --- | --- | --- | --- | --- | --- | --- | --- | --- | --- | --- | --- | --- | --- | --- | --- | --- | --- | --- |
| **Day** | **D** | **T** | **B** | **M** | **T** | **B** | **M** | **T** | **B** | **M** | **T** | **B** | **M** | **D** | **T** | **B** | **M** | **T** | **B** | **M** | **T** | **B** | **M** | **T** | **B** | **M** |
| 1 | 825 | 15 | 25 | 784 | 1.82 | 3.07 | 95.1 | 1 | 35 | 789 | 0.12 | 4.24 | 95.6 | 855 | 12 | 145 | 698 | 1.40 | 16.2 | 82.4 | 15 | 200 | 640 | 1.75 | 23.4 | 74.9 |
| 2 | 835 | 17 | 45 | 773 | 2.04 | 5.39 | 92.6 | 1 | 65 | 769 | 0.12 | 7.78 | 92.1 | 845 | 15 | 110 | 720 | 1.78 | 13.0 | 85.2 | 15 | 185 | 645 | 1.78 | 21.9 | 76.3 |
| 3 | 835 | 11 | 40 | 784 | 1.32 | 4.83 | 93.9 | 1 | 15 | 819 | 0.12 | 1.80 | 98.1 | 850 | 21 | 127 | 702 | 2.47 | 14.9 | 82.6 | 7 | 205 | 638 | 0.82 | 24.1 | 75.1 |
| 4 | 835 | 11 | 43 | 781 | 1.32 | 5.19 | 93.5 | 1 | 15 | 819 | 0.12 | 1.80 | 98.1 | 855 | 15 | 130 | 710 | 1.75 | 15.2 | 83.0 | 1 | 200 | 654 | 0.12 | 23.4 | 76.5 |
| 5 | 840 | 13 | 80 | 747 | 1.55 | 9.56 | 88.9 | 1 | 37 | 802 | 0.12 | 4.40 | 95.5 | 855 | 12 | 117 | 726 | 1.40 | 13.7 | 85.0 | 1 | 175 | 679 | 0.12 | 20.5 | 79.4 |
| 6 | 815 | 8 | 19 | 789 | 0.98 | 2.29 | 96.8 | 1 | 20 | 794 | 0.12 | 2.45 | 97.4 | 845 | 10 | 122 | 714 | 1.18 | 14.4 | 84.5 | 1 | 355 | 489 | 0.12 | 42.0 | 57.9 |
| 7 | 830 | 18 | 23 | 789 | 2.17 | 2.81 | 95.1 | 1 | 15 | 814 | 0.12 | 1.81 | 98.1 | 845 | 15 | 138 | 692 | 1.78 | 16.4 | 81.9 | 10 | 225 | 610 | 1.18 | 26.6 | 72.2 |
| 8 | 825 | 13 | 85 | 727 | 1.58 | 10.3 | 88.1 | 1 | 10 | 814 | 0.12 | 1.21 | 98.7 | 855 | 10 | 116 | 729 | 1.17 | 13.6 | 85.3 | 1 | 240 | 614 | 0.12 | 28.1 | 71.8 |
| 9 | 815 | 9 | 31 | 775 | 1.10 | 3.76 | 95.1 | 1 | 65 | 749 | 0.12 | 7.98 | 91.9 | 850 | 16 | 120 | 714 | 1.88 | 14.1 | 84.0 | 1 | 200 | 649 | 0.12 | 23.5 | 76.4 |
| 10 | 835 | 14 | 50 | 771 | 1.68 | 5.99 | 92.3 | 1 | 10 | 824 | 0.12 | 1.20 | 98.7 | 855 | 21 | 150 | 684 | 2.46 | 17.5 | 80.0 | 5 | 235 | 615 | 0.58 | 27.5 | 71.9 |
| 11 | 835 | 9 | 63 | 762 | 1.08 | 7.58 | 91.3 | 1 | 35 | 799 | 0.12 | 4.19 | 95.7 | 855 | 15 | 108 | 732 | 1.75 | 12.7 | 85.6 | 1 | 270 | 584 | 0.12 | 31.6 | 68.3 |
| 12 | 845 | 14 | 65 | 766 | 1.66 | 7.69 | 90.7 | 1 | 25 | 819 | 0.12 | 2.96 | 96.9 | 855 | 31 | 117 | 707 | 3.63 | 13.7 | 82.7 | 5 | 410 | 440 | 0.58 | 48.0 | 51.5 |
| 13 | 830 | 13 | 88 | 730 | 1.57 | 10.6 | 87.9 | 1 | 30 | 799 | 0.12 | 3.61 | 96.3 | 855 | 23 | 122 | 711 | 2.69 | 14.2 | 83.1 | 1 | 345 | 509 | 0.12 | 40.4 | 59.5 |
| 14 | 825 | 9 | 48 | 767 | 1.09 | 5.86 | 93.0 | 1 | 15 | 809 | 0.12 | 1.82 | 98.1 | 855 | 22 | 115 | 718 | 2.57 | 13.5 | 84.0 | 1 | 305 | 549 | 0.12 | 35.7 | 64.2 |
| 15 | 835 | 23 | 127 | 685 | 2.75 | 15.2 | 82.0 | 2 | 5 | 828 | 0.24 | 0.60 | 99.2 | 845 | 17 | 102 | 726 | 2.01 | 12.0 | 85.9 | 1 | 30 | 814 | 0.12 | 3.55 | 96.3 |
| 16 | 845 | 22 | 115 | 708 | 2.60 | 13.6 | 83.8 | 1 | 30 | 814 | 0.12 | 3.55 | 96.3 | 850 | 21 | 90 | 739 | 2.47 | 10.6 | 86.9 | 13 | 40 | 797 | 1.53 | 4.71 | 93.8 |
| 17 | 820 | 20 | 63 | 737 | 2.44 | 7.72 | 89.8 | 1 | 20 | 799 | 0.12 | 2.44 | 97.4 | 855 | 23 | 67 | 766 | 2.69 | 7.80 | 89.6 | 10 | 45 | 800 | 1.17 | 5.26 | 93.6 |
| 18 | 845 | 0 | 23 | 822 | 0.00 | 2.76 | 97.2 | 0 | 15 | 830 | 0.00 | 1.78 | 98.2 | 855 | 2 | 92 | 762 | 0.23 | 10.7 | 89.1 | 3 | 55 | 797 | 0.35 | 6.43 | 93.2 |
| 19 | 835 | 2 | 33 | 800 | 0.24 | 3.99 | 95.8 | 0 | 35 | 800 | 0.00 | 4.19 | 95.8 | 855 | 6 | 42 | 807 | 0.70 | 4.87 | 94.4 | 3 | 20 | 832 | 0.35 | 2.34 | 97.3 |
| 20 | 850 | 2 | 17 | 831 | 0.24 | 1.96 | 97.8 | 1 | 15 | 834 | 0.12 | 1.76 | 98.1 | 855 | 9 | 52 | 794 | 1.05 | 6.04 | 92.9 | 3 | 35 | 817 | 0.35 | 4.09 | 95.6 |
| 21 | 865 | 11 | 42 | 812 | 1.27 | 4.82 | 93.9 | 1 | 25 | 839 | 0.12 | 2.89 | 97.0 | 840 | 9 | 63 | 768 | 1.07 | 7.54 | 91.4 | 1 | 20 | 819 | 0.12 | 2.38 | 97.5 |
| 22 | 835 | 4 | 20 | 811 | 0.48 | 2.44 | 97.1 | 1 | 15 | 819 | 0.12 | 1.80 | 98.1 | 850 | 4 | 56 | 791 | 0.47 | 6.55 | 93.0 | 1 | 25 | 824 | 0.12 | 2.94 | 96.9 |
| 23 | 845 | 6 | 20 | 819 | 0.71 | 2.37 | 97.0 | 1 | 40 | 804 | 0.12 | 4.73 | 95.2 | 845 | 5 | 60 | 780 | 0.59 | 7.10 | 92.4 | 1 | 40 | 804 | 0.12 | 4.73 | 95.2 |
| 24 | 840 | 8 | 28 | 804 | 0.95 | 3.37 | 95.7 | 0 | 75 | 765 | 0.00 | 8.93 | 91.1 | 855 | 6 | 88 | 761 | 0.70 | 10.3 | 89.0 | 1 | 50 | 804 | 0.12 | 5.85 | 94.0 |
| 25 | 845 | 7 | 43 | 794 | 0.83 | 5.13 | 94.0 | 1 | 40 | 804 | 0.12 | 4.73 | 95.2 | 845 | 1 | 83 | 760 | 0.12 | 9.86 | 90.0 | 1 | 45 | 799 | 0.12 | 5.33 | 94.6 |
| 26 | 825 | 11 | 32 | 782 | 1.33 | 3.84 | 94.8 | 1 | 55 | 769 | 0.12 | 6.67 | 93.2 | 850 | 2 | 92 | 756 | 0.24 | 10.8 | 88.9 | 1 | 25 | 824 | 0.12 | 2.94 | 96.9 |
| 27 | 840 | 10 | 43 | 787 | 1.19 | 5.16 | 93.7 | 1 | 30 | 809 | 0.12 | 3.57 | 96.3 | 855 | 6 | 80 | 769 | 0.70 | 9.36 | 90.0 | 1 | 30 | 824 | 0.12 | 3.51 | 96.4 |
| 28 | 835 | 17 | 25 | 793 | 2.04 | 2.99 | 95.0 | 1 | 35 | 799 | 0.12 | 4.19 | 95.7 | 845 | 11 | 95 | 739 | 1.30 | 11.2 | 87.4 | 1 | 65 | 779 | 0.12 | 7.69 | 92.2 |
| 29 | 840 | 13 | 30 | 797 | 1.55 | 3.57 | 94.9 | 0 | 65 | 775 | 0.00 | 7.74 | 92.3 | 835 | 4 | 78 | 753 | 0.48 | 9.38 | 90.1 | 1 | 45 | 789 | 0.12 | 5.39 | 94.5 |
| 30 | 845 | 18 | 53 | 774 | 2.13 | 6.31 | 91.6 | 1 | 35 | 809 | 0.12 | 4.14 | 95.7 | 840 | 8 | 63 | 769 | 0.95 | 7.54 | 91.6 | 1 | 35 | 804 | 0.12 | 4.17 | 95.7 |
| 31 | 830 | 6 | 45 | 779 | 0.72 | 5.42 | 93.9 | 1 | 75 | 754 | 0.12 | 9.04 | 90.8 | 850 | 1 | 85 | 764 | 0.12 | 10.0 | 89.9 | 1 | 40 | 809 | 0.12 | 4.71 | 95.2 |
| 32 | 845 | 17 | 50 | 778 | 2.01 | 5.92 | 92.1 | 1 | 35 | 809 | 0.12 | 4.14 | 95.7 | 855 | 7 | 88 | 760 | 0.82 | 10.3 | 88.9 | 1 | 15 | 839 | 0.12 | 1.75 | 98.1 |
| Avg | 836 | 12 | 47 | 777 | 1.39 | 5.67 | 93.0 | 1 | 32 | 802 | 0.11 | 3.88 | 96.0 | 850 | 12 | 97 | 741 | 1.39 | 11.4 | 87.2 | 3 | 132 | 715 | 0.40 | 15.5 | 84.2 |
| Max | 865 | 23 | 127 | 831 | 2.75 | 15.2 | 97.8 | 2 | 75 | 839 | 0.24 | 9.04 | 99.2 | 855 | 31 | 150 | 807 | 3.63 | 17.5 | 94.4 | 15 | 410 | 839 | 1.78 | 48.0 | 98.1 |
| Min | 815 | 0 | 17 | 685 | 0.00 | 1.96 | 82.0 | 0 | 5 | 749 | 0.00 | 0.60 | 90.8 | 835 | 1 | 42 | 684 | 0.12 | 4.87 | 80.0 | 1 | 15 | 440 | 0.12 | 1.75 | 51.5 |
| Std err | 1.85 | 1.01 | 4.80 | 5.75 | 0.12 | 0.57 | 0.65 | 0.07 | 3.45 | 3.99 | 0.01 | 0.41 | 0.41 | 1.00 | 1.35 | 5.01 | 5.65 | 0.16 | 0.58 | 0.68 | 0.77 | 21.1 | 21.0 | 0.09 | 2.48 | 2.49 |

**Table S3.** Estimated HRT for Grease Traps A to D based on 500 L working volume and MYR36 monthly water bill per stall and eight operating hours to estimate the flow rates (Tang et al. 2024).

| **Parameter** | **Unit** | **Grease Trap A** | **Grease Trap B** | **Grease Trap C** | **Grease Trap D** |
| --- | --- | --- | --- | --- | --- |
| No. F&B stalls | -- | 20 | 16 | 10 | 15 |
| Working volume, V | m^3^ | 0.50 | 0.50 | 0.50 | 0.50 |
| Flowrate, Q | m^3^ d^-1^ | 28.5 | 22.8 | 14.3 | 21.4 |
| HRT = V/Q | h | 0.14 | 0.18 | 0.28 | 0.19 |
| HRT = V/Q | min | 8.40 | 10.5 | 16.8 | 11.2 |

**Table S4.** Comparison of SI and CT results on their average layer thickness (T, cm) and profile distribution (%) at the top (T), middle (M), and bottom (B) layers for Grease Traps A to D.

| **Sample** | **Site Investigation (SI)** | | | | | | **Column Test (CT)** | | | | | |
| --- | --- | --- | --- | --- | --- | --- | --- | --- | --- | --- | --- | --- |
|  | **T (cm)** | **T (%)** | **M (cm)** | **M (%)** | **B (cm)** | **B (%)** | **T (cm)** | **T (%)** | **M (cm)** | **M (%)** | **B (cm)** | **B (%)** |
| A-R1-I | 2.23 | 2.52 | 60.4 | 68.3 | 25.8 | 29.2 | 10.9 | 12.1 | 22.5 | 25.1 | 56.3 | 62.8 |
| A-R2-I | 2.23 | 2.51 | 63.8 | 71.7 | 23.0 | 25.8 | 6.60 | 7.33 | 18.5 | 20.5 | 65.0 | 72.2 |
| A-R1-O | 0.10 | 0.11 | 50.4 | 57.0 | 38.0 | 42.9 | 0.50 | 0.56 | 9.50 | 10.6 | 80.0 | 88.9 |
| A-R2-O | 0.20 | 0.22 | 72.3 | 81.2 | 16.5 | 18.5 | 1.70 | 1.89 | 47.3 | 52.6 | 41.0 | 45.6 |
| B-R1-I | 1.00 | 1.21 | 44.7 | 54.1 | 36.8 | 44.7 | 2.80 | 3.11 | 48.6 | 53.9 | 38.7 | 42.9 |
| B-R2-I | 2.07 | 2.32 | 44.9 | 50.5 | 42.0 | 47.2 | 7.05 | 8.33 | 11.8 | 13.9 | 65.8 | 77.7 |
| B-R1-O | 0.10 | 0.12 | 54.9 | 66.6 | 27.5 | 33.3 | 2.80 | 3.50 | 27.7 | 34.6 | 49.5 | 61.9 |
| B-R2-O | 16.5 | 18.5 | 45.4 | 51.0 | 27.1 | 30.5 | 2.20 | 2.44 | 25.6 | 28.4 | 62.2 | 69.1 |
| C-R1-I | 0.73 | 0.93 | 69.3 | 88.2 | 8.50 | 10.8 | 2.65 | 2.95 | 67.3 | 74.9 | 20.0 | 22.2 |
| C-R2-I | 2.67 | 3.03 | 78.2 | 88.8 | 7.17 | 8.14 | 5.15 | 6.49 | 60.4 | 76.1 | 13.9 | 17.4 |
| C-R1-O | 0.00 | 0.00 | 63.5 | 80.9 | 15.0 | 19.1 | 0.20 | 0.22 | 61.8 | 68.7 | 28.0 | 31.1 |
| C-R2-O | 0.10 | 0.11 | 79.4 | 90.2 | 8.50 | 9.66 | 0.70 | 0.78 | 74.7 | 83.0 | 14.6 | 16.2 |
| D-R1-I | 1.53 | 1.72 | 77.5 | 87.0 | 10.0 | 11.2 | 1.55 | 1.72 | 63.4 | 70.4 | 25.1 | 27.9 |
| D-R2-I | 2.00 | 2.30 | 74.2 | 85.3 | 10.8 | 12.5 | 5.40 | 6.00 | 72.5 | 80.5 | 12.2 | 13.5 |
| D-R1-O | 0.90 | 1.01 | 83.1 | 93.4 | 5.00 | 5.62 | 0.80 | 0.89 | 79.7 | 88.6 | 9.50 | 10.6 |
| D-R2-O | 0.50 | 0.57 | 82.5 | 94.8 | 4.00 | 4.60 | 1.60 | 1.78 | 80.2 | 89.1 | 8.20 | 9.11 |

**Table S5.** Settling and floating velocity (m h⁻¹) over the elapsed time (min) in Grease Trap A.

| **Time (min)** |  |  | **Settling Velocity** | |  |  |  |  | **Floating Velocity** | |  |  |
| --- | --- | --- | --- | --- | --- | --- | --- | --- | --- | --- | --- | --- |
|  | **A-R1-**  **I-C1** | **A-R1-**  **I-C2** | **A-R1-**  **O-C1** | **A-R2-**  **I-C1** | **A-R2-**  **I-C2** | **A-R2-**  **O-C1** | **A-R1-**  **I-C1** | **A-R1-**  **I-C2** | **A-R1-**  **O-C1** | **A-R2-**  **I-C1** | **A-R2-**  **I-C2** | **A-R2-**  **O-C1** |
| 0 | -- | -- | -- | -- | -- | -- | -- | -- | -- | -- | -- | -- |
| 1 | 7.860 | 5.040 | 0.000 | 5.160 | 1.440 | 0.000 | 7.800 | 4.620 | 0.000 | 5.160 | 1.440 | 0.000 |
| 2 | 4.380 | 2.760 | 0.000 | 2.940 | 1.110 | 0.600 | 3.930 | 2.460 | 0.000 | 2.790 | 0.870 | 0.210 |
| 3 | 3.120 | 1.980 | 0.000 | 2.120 | 0.900 | 0.800 | 2.700 | 1.640 | 0.000 | 1.880 | 0.600 | 0.220 |
| 4 | 2.490 | 1.635 | 0.000 | 1.665 | 0.735 | 0.750 | 2.025 | 1.230 | 0.000 | 1.425 | 0.480 | 0.180 |
| 5 | 2.148 | 1.440 | 0.000 | 1.392 | 0.636 | 0.720 | 1.620 | 0.984 | 0.000 | 1.140 | 0.384 | 0.180 |
| 6 | 1.910 | 1.290 | 0.100 | 1.220 | 0.590 | 0.800 | 1.350 | 0.820 | 0.000 | 0.950 | 0.330 | 0.150 |
| 7 | 1.731 | 1.217 | 0.111 | 1.106 | 0.549 | 0.943 | 1.157 | 0.703 | 0.000 | 0.823 | 0.283 | 0.129 |
| 8 | 1.598 | 1.148 | 0.113 | 0.998 | 0.518 | 0.900 | 1.013 | 0.615 | 0.000 | 0.713 | 0.248 | 0.113 |
| 9 | 1.500 | 1.080 | 0.100 | 0.927 | 0.493 | 1.000 | 0.900 | 0.547 | 0.000 | 0.640 | 0.220 | 0.100 |
| 10 | 1.398 | 1.002 | 0.090 | 0.870 | 0.474 | 0.990 | 0.810 | 0.492 | 0.000 | 0.582 | 0.198 | 0.090 |
| 11 | 1.342 | 0.955 | 0.109 | 0.824 | 0.469 | 0.982 | 0.736 | 0.447 | 0.000 | 0.529 | 0.180 | 0.087 |
| 12 | 1.280 | 0.905 | 0.100 | 0.780 | 0.455 | 1.035 | 0.675 | 0.410 | 0.000 | 0.485 | 0.170 | 0.080 |
| 13 | 1.228 | 0.863 | 0.097 | 0.757 | 0.452 | 1.038 | 0.623 | 0.378 | 0.000 | 0.448 | 0.157 | 0.074 |
| 14 | 1.179 | 0.823 | 0.090 | 0.733 | 0.450 | 1.071 | 0.579 | 0.351 | 0.000 | 0.416 | 0.146 | 0.073 |
| 15 | 1.136 | 0.788 | 0.084 | 0.708 | 0.448 | 1.020 | 0.540 | 0.328 | 0.000 | 0.388 | 0.140 | 0.068 |
| 16 | 1.091 | 0.758 | 0.083 | 0.690 | 0.443 | 1.035 | 0.506 | 0.308 | 0.000 | 0.364 | 0.131 | 0.064 |
| 17 | 1.062 | 0.731 | 0.088 | 0.674 | 0.441 | 1.020 | 0.476 | 0.289 | 0.000 | 0.342 | 0.124 | 0.060 |
| 18 | 1.030 | 0.703 | 0.087 | 0.660 | 0.437 | 1.017 | 0.450 | 0.273 | 0.000 | 0.323 | 0.117 | 0.057 |
| 19 | 0.998 | 0.679 | 0.082 | 0.644 | 0.439 | 1.011 | 0.426 | 0.259 | 0.000 | 0.306 | 0.111 | 0.054 |
| 20 | 0.972 | 0.660 | 0.081 | 0.633 | 0.435 | 0.990 | 0.405 | 0.246 | 0.000 | 0.291 | 0.105 | 0.051 |
| 21 | 0.946 | 0.640 | 0.083 | 0.617 | 0.431 | 0.997 | 0.386 | 0.234 | 0.000 | 0.277 | 0.100 | 0.049 |
| 22 | 0.916 | 0.619 | 0.082 | 0.605 | 0.428 | 0.982 | 0.368 | 0.224 | 0.000 | 0.265 | 0.095 | 0.046 |
| 23 | 0.887 | 0.605 | 0.081 | 0.595 | 0.423 | 0.973 | 0.352 | 0.214 | 0.000 | 0.253 | 0.091 | 0.044 |
| 24 | 0.858 | 0.588 | 0.078 | 0.580 | 0.415 | 0.960 | 0.338 | 0.205 | 0.000 | 0.243 | 0.088 | 0.043 |
| 25 | 0.833 | 0.571 | 0.074 | 0.566 | 0.408 | 0.941 | 0.324 | 0.197 | 0.000 | 0.233 | 0.084 | 0.041 |
| 26 | 0.808 | 0.556 | 0.076 | 0.554 | 0.399 | 0.923 | 0.312 | 0.189 | 0.000 | 0.224 | 0.081 | 0.039 |
| 27 | 0.784 | 0.542 | 0.076 | 0.538 | 0.391 | 0.907 | 0.300 | 0.182 | 0.000 | 0.216 | 0.078 | 0.038 |
| 28 | 0.763 | 0.529 | 0.073 | 0.525 | 0.384 | 0.887 | 0.289 | 0.176 | 0.000 | 0.208 | 0.075 | 0.036 |
| 29 | 0.741 | 0.513 | 0.070 | 0.513 | 0.377 | 0.867 | 0.279 | 0.170 | 0.000 | 0.201 | 0.072 | 0.035 |
| 30 | 0.720 | 0.500 | 0.070 | 0.500 | 0.368 | 0.850 | 0.270 | 0.164 | 0.000 | 0.194 | 0.070 | 0.034 |
| 31 | 0.701 | 0.488 | 0.070 | 0.488 | 0.360 | 0.830 | 0.261 | 0.159 | 0.019 | 0.188 | 0.068 | 0.033 |
| 32 | 0.683 | 0.476 | 0.071 | 0.478 | 0.353 | 0.812 | 0.253 | 0.154 | 0.009 | 0.182 | 0.066 | 0.032 |
| 33 | 0.665 | 0.464 | 0.073 | 0.465 | 0.345 | 0.796 | 0.245 | 0.149 | 0.009 | 0.176 | 0.064 | 0.031 |
| 34 | 0.649 | 0.454 | 0.071 | 0.455 | 0.337 | 0.778 | 0.238 | 0.145 | 0.009 | 0.171 | 0.062 | 0.030 |
| 35 | 0.634 | 0.441 | 0.077 | 0.446 | 0.333 | 0.761 | 0.231 | 0.141 | 0.009 | 0.166 | 0.060 | 0.031 |
| 36 | 0.618 | 0.430 | 0.075 | 0.435 | 0.325 | 0.745 | 0.225 | 0.137 | 0.008 | 0.162 | 0.058 | 0.030 |
| 37 | 0.605 | 0.422 | 0.073 | 0.426 | 0.319 | 0.730 | 0.219 | 0.133 | 0.008 | 0.157 | 0.057 | 0.029 |
| 38 | 0.591 | 0.412 | 0.076 | 0.417 | 0.313 | 0.715 | 0.213 | 0.129 | 0.008 | 0.153 | 0.055 | 0.028 |
| 39 | 0.578 | 0.403 | 0.077 | 0.408 | 0.308 | 0.702 | 0.208 | 0.126 | 0.008 | 0.149 | 0.054 | 0.028 |
| 40 | 0.566 | 0.393 | 0.077 | 0.401 | 0.302 | 0.687 | 0.203 | 0.123 | 0.008 | 0.146 | 0.053 | 0.027 |
| 41 | 0.555 | 0.386 | 0.079 | 0.392 | 0.296 | 0.673 | 0.198 | 0.120 | 0.007 | 0.142 | 0.051 | 0.026 |
| 42 | 0.543 | 0.377 | 0.079 | 0.384 | 0.290 | 0.661 | 0.193 | 0.117 | 0.007 | 0.139 | 0.050 | 0.026 |
| 43 | 0.532 | 0.370 | 0.081 | 0.377 | 0.285 | 0.649 | 0.188 | 0.114 | 0.007 | 0.135 | 0.049 | 0.025 |
| 44 | 0.521 | 0.363 | 0.082 | 0.370 | 0.280 | 0.637 | 0.184 | 0.112 | 0.007 | 0.132 | 0.048 | 0.025 |
| 45 | 0.511 | 0.356 | 0.080 | 0.361 | 0.275 | 0.625 | 0.180 | 0.109 | 0.007 | 0.129 | 0.047 | 0.024 |
| 46 | 0.501 | 0.348 | 0.083 | 0.356 | 0.270 | 0.613 | 0.176 | 0.107 | 0.007 | 0.127 | 0.046 | 0.023 |
| 47 | 0.491 | 0.342 | 0.084 | 0.350 | 0.266 | 0.604 | 0.172 | 0.105 | 0.006 | 0.124 | 0.045 | 0.023 |
| 48 | 0.481 | 0.335 | 0.088 | 0.344 | 0.261 | 0.594 | 0.169 | 0.103 | 0.006 | 0.121 | 0.044 | 0.023 |
| 49 | 0.473 | 0.328 | 0.089 | 0.337 | 0.257 | 0.582 | 0.165 | 0.100 | 0.006 | 0.119 | 0.043 | 0.022 |
| 50 | 0.464 | 0.323 | 0.090 | 0.331 | 0.253 | 0.572 | 0.162 | 0.098 | 0.006 | 0.116 | 0.042 | 0.022 |
| 51 | 0.456 | 0.318 | 0.091 | 0.326 | 0.249 | 0.564 | 0.159 | 0.096 | 0.006 | 0.114 | 0.041 | 0.021 |
| 52 | 0.449 | 0.312 | 0.092 | 0.321 | 0.246 | 0.554 | 0.156 | 0.095 | 0.006 | 0.112 | 0.040 | 0.021 |
| 53 | 0.442 | 0.307 | 0.092 | 0.316 | 0.242 | 0.546 | 0.153 | 0.093 | 0.006 | 0.110 | 0.040 | 0.020 |
| 54 | 0.433 | 0.301 | 0.093 | 0.311 | 0.239 | 0.537 | 0.150 | 0.091 | 0.006 | 0.108 | 0.039 | 0.020 |
| 55 | 0.427 | 0.296 | 0.095 | 0.305 | 0.235 | 0.528 | 0.147 | 0.089 | 0.005 | 0.106 | 0.038 | 0.020 |
| 56 | 0.420 | 0.291 | 0.096 | 0.301 | 0.231 | 0.521 | 0.145 | 0.088 | 0.005 | 0.104 | 0.038 | 0.019 |
| 57 | 0.413 | 0.286 | 0.098 | 0.296 | 0.227 | 0.513 | 0.142 | 0.086 | 0.005 | 0.102 | 0.037 | 0.019 |
| 58 | 0.407 | 0.281 | 0.098 | 0.292 | 0.224 | 0.505 | 0.140 | 0.085 | 0.005 | 0.100 | 0.036 | 0.018 |
| 59 | 0.401 | 0.278 | 0.099 | 0.288 | 0.222 | 0.497 | 0.137 | 0.083 | 0.005 | 0.099 | 0.036 | 0.017 |
| 60 | 0.394 | 0.273 | 0.100 | 0.283 | 0.218 | 0.490 | 0.135 | 0.082 | 0.005 | 0.097 | 0.035 | 0.017 |

**Table S6.** Settling and floating velocity (m h⁻¹) over the elapsed time (min) in Grease Trap B.

| **Time (min)** |  |  | **Settling Velocity** | |  |  |  |  | **Floating Velocity** | |  |  |
| --- | --- | --- | --- | --- | --- | --- | --- | --- | --- | --- | --- | --- |
|  | **B-R1-**  **I-C1** | **B-R1-**  **I-C2** | **B-R1-**  **O-C1** | **B-R2-**  **I-C1** | **B-R2-**  **I-C2** | **B-R2-**  **O-C1** | **B-R1-**  **I-C1** | **B-R1-**  **I-C2** | **B-R1-**  **O-C1** | **B-R2-**  **I-C1** | **B-R2-**  **I-C2** | **B-R2-**  **O-C1** |
| 0 | -- | -- | -- | -- | -- | -- | -- | -- | -- | -- | -- | -- |
| 1 | 0.000 | 0.000 | 0.000 | 0.000 | 0.000 | 0.000 | 0.000 | 0.000 | 0.000 | 0.000 | 0.000 | 0.000 |
| 2 | 0.600 | 1.500 | 0.000 | 0.000 | 0.000 | 0.000 | 1.050 | 0.300 | 0.000 | 0.000 | 0.000 | 0.000 |
| 3 | 1.400 | 1.000 | 0.000 | 0.760 | 0.700 | 0.000 | 0.760 | 0.200 | 0.000 | 0.760 | 0.700 | 0.000 |
| 4 | 1.350 | 0.900 | 0.000 | 0.720 | 0.675 | 0.000 | 0.585 | 0.180 | 0.000 | 0.720 | 0.675 | 0.000 |
| 5 | 1.320 | 0.960 | 0.000 | 0.732 | 0.660 | 0.360 | 0.480 | 0.156 | 0.000 | 0.732 | 0.660 | 0.300 |
| 6 | 1.400 | 1.000 | 0.000 | 0.710 | 0.750 | 0.300 | 0.400 | 0.130 | 0.000 | 0.710 | 0.550 | 0.250 |
| 7 | 1.371 | 1.029 | 0.000 | 0.711 | 0.557 | 0.369 | 0.343 | 0.111 | 0.000 | 0.711 | 0.471 | 0.214 |
| 8 | 1.350 | 1.125 | 0.000 | 0.660 | 0.525 | 0.330 | 0.308 | 0.097 | 0.000 | 0.660 | 0.413 | 0.158 |
| 9 | 1.400 | 1.133 | 0.000 | 0.787 | 0.467 | 0.300 | 0.273 | 0.087 | 0.000 | 0.687 | 0.367 | 0.147 |
| 10 | 1.380 | 1.140 | 0.000 | 0.738 | 0.450 | 0.300 | 0.246 | 0.084 | 0.000 | 0.618 | 0.330 | 0.132 |
| 11 | 1.391 | 1.145 | 0.000 | 0.671 | 0.409 | 0.300 | 0.224 | 0.076 | 0.000 | 0.551 | 0.300 | 0.120 |
| 12 | 1.375 | 1.150 | 0.550 | 0.630 | 0.390 | 0.315 | 0.205 | 0.070 | 0.600 | 0.500 | 0.275 | 0.110 |
| 13 | 1.362 | 1.131 | 0.554 | 0.591 | 0.369 | 0.323 | 0.189 | 0.069 | 0.415 | 0.457 | 0.254 | 0.102 |
| 14 | 1.346 | 1.157 | 0.471 | 0.561 | 0.364 | 0.317 | 0.176 | 0.064 | 0.364 | 0.420 | 0.236 | 0.094 |
| 15 | 1.336 | 1.136 | 0.440 | 0.532 | 0.372 | 0.312 | 0.164 | 0.060 | 0.340 | 0.392 | 0.220 | 0.088 |
| 16 | 1.313 | 1.125 | 0.413 | 0.503 | 0.360 | 0.300 | 0.154 | 0.056 | 0.319 | 0.368 | 0.206 | 0.083 |
| 17 | 1.299 | 1.119 | 0.388 | 0.480 | 0.353 | 0.307 | 0.145 | 0.053 | 0.300 | 0.346 | 0.194 | 0.078 |
| 18 | 1.273 | 1.113 | 0.383 | 0.460 | 0.350 | 0.310 | 0.137 | 0.050 | 0.283 | 0.327 | 0.183 | 0.073 |
| 19 | 1.247 | 1.096 | 0.379 | 0.439 | 0.347 | 0.316 | 0.129 | 0.047 | 0.268 | 0.309 | 0.174 | 0.069 |
| 20 | 1.230 | 1.080 | 0.375 | 0.423 | 0.336 | 0.315 | 0.123 | 0.045 | 0.255 | 0.294 | 0.165 | 0.066 |
| 21 | 1.200 | 1.046 | 0.371 | 0.409 | 0.329 | 0.314 | 0.117 | 0.043 | 0.243 | 0.266 | 0.146 | 0.063 |
| 22 | 1.173 | 1.025 | 0.368 | 0.393 | 0.314 | 0.325 | 0.112 | 0.041 | 0.232 | 0.254 | 0.139 | 0.060 |
| 23 | 1.148 | 1.004 | 0.357 | 0.378 | 0.300 | 0.331 | 0.107 | 0.039 | 0.222 | 0.243 | 0.133 | 0.057 |
| 24 | 1.113 | 0.975 | 0.350 | 0.370 | 0.288 | 0.325 | 0.103 | 0.038 | 0.225 | 0.233 | 0.128 | 0.055 |
| 25 | 1.085 | 0.950 | 0.348 | 0.355 | 0.276 | 0.324 | 0.098 | 0.036 | 0.216 | 0.223 | 0.122 | 0.053 |
| 26 | 1.057 | 0.925 | 0.346 | 0.346 | 0.268 | 0.330 | 0.095 | 0.035 | 0.208 | 0.215 | 0.118 | 0.051 |
| 27 | 1.031 | 0.902 | 0.344 | 0.351 | 0.262 | 0.333 | 0.091 | 0.033 | 0.200 | 0.207 | 0.113 | 0.049 |
| 28 | 1.007 | 0.879 | 0.343 | 0.381 | 0.253 | 0.332 | 0.088 | 0.032 | 0.191 | 0.199 | 0.109 | 0.047 |
| 29 | 0.981 | 0.857 | 0.341 | 0.385 | 0.248 | 0.331 | 0.085 | 0.031 | 0.182 | 0.192 | 0.106 | 0.046 |
| 30 | 0.956 | 0.834 | 0.340 | 0.380 | 0.240 | 0.330 | 0.082 | 0.030 | 0.174 | 0.186 | 0.102 | 0.044 |
| 31 | 0.935 | 0.813 | 0.331 | 0.377 | 0.234 | 0.333 | 0.079 | 0.029 | 0.168 | 0.180 | 0.099 | 0.043 |
| 32 | 0.911 | 0.791 | 0.332 | 0.371 | 0.229 | 0.334 | 0.077 | 0.028 | 0.161 | 0.174 | 0.096 | 0.041 |
| 33 | 0.891 | 0.775 | 0.329 | 0.369 | 0.224 | 0.336 | 0.075 | 0.027 | 0.155 | 0.169 | 0.093 | 0.040 |
| 34 | 0.870 | 0.759 | 0.326 | 0.362 | 0.221 | 0.335 | 0.072 | 0.026 | 0.150 | 0.164 | 0.090 | 0.039 |
| 35 | 0.852 | 0.741 | 0.326 | 0.357 | 0.214 | 0.334 | 0.070 | 0.026 | 0.146 | 0.159 | 0.087 | 0.038 |
| 36 | 0.833 | 0.725 | 0.325 | 0.350 | 0.208 | 0.332 | 0.068 | 0.025 | 0.142 | 0.155 | 0.085 | 0.037 |
| 37 | 0.817 | 0.710 | 0.324 | 0.344 | 0.204 | 0.341 | 0.066 | 0.024 | 0.138 | 0.151 | 0.083 | 0.036 |
| 38 | 0.801 | 0.695 | 0.324 | 0.338 | 0.199 | 0.339 | 0.065 | 0.024 | 0.134 | 0.147 | 0.081 | 0.035 |
| 39 | 0.785 | 0.678 | 0.325 | 0.334 | 0.194 | 0.338 | 0.063 | 0.023 | 0.131 | 0.143 | 0.078 | 0.034 |
| 40 | 0.770 | 0.666 | 0.326 | 0.327 | 0.191 | 0.338 | 0.061 | 0.023 | 0.128 | 0.138 | 0.075 | 0.033 |
| 41 | 0.754 | 0.653 | 0.326 | 0.319 | 0.186 | 0.337 | 0.060 | 0.022 | 0.124 | 0.135 | 0.073 | 0.032 |
| 42 | 0.740 | 0.640 | 0.329 | 0.316 | 0.183 | 0.334 | 0.059 | 0.021 | 0.121 | 0.130 | 0.071 | 0.031 |
| 43 | 0.726 | 0.628 | 0.329 | 0.311 | 0.179 | 0.331 | 0.057 | 0.021 | 0.119 | 0.127 | 0.070 | 0.031 |
| 44 | 0.713 | 0.618 | 0.330 | 0.305 | 0.175 | 0.327 | 0.056 | 0.020 | 0.116 | 0.124 | 0.068 | 0.030 |
| 45 | 0.700 | 0.607 | 0.329 | 0.301 | 0.172 | 0.324 | 0.055 | 0.020 | 0.113 | 0.121 | 0.067 | 0.029 |
| 46 | 0.687 | 0.595 | 0.329 | 0.297 | 0.168 | 0.321 | 0.053 | 0.020 | 0.111 | 0.119 | 0.065 | 0.029 |
| 47 | 0.677 | 0.585 | 0.329 | 0.291 | 0.165 | 0.319 | 0.052 | 0.019 | 0.109 | 0.116 | 0.064 | 0.028 |
| 48 | 0.664 | 0.575 | 0.328 | 0.286 | 0.163 | 0.315 | 0.051 | 0.019 | 0.106 | 0.114 | 0.063 | 0.028 |
| 49 | 0.651 | 0.563 | 0.327 | 0.283 | 0.159 | 0.311 | 0.050 | 0.018 | 0.104 | 0.111 | 0.061 | 0.027 |
| 50 | 0.642 | 0.556 | 0.325 | 0.278 | 0.160 | 0.308 | 0.049 | 0.018 | 0.102 | 0.109 | 0.060 | 0.026 |
| 51 | 0.631 | 0.547 | 0.324 | 0.274 | 0.156 | 0.306 | 0.048 | 0.018 | 0.100 | 0.107 | 0.059 | 0.026 |
| 52 | 0.622 | 0.538 | 0.321 | 0.269 | 0.153 | 0.302 | 0.047 | 0.017 | 0.098 | 0.105 | 0.058 | 0.025 |
| 53 | 0.611 | 0.530 | 0.318 | 0.266 | 0.151 | 0.299 | 0.046 | 0.017 | 0.096 | 0.103 | 0.057 | 0.025 |
| 54 | 0.601 | 0.521 | 0.317 | 0.262 | 0.149 | 0.296 | 0.046 | 0.017 | 0.094 | 0.101 | 0.056 | 0.024 |
| 55 | 0.592 | 0.513 | 0.315 | 0.259 | 0.146 | 0.295 | 0.045 | 0.016 | 0.093 | 0.099 | 0.055 | 0.024 |
| 56 | 0.584 | 0.505 | 0.312 | 0.255 | 0.144 | 0.290 | 0.044 | 0.016 | 0.091 | 0.097 | 0.054 | 0.024 |
| 57 | 0.575 | 0.499 | 0.311 | 0.252 | 0.142 | 0.287 | 0.043 | 0.016 | 0.089 | 0.096 | 0.053 | 0.023 |
| 58 | 0.566 | 0.491 | 0.310 | 0.247 | 0.140 | 0.284 | 0.042 | 0.016 | 0.088 | 0.094 | 0.052 | 0.023 |
| 59 | 0.558 | 0.484 | 0.307 | 0.245 | 0.137 | 0.281 | 0.042 | 0.015 | 0.086 | 0.093 | 0.051 | 0.022 |
| 60 | 0.550 | 0.477 | 0.305 | 0.242 | 0.135 | 0.278 | 0.041 | 0.015 | 0.085 | 0.091 | 0.050 | 0.022 |

**Table S7.** Settling and floating velocity (m h⁻¹) over the elapsed time (min) in Grease Trap C.

| **Time (min)** |  |  | **Settling Velocity** | |  |  |  |  | **Floating Velocity** | |  |  |
| --- | --- | --- | --- | --- | --- | --- | --- | --- | --- | --- | --- | --- |
|  | **C-R1-**  **I-C1** | **C-R1-**  **I-C2** | **C-R1-**  **O-C1** | **C-R2-**  **I-C1** | **C-R2-**  **I-C2** | **C-R2-**  **O-C1** | **C-R1-**  **I-C1** | **C-R1-**  **I-C2** | **C-R1-**  **O-C1** | **C-R2-**  **I-C1** | **C-R2-**  **I-C2** | **C-R2-**  **O-C1** |
| 0 | -- | -- | -- | -- | -- | -- | -- | -- | -- | -- | -- | -- |
| 1 | 5.400 | 0.660 | 0.600 | 1.440 | 1.440 | 0.000 | 1.260 | 0.660 | 0.120 | 1.440 | 1.440 | 0.240 |
| 2 | 3.600 | 0.810 | 1.350 | 1.020 | 1.020 | 2.400 | 0.750 | 0.360 | 0.060 | 1.020 | 1.020 | 0.150 |
| 3 | 4.000 | 1.740 | 1.700 | 0.800 | 0.800 | 2.600 | 0.520 | 0.280 | 0.040 | 0.800 | 0.800 | 0.120 |
| 4 | 3.450 | 2.505 | 1.950 | 0.660 | 0.660 | 3.000 | 0.390 | 0.225 | 0.030 | 0.660 | 0.660 | 0.090 |
| 5 | 3.600 | 3.264 | 2.040 | 2.028 | 2.088 | 3.480 | 0.336 | 0.180 | 0.024 | 0.564 | 0.540 | 0.084 |
| 6 | 3.400 | 3.170 | 2.200 | 2.440 | 2.240 | 3.500 | 0.280 | 0.150 | 0.020 | 0.490 | 0.460 | 0.070 |
| 7 | 3.429 | 3.403 | 2.057 | 3.034 | 3.034 | 4.029 | 0.240 | 0.137 | 0.017 | 0.420 | 0.394 | 0.060 |
| 8 | 3.300 | 3.840 | 2.100 | 2.880 | 2.880 | 4.050 | 0.218 | 0.120 | 0.015 | 0.375 | 0.345 | 0.053 |
| 9 | 3.333 | 3.580 | 2.067 | 2.927 | 3.093 | 4.200 | 0.200 | 0.113 | 0.013 | 0.333 | 0.313 | 0.047 |
| 10 | 3.180 | 3.672 | 2.040 | 3.084 | 3.024 | 4.134 | 0.180 | 0.102 | 0.012 | 0.300 | 0.282 | 0.042 |
| 11 | 3.000 | 3.475 | 2.018 | 3.404 | 3.076 | 3.769 | 0.169 | 0.093 | 0.011 | 0.273 | 0.256 | 0.038 |
| 12 | 2.790 | 3.285 | 1.975 | 3.200 | 2.890 | 3.490 | 0.155 | 0.085 | 0.010 | 0.250 | 0.240 | 0.035 |
| 13 | 2.626 | 3.078 | 1.938 | 2.968 | 2.677 | 3.245 | 0.148 | 0.078 | 0.009 | 0.240 | 0.222 | 0.032 |
| 14 | 2.464 | 2.901 | 1.864 | 2.764 | 2.507 | 3.039 | 0.137 | 0.077 | 0.009 | 0.223 | 0.206 | 0.030 |
| 15 | 2.320 | 2.728 | 1.820 | 2.588 | 2.352 | 2.848 | 0.128 | 0.072 | 0.008 | 0.204 | 0.192 | 0.028 |
| 16 | 2.194 | 2.576 | 1.744 | 2.441 | 2.220 | 2.681 | 0.124 | 0.067 | 0.008 | 0.191 | 0.176 | 0.026 |
| 17 | 2.082 | 2.442 | 1.694 | 2.308 | 2.100 | 2.531 | 0.116 | 0.067 | 0.007 | 0.180 | 0.169 | 0.025 |
| 18 | 1.983 | 2.323 | 1.640 | 2.183 | 1.993 | 2.400 | 0.110 | 0.063 | 0.007 | 0.170 | 0.160 | 0.023 |
| 19 | 1.895 | 2.214 | 1.579 | 2.078 | 1.895 | 2.283 | 0.104 | 0.060 | 0.006 | 0.161 | 0.152 | 0.022 |
| 20 | 1.809 | 2.112 | 1.530 | 1.977 | 1.806 | 2.175 | 0.099 | 0.057 | 0.006 | 0.153 | 0.144 | 0.021 |
| 21 | 1.729 | 2.020 | 1.486 | 1.889 | 1.729 | 2.077 | 0.094 | 0.054 | 0.006 | 0.146 | 0.137 | 0.020 |
| 22 | 1.661 | 1.939 | 1.432 | 1.805 | 1.653 | 1.985 | 0.090 | 0.052 | 0.005 | 0.139 | 0.131 | 0.019 |
| 23 | 1.594 | 1.860 | 1.388 | 1.730 | 1.586 | 1.904 | 0.086 | 0.050 | 0.005 | 0.133 | 0.125 | 0.018 |
| 24 | 1.535 | 1.790 | 1.345 | 1.663 | 1.525 | 1.830 | 0.082 | 0.048 | 0.005 | 0.128 | 0.120 | 0.018 |
| 25 | 1.478 | 1.723 | 1.303 | 1.598 | 1.466 | 1.759 | 0.079 | 0.046 | 0.005 | 0.122 | 0.115 | 0.017 |
| 26 | 1.426 | 1.662 | 1.265 | 1.539 | 1.412 | 1.696 | 0.076 | 0.044 | 0.005 | 0.118 | 0.111 | 0.016 |
| 27 | 1.378 | 1.604 | 1.227 | 1.484 | 1.362 | 1.636 | 0.073 | 0.042 | 0.004 | 0.116 | 0.107 | 0.016 |
| 28 | 1.333 | 1.551 | 1.189 | 1.434 | 1.316 | 1.579 | 0.071 | 0.041 | 0.004 | 0.111 | 0.103 | 0.015 |
| 29 | 1.291 | 1.502 | 1.159 | 1.384 | 1.272 | 1.527 | 0.068 | 0.039 | 0.004 | 0.108 | 0.099 | 0.014 |
| 30 | 1.252 | 1.454 | 1.126 | 1.340 | 1.232 | 1.478 | 0.066 | 0.040 | 0.004 | 0.104 | 0.096 | 0.014 |
| 31 | 1.214 | 1.409 | 1.095 | 1.299 | 1.194 | 1.432 | 0.064 | 0.039 | 0.004 | 0.101 | 0.093 | 0.014 |
| 32 | 1.178 | 1.369 | 1.069 | 1.260 | 1.159 | 1.389 | 0.062 | 0.038 | 0.004 | 0.098 | 0.090 | 0.013 |
| 33 | 1.144 | 1.329 | 1.042 | 1.222 | 1.125 | 1.347 | 0.060 | 0.036 | 0.004 | 0.095 | 0.087 | 0.013 |
| 34 | 1.112 | 1.292 | 1.015 | 1.188 | 1.094 | 1.309 | 0.058 | 0.035 | 0.004 | 0.092 | 0.085 | 0.012 |
| 35 | 1.083 | 1.257 | 0.991 | 1.155 | 1.063 | 1.274 | 0.057 | 0.034 | 0.003 | 0.089 | 0.082 | 0.012 |
| 36 | 1.055 | 1.225 | 0.967 | 1.125 | 1.035 | 1.240 | 0.055 | 0.033 | 0.003 | 0.087 | 0.080 | 0.012 |
| 37 | 1.028 | 1.194 | 0.947 | 1.095 | 1.007 | 1.206 | 0.054 | 0.032 | 0.003 | 0.084 | 0.078 | 0.011 |
| 38 | 1.003 | 1.162 | 0.925 | 1.066 | 0.981 | 1.176 | 0.052 | 0.032 | 0.003 | 0.082 | 0.076 | 0.011 |
| 39 | 0.978 | 1.134 | 0.905 | 1.038 | 0.957 | 1.148 | 0.051 | 0.031 | 0.003 | 0.080 | 0.074 | 0.011 |
| 40 | 0.956 | 1.106 | 0.885 | 1.014 | 0.933 | 1.119 | 0.050 | 0.030 | 0.003 | 0.080 | 0.072 | 0.011 |
| 41 | 0.934 | 1.080 | 0.866 | 0.989 | 0.913 | 1.093 | 0.048 | 0.029 | 0.003 | 0.078 | 0.070 | 0.010 |
| 42 | 0.913 | 1.056 | 0.849 | 0.967 | 0.891 | 1.067 | 0.047 | 0.029 | 0.003 | 0.076 | 0.070 | 0.010 |
| 43 | 0.893 | 1.033 | 0.830 | 0.946 | 0.871 | 1.044 | 0.046 | 0.028 | 0.003 | 0.074 | 0.068 | 0.010 |
| 44 | 0.873 | 1.010 | 0.813 | 0.925 | 0.852 | 1.020 | 0.045 | 0.027 | 0.003 | 0.072 | 0.067 | 0.010 |
| 45 | 0.855 | 0.989 | 0.800 | 0.904 | 0.833 | 0.999 | 0.044 | 0.027 | 0.003 | 0.072 | 0.065 | 0.009 |
| 46 | 0.836 | 0.968 | 0.785 | 0.886 | 0.815 | 0.977 | 0.043 | 0.026 | 0.003 | 0.070 | 0.064 | 0.009 |
| 47 | 0.820 | 0.949 | 0.770 | 0.867 | 0.798 | 0.957 | 0.042 | 0.026 | 0.003 | 0.069 | 0.063 | 0.009 |
| 48 | 0.804 | 0.929 | 0.756 | 0.850 | 0.783 | 0.938 | 0.041 | 0.025 | 0.003 | 0.068 | 0.061 | 0.009 |
| 49 | 0.787 | 0.911 | 0.742 | 0.833 | 0.767 | 0.920 | 0.040 | 0.024 | 0.002 | 0.066 | 0.060 | 0.009 |
| 50 | 0.773 | 0.893 | 0.728 | 0.816 | 0.752 | 0.901 | 0.040 | 0.024 | 0.002 | 0.065 | 0.059 | 0.008 |
| 51 | 0.759 | 0.875 | 0.718 | 0.800 | 0.738 | 0.884 | 0.039 | 0.024 | 0.002 | 0.064 | 0.058 | 0.008 |
| 52 | 0.744 | 0.861 | 0.705 | 0.785 | 0.723 | 0.868 | 0.038 | 0.023 | 0.002 | 0.062 | 0.057 | 0.008 |
| 53 | 0.730 | 0.845 | 0.693 | 0.770 | 0.711 | 0.851 | 0.037 | 0.023 | 0.002 | 0.061 | 0.055 | 0.008 |
| 54 | 0.718 | 0.830 | 0.681 | 0.756 | 0.698 | 0.836 | 0.037 | 0.022 | 0.002 | 0.060 | 0.054 | 0.008 |
| 55 | 0.705 | 0.815 | 0.671 | 0.743 | 0.685 | 0.821 | 0.036 | 0.022 | 0.002 | 0.059 | 0.053 | 0.008 |
| 56 | 0.693 | 0.800 | 0.660 | 0.730 | 0.674 | 0.807 | 0.035 | 0.021 | 0.002 | 0.058 | 0.053 | 0.008 |
| 57 | 0.681 | 0.787 | 0.649 | 0.717 | 0.662 | 0.793 | 0.035 | 0.021 | 0.002 | 0.057 | 0.052 | 0.007 |
| 58 | 0.670 | 0.774 | 0.639 | 0.704 | 0.651 | 0.780 | 0.034 | 0.021 | 0.002 | 0.056 | 0.051 | 0.007 |
| 59 | 0.659 | 0.762 | 0.629 | 0.694 | 0.640 | 0.767 | 0.034 | 0.020 | 0.002 | 0.055 | 0.050 | 0.007 |
| 60 | 0.649 | 0.749 | 0.620 | 0.682 | 0.629 | 0.754 | 0.033 | 0.020 | 0.002 | 0.054 | 0.049 | 0.007 |

**Table S8.** Settling and floating velocity (m h⁻¹) over the elapsed time (min) in Grease Trap D.

| **Time (min)** |  |  | **Settling Velocity** | |  |  |  |  | **Floating Velocity** | |  |  |
| --- | --- | --- | --- | --- | --- | --- | --- | --- | --- | --- | --- | --- |
|  | **D-R1-**  **I-C1** | **D-R1-**  **I-C2** | **D-R1-**  **O-C1** | **D-R2-**  **I-C1** | **D-R2-**  **I-C2** | **D-R2-**  **O-C1** | **D-R1-**  **I-C1** | **D-R1-**  **I-C2** | **D-R1-**  **O-C1** | **D-R2-**  **I-C1** | **D-R2-**  **I-C2** | **D-R2-**  **O-C1** |
| 0 | -- | -- | -- | -- | -- | -- | -- | -- | -- | -- | -- | -- |
| 1 | 1.800 | 1.800 | 0.000 | 3.600 | 2.700 | 0.600 | 0.300 | 0.420 | 0.300 | 2.400 | 1.740 | 0.600 |
| 2 | 1.350 | 1.350 | 0.000 | 2.100 | 1.650 | 2.100 | 0.300 | 0.270 | 0.210 | 1.500 | 1.050 | 0.330 |
| 3 | 1.200 | 1.200 | 3.200 | 2.400 | 2.200 | 3.400 | 0.220 | 0.200 | 0.140 | 1.100 | 0.760 | 0.220 |
| 4 | 1.650 | 1.650 | 3.900 | 2.850 | 2.850 | 3.600 | 0.165 | 0.165 | 0.105 | 0.855 | 0.600 | 0.195 |
| 5 | 2.160 | 2.160 | 4.080 | 3.600 | 3.600 | 4.320 | 0.144 | 0.144 | 0.084 | 0.708 | 0.480 | 0.180 |
| 6 | 2.300 | 2.300 | 4.500 | 4.500 | 4.200 | 4.000 | 0.120 | 0.120 | 0.070 | 0.610 | 0.420 | 0.150 |
| 7 | 2.229 | 2.229 | 4.629 | 4.286 | 4.286 | 3.857 | 0.111 | 0.111 | 0.060 | 0.531 | 0.360 | 0.129 |
| 8 | 2.325 | 2.325 | 4.725 | 4.950 | 4.575 | 3.750 | 0.105 | 0.097 | 0.060 | 0.458 | 0.315 | 0.120 |
| 9 | 2.333 | 2.333 | 4.600 | 4.867 | 4.533 | 3.800 | 0.100 | 0.087 | 0.053 | 0.407 | 0.280 | 0.107 |
| 10 | 2.460 | 2.460 | 4.440 | 4.560 | 4.170 | 4.668 | 0.090 | 0.084 | 0.048 | 0.366 | 0.252 | 0.096 |
| 11 | 2.455 | 2.455 | 4.075 | 4.151 | 3.807 | 4.255 | 0.082 | 0.076 | 0.044 | 0.344 | 0.235 | 0.087 |
| 12 | 2.375 | 2.400 | 3.770 | 3.835 | 3.515 | 3.915 | 0.075 | 0.070 | 0.040 | 0.320 | 0.215 | 0.080 |
| 13 | 2.317 | 2.331 | 3.503 | 3.545 | 3.249 | 3.628 | 0.069 | 0.065 | 0.037 | 0.295 | 0.198 | 0.074 |
| 14 | 2.207 | 2.220 | 3.266 | 3.304 | 3.034 | 3.381 | 0.064 | 0.060 | 0.034 | 0.274 | 0.184 | 0.069 |
| 15 | 2.120 | 2.128 | 3.064 | 3.092 | 2.840 | 3.164 | 0.060 | 0.056 | 0.032 | 0.256 | 0.176 | 0.064 |
| 16 | 2.036 | 2.025 | 2.884 | 2.910 | 2.678 | 2.974 | 0.056 | 0.053 | 0.030 | 0.240 | 0.165 | 0.060 |
| 17 | 1.945 | 1.938 | 2.725 | 2.742 | 2.527 | 2.806 | 0.053 | 0.049 | 0.028 | 0.226 | 0.155 | 0.056 |
| 18 | 1.867 | 1.850 | 2.580 | 2.597 | 2.393 | 2.657 | 0.050 | 0.047 | 0.027 | 0.213 | 0.147 | 0.053 |
| 19 | 1.787 | 1.772 | 2.451 | 2.469 | 2.277 | 2.520 | 0.047 | 0.044 | 0.025 | 0.202 | 0.139 | 0.051 |
| 20 | 1.716 | 1.701 | 2.337 | 2.352 | 2.169 | 2.400 | 0.045 | 0.042 | 0.024 | 0.192 | 0.132 | 0.048 |
| 21 | 1.651 | 1.629 | 2.229 | 2.246 | 2.069 | 2.289 | 0.043 | 0.040 | 0.023 | 0.183 | 0.126 | 0.046 |
| 22 | 1.595 | 1.571 | 2.133 | 2.149 | 1.977 | 2.185 | 0.044 | 0.038 | 0.022 | 0.175 | 0.120 | 0.044 |
| 23 | 1.534 | 1.513 | 2.045 | 2.058 | 1.899 | 2.092 | 0.042 | 0.037 | 0.021 | 0.167 | 0.115 | 0.042 |
| 24 | 1.478 | 1.458 | 1.963 | 1.975 | 1.823 | 2.008 | 0.040 | 0.035 | 0.020 | 0.160 | 0.110 | 0.040 |
| 25 | 1.428 | 1.406 | 1.889 | 1.901 | 1.754 | 1.930 | 0.038 | 0.034 | 0.019 | 0.154 | 0.106 | 0.038 |
| 26 | 1.385 | 1.362 | 1.818 | 1.832 | 1.689 | 1.858 | 0.037 | 0.032 | 0.018 | 0.148 | 0.102 | 0.037 |
| 27 | 1.338 | 1.316 | 1.756 | 1.767 | 1.629 | 1.791 | 0.036 | 0.031 | 0.018 | 0.142 | 0.098 | 0.036 |
| 28 | 1.296 | 1.275 | 1.693 | 1.706 | 1.573 | 1.729 | 0.034 | 0.030 | 0.017 | 0.137 | 0.094 | 0.034 |
| 29 | 1.260 | 1.239 | 1.639 | 1.647 | 1.521 | 1.672 | 0.033 | 0.029 | 0.017 | 0.132 | 0.091 | 0.033 |
| 30 | 1.220 | 1.200 | 1.586 | 1.594 | 1.472 | 1.616 | 0.032 | 0.028 | 0.016 | 0.128 | 0.088 | 0.032 |
| 31 | 1.190 | 1.169 | 1.535 | 1.543 | 1.426 | 1.566 | 0.031 | 0.027 | 0.015 | 0.124 | 0.085 | 0.031 |
| 32 | 1.159 | 1.136 | 1.489 | 1.498 | 1.384 | 1.519 | 0.032 | 0.026 | 0.015 | 0.120 | 0.083 | 0.030 |
| 33 | 1.127 | 1.105 | 1.444 | 1.455 | 1.342 | 1.473 | 0.031 | 0.025 | 0.015 | 0.116 | 0.080 | 0.029 |
| 34 | 1.099 | 1.076 | 1.401 | 1.412 | 1.304 | 1.431 | 0.030 | 0.025 | 0.014 | 0.113 | 0.078 | 0.026 |
| 35 | 1.071 | 1.049 | 1.363 | 1.373 | 1.269 | 1.390 | 0.029 | 0.024 | 0.014 | 0.110 | 0.075 | 0.026 |
| 36 | 1.045 | 1.023 | 1.327 | 1.337 | 1.233 | 1.353 | 0.028 | 0.023 | 0.013 | 0.107 | 0.073 | 0.025 |
| 37 | 1.022 | 0.997 | 1.296 | 1.302 | 1.202 | 1.317 | 0.028 | 0.023 | 0.013 | 0.104 | 0.071 | 0.024 |
| 38 | 0.995 | 0.974 | 1.262 | 1.268 | 1.172 | 1.284 | 0.027 | 0.022 | 0.013 | 0.101 | 0.069 | 0.024 |
| 39 | 0.975 | 0.954 | 1.231 | 1.237 | 1.142 | 1.251 | 0.026 | 0.022 | 0.012 | 0.098 | 0.068 | 0.025 |
| 40 | 0.953 | 0.930 | 1.200 | 1.206 | 1.115 | 1.221 | 0.026 | 0.021 | 0.012 | 0.096 | 0.066 | 0.024 |
| 41 | 0.931 | 0.909 | 1.172 | 1.178 | 1.087 | 1.191 | 0.025 | 0.020 | 0.012 | 0.094 | 0.064 | 0.023 |
| 42 | 0.911 | 0.891 | 1.144 | 1.150 | 1.063 | 1.163 | 0.024 | 0.020 | 0.011 | 0.091 | 0.063 | 0.023 |
| 43 | 0.893 | 0.872 | 1.118 | 1.123 | 1.038 | 1.137 | 0.024 | 0.020 | 0.011 | 0.089 | 0.061 | 0.022 |
| 44 | 0.873 | 0.854 | 1.092 | 1.099 | 1.016 | 1.111 | 0.023 | 0.019 | 0.011 | 0.087 | 0.060 | 0.022 |
| 45 | 0.856 | 0.836 | 1.068 | 1.075 | 0.993 | 1.087 | 0.023 | 0.019 | 0.011 | 0.085 | 0.059 | 0.021 |
| 46 | 0.839 | 0.819 | 1.046 | 1.051 | 0.972 | 1.063 | 0.022 | 0.018 | 0.010 | 0.083 | 0.057 | 0.021 |
| 47 | 0.823 | 0.804 | 1.024 | 1.030 | 0.951 | 1.042 | 0.022 | 0.018 | 0.010 | 0.082 | 0.056 | 0.020 |
| 48 | 0.806 | 0.788 | 1.003 | 1.009 | 0.933 | 1.020 | 0.021 | 0.018 | 0.010 | 0.080 | 0.055 | 0.020 |
| 49 | 0.791 | 0.773 | 0.982 | 0.988 | 0.913 | 0.999 | 0.021 | 0.017 | 0.010 | 0.078 | 0.054 | 0.020 |
| 50 | 0.776 | 0.758 | 0.964 | 0.968 | 0.895 | 0.979 | 0.020 | 0.017 | 0.010 | 0.077 | 0.053 | 0.019 |
| 51 | 0.762 | 0.745 | 0.945 | 0.951 | 0.879 | 0.961 | 0.020 | 0.016 | 0.009 | 0.075 | 0.052 | 0.019 |
| 52 | 0.749 | 0.732 | 0.927 | 0.932 | 0.862 | 0.943 | 0.020 | 0.016 | 0.009 | 0.074 | 0.051 | 0.018 |
| 53 | 0.736 | 0.719 | 0.910 | 0.915 | 0.846 | 0.925 | 0.019 | 0.016 | 0.009 | 0.072 | 0.050 | 0.018 |
| 54 | 0.723 | 0.707 | 0.893 | 0.899 | 0.830 | 0.908 | 0.019 | 0.016 | 0.009 | 0.071 | 0.049 | 0.018 |
| 55 | 0.711 | 0.694 | 0.877 | 0.883 | 0.815 | 0.891 | 0.019 | 0.015 | 0.009 | 0.070 | 0.048 | 0.017 |
| 56 | 0.700 | 0.683 | 0.861 | 0.867 | 0.801 | 0.876 | 0.018 | 0.015 | 0.009 | 0.069 | 0.047 | 0.017 |
| 57 | 0.688 | 0.672 | 0.847 | 0.852 | 0.787 | 0.861 | 0.018 | 0.015 | 0.008 | 0.067 | 0.046 | 0.017 |
| 58 | 0.678 | 0.661 | 0.833 | 0.837 | 0.774 | 0.846 | 0.018 | 0.014 | 0.008 | 0.066 | 0.046 | 0.017 |
| 59 | 0.667 | 0.651 | 0.819 | 0.823 | 0.761 | 0.832 | 0.017 | 0.014 | 0.008 | 0.065 | 0.045 | 0.016 |
| 60 | 0.657 | 0.641 | 0.805 | 0.809 | 0.748 | 0.818 | 0.017 | 0.014 | 0.008 | 0.064 | 0.044 | 0.016 |

**Table S9.** Photographs of column tests (CTs) for Round 1 (R1) samples collected from Grease Traps A, including Column 1 (C1) for both inlet (I) and outlet (O) chamber samples and Column 2 (C2) for inlet (I) chamber samples only.


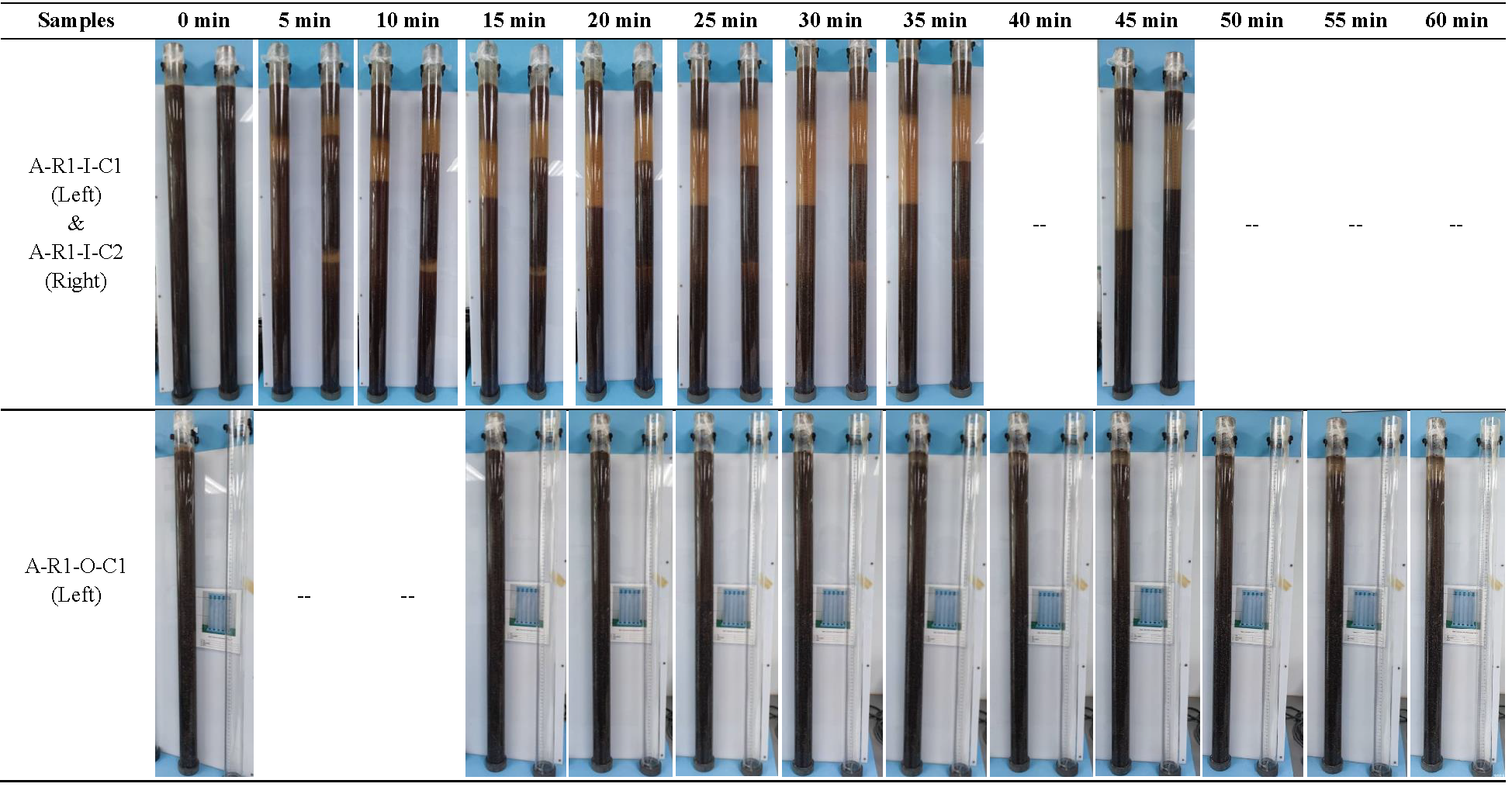


**Table S10.** Photographs of column tests (CTs) for Round 2 (R2) samples collected from Grease Traps A, including Column 1 (C1) for both inlet (I) and outlet (O) chamber samples and Column 2 (C2) for inlet (I) chamber samples only.


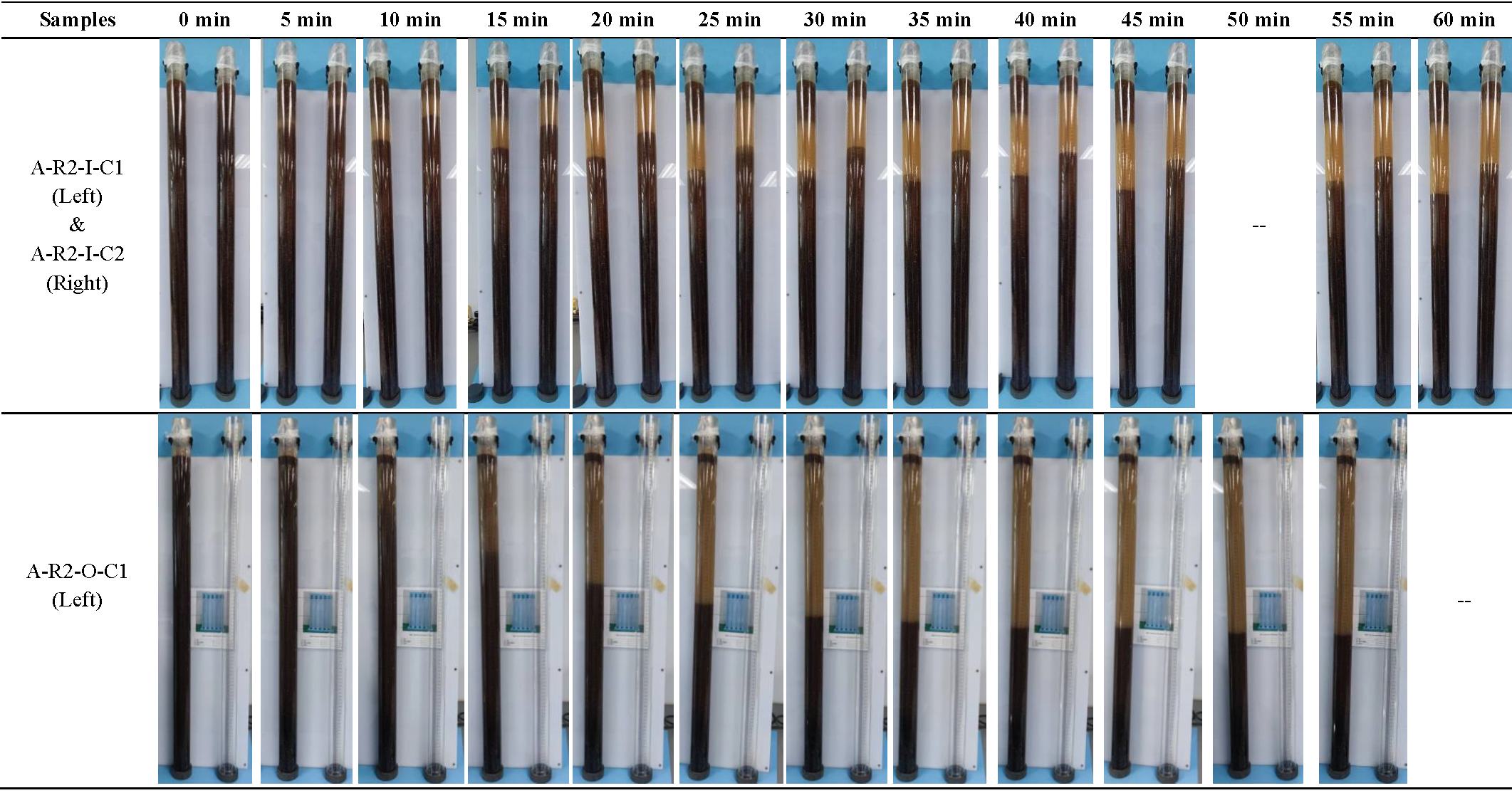


**Table S11.** Photographs of column tests (CTs) for Round 1 (R1) samples collected from Grease Traps B, including Column 1 (C1) for both inlet (I) and outlet (O) chamber samples and Column 2 (C2) for inlet (I) chamber samples only.


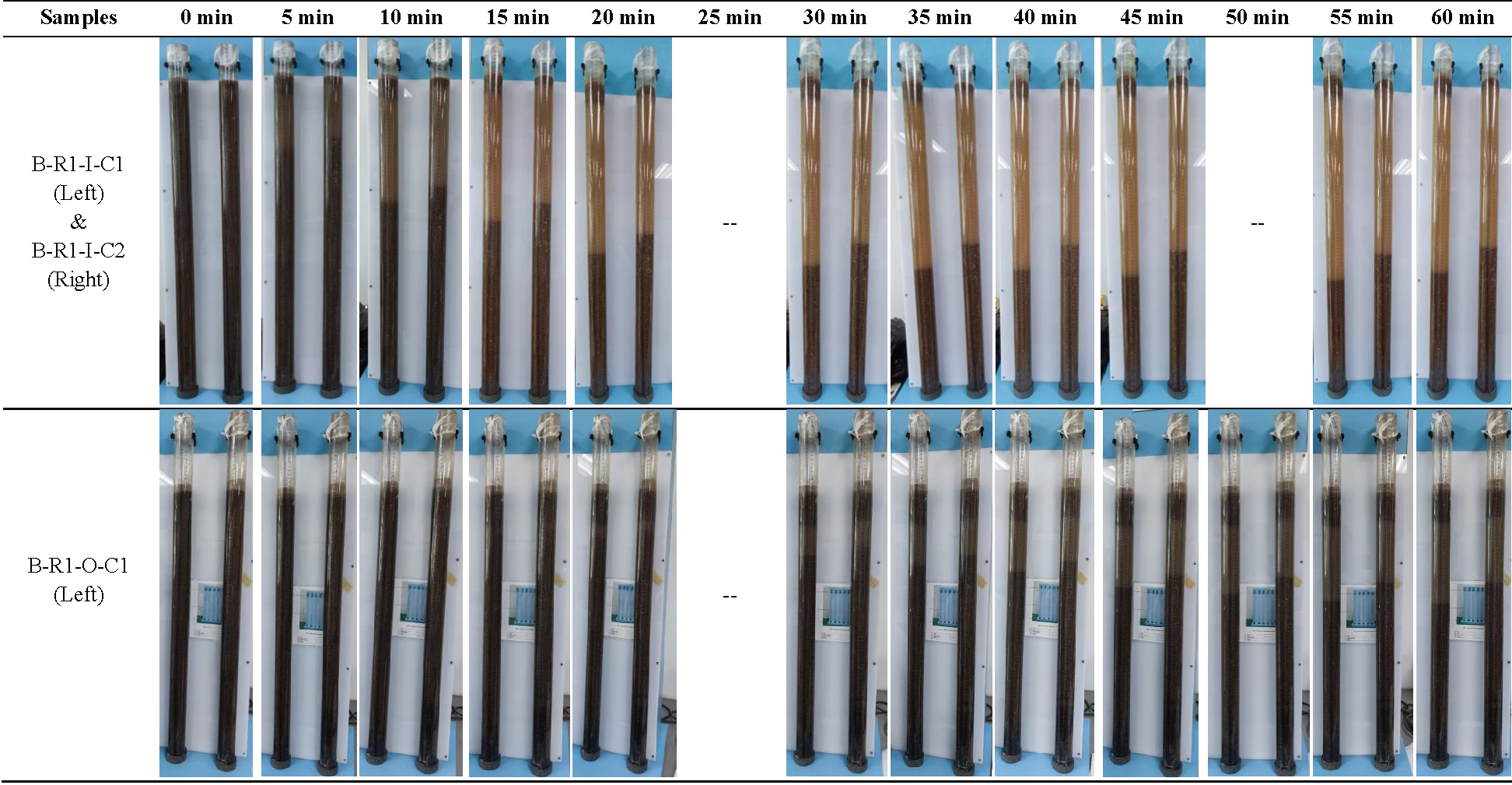


**Table S12.** Photographs of column tests (CTs) for Round 2 (R1) samples collected from Grease Traps B including Column 1 (C1) for both inlet (I) and outlet (O) chamber samples and Column 2 (C2) for inlet (I) chamber samples only.


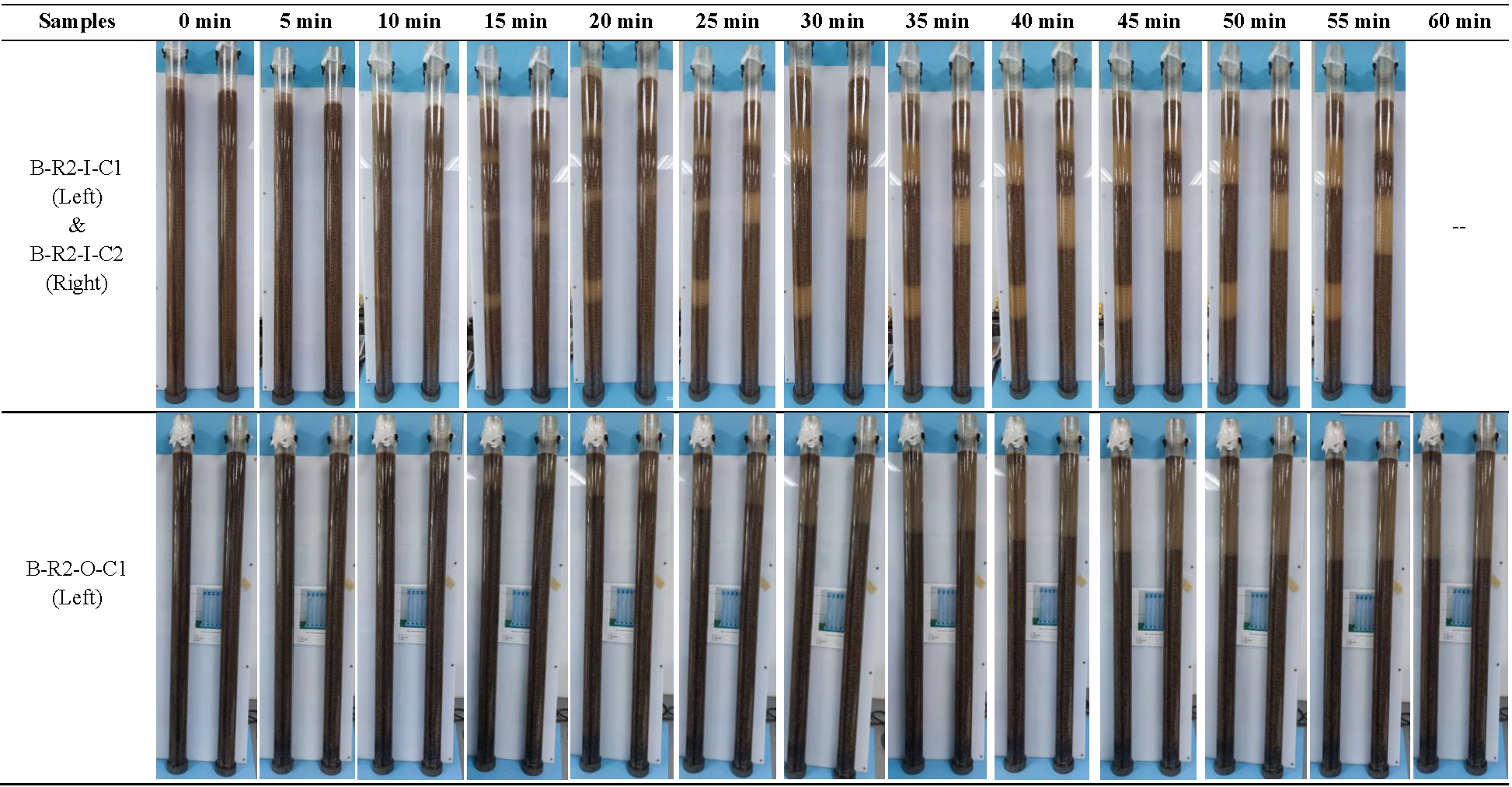


**Table S13.** Photographs of column tests (CTs) for Round 1 (R1) samples collected from Grease Traps C, including Column 1 (C1) for both inlet (I) and outlet (O) chamber samples and Column 2 (C2) for inlet (I) chamber samples only.


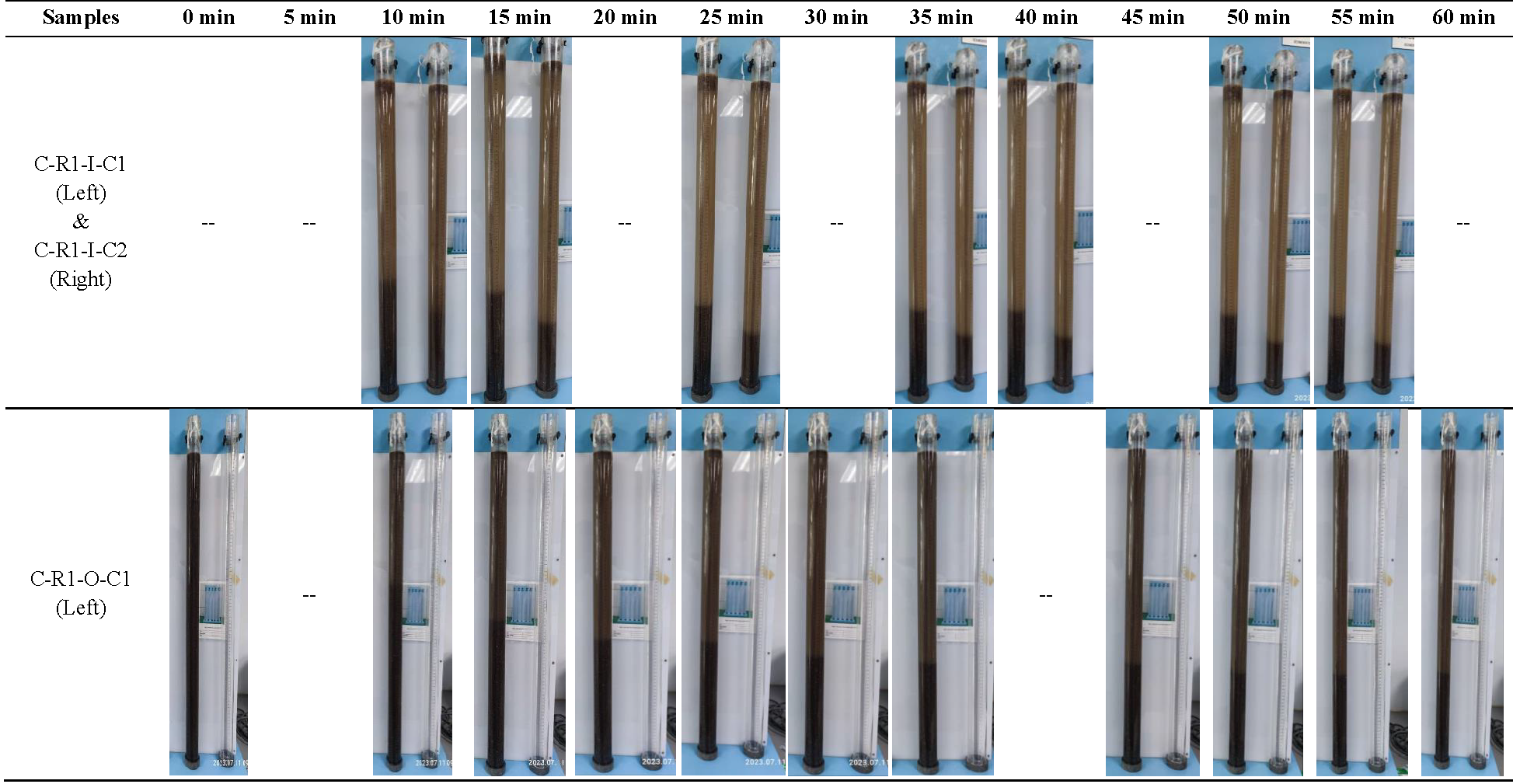


**Table S14.** Photographs of column tests (CTs) for Round 2 (R2) samples collected from Grease Traps C, including Column 1 (C1) for both inlet (I) and outlet (O) chamber samples and Column 2 (C2) for inlet (I) chamber samples only.


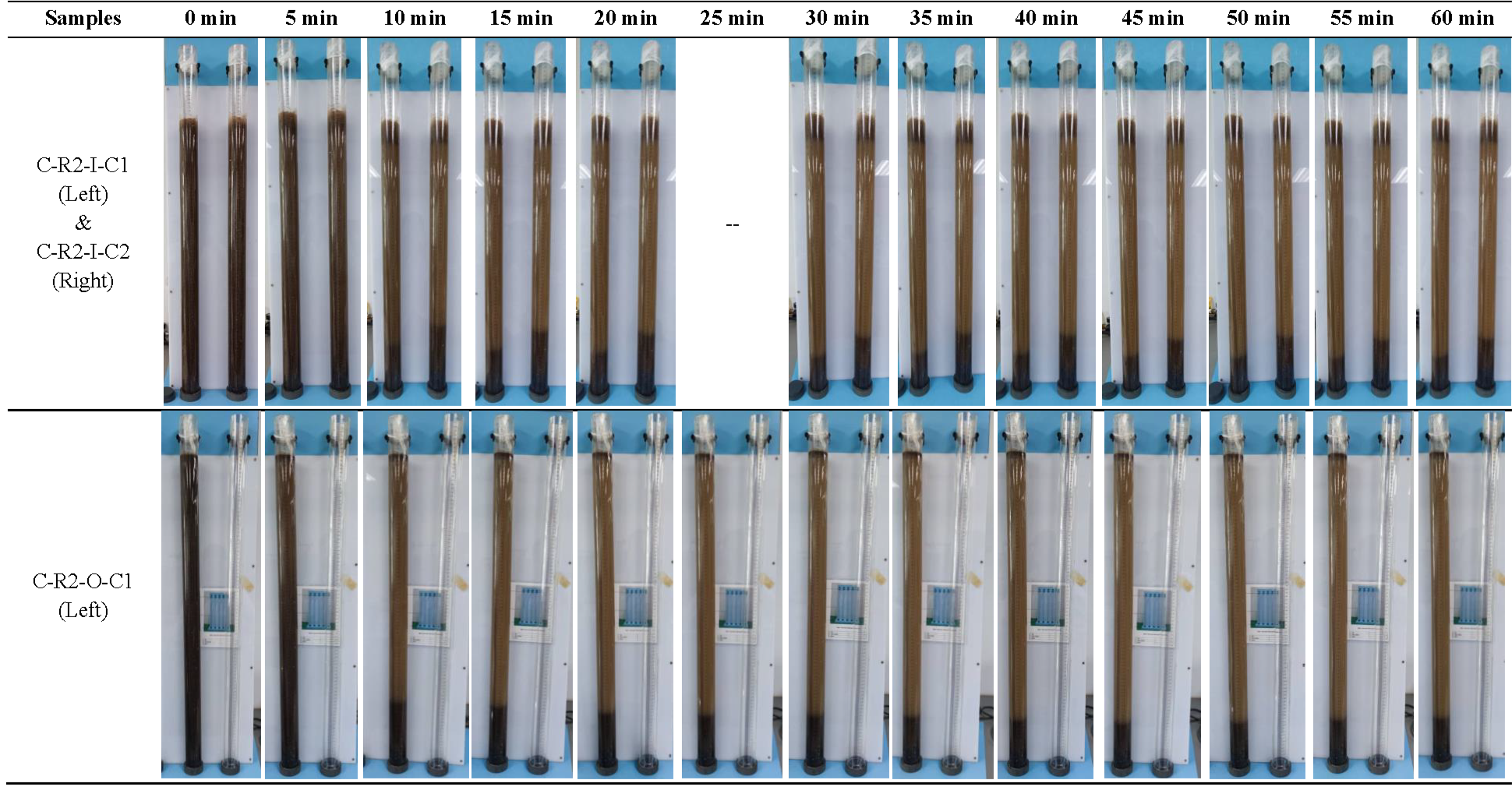


**Table S15.** Photographs of column tests (CTs) for Round 1 (R1) samples collected from Grease Traps D, including Column 1 (C1) for both inlet (I) and outlet (O) chamber samples and Column 2 (C2) for inlet (I) chamber samples only.


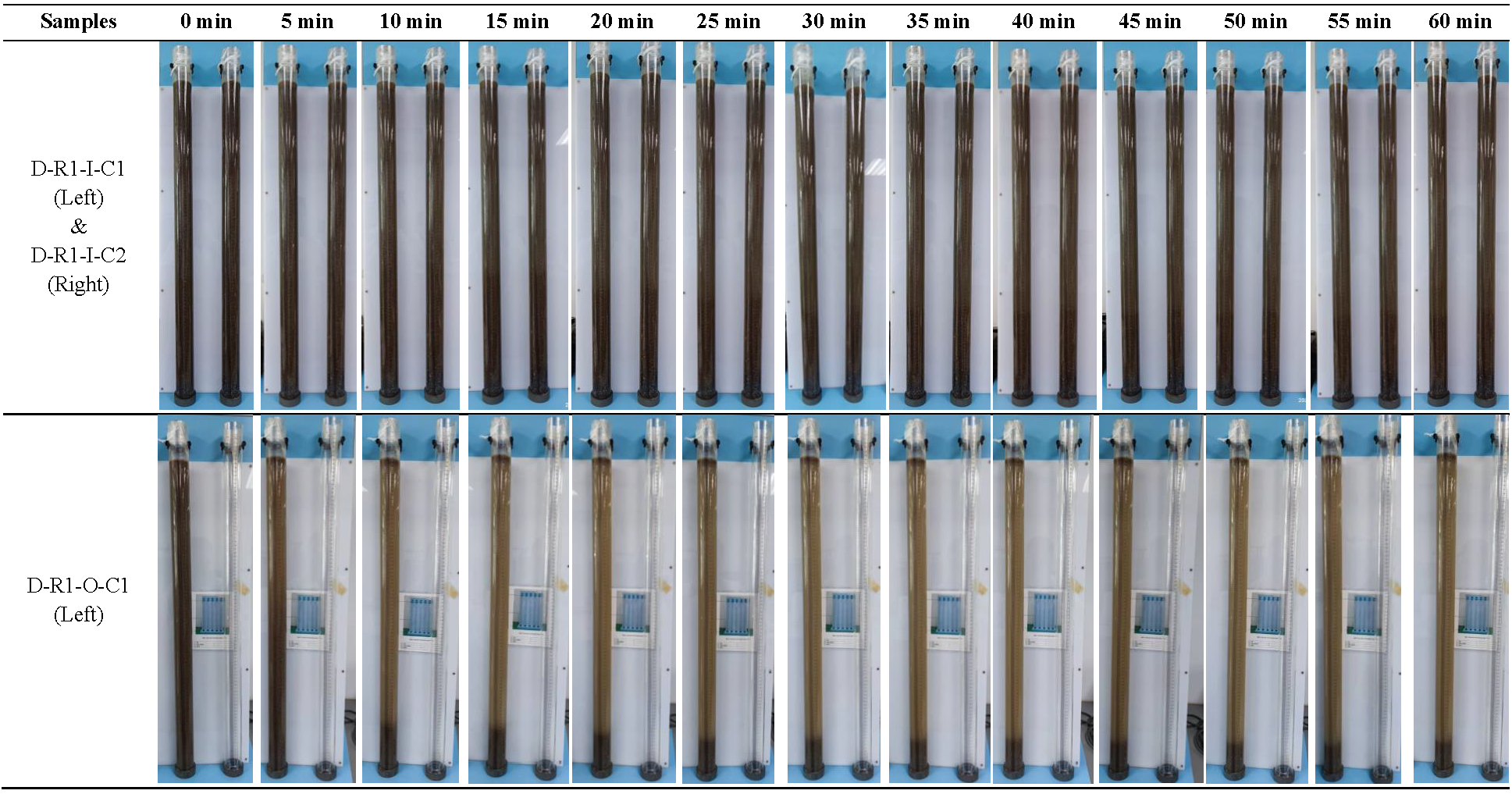


**Table S16.** Photographs of column tests (CTs) for Round 2 (R2) samples collected from Grease Traps D, including Column 1 (C1) for both inlet (I) and outlet (O) chamber samples and Column 2 (C2) for inlet (I) chamber samples only.


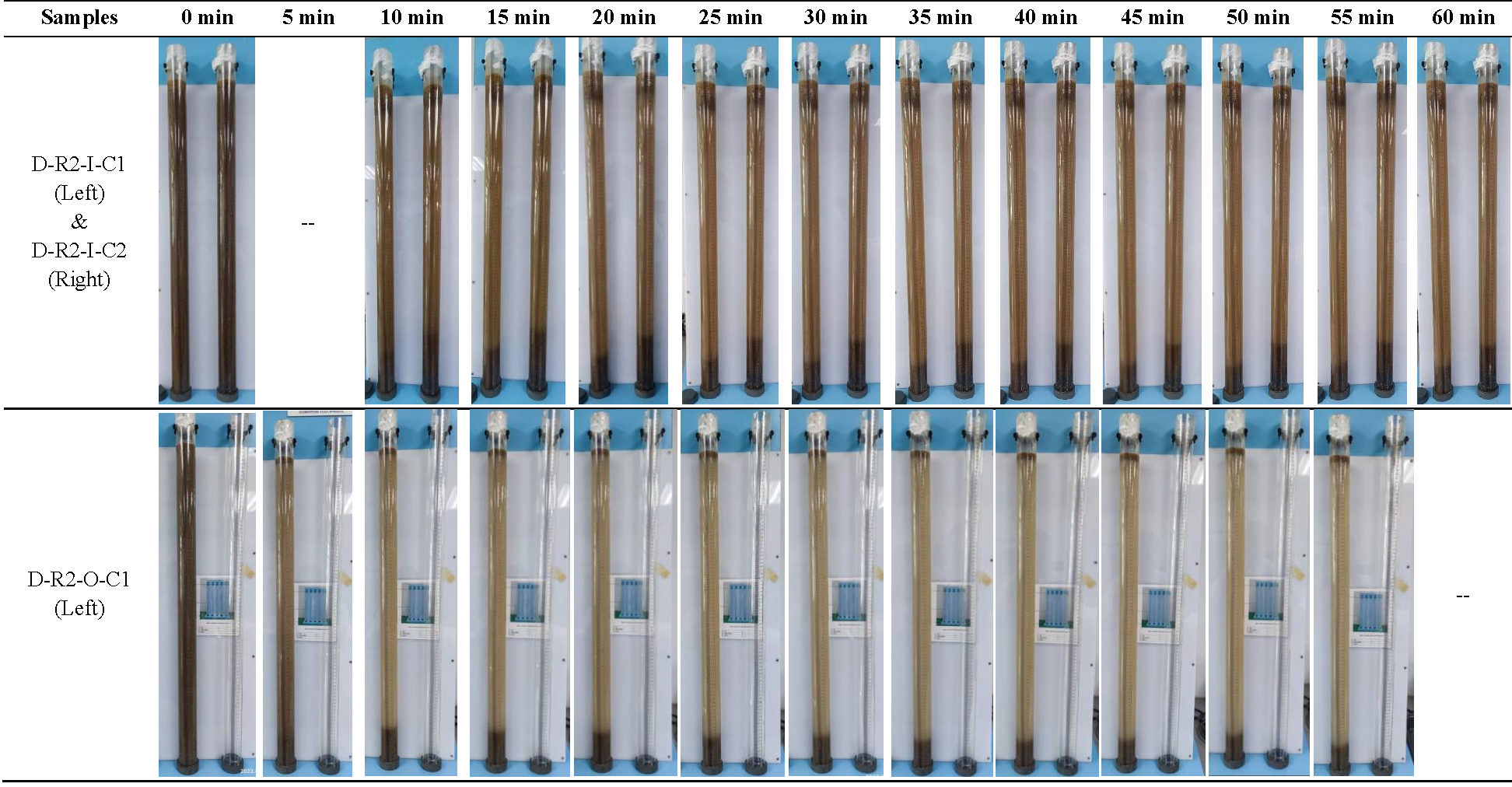


# References

Tadros, T. F. 2013. "Emulsion formation, stability, and rheology." *Emulsion Form. Stab.*: 1-75.10.1002/9783527647941.

Tang, L. Y., N. H. Wong, T. A. Chieng, A. K. J. Kiu, C. S. Choo, Y. Li, C. P. Tan, A. Z. Yaser, D. S. Khaerudini, G. H. Chen, and J. Sunarso. 2024. "Physicochemical characteristics of grease-trap wastewater with different potential mechanisms of FOG solid formation, separation, and accumulation inside grease traps." *Water Research*, 256: 121607.<https://doi.org/10.1016/j.watres.2024.121607>.
